# Strategic Data Re-Uploads: A Pathway to Improved Quantum Classification Data Re-Uploading Strategies for Improved Quantum Classifier Performance

**DOI:** 10.3390/e28050550

**Published:** 2026-05-13

**Authors:** Sara Aminpour, Yaser M. Banad, Sarah S. Sharif

**Affiliations:** 1 School of Electrical and Computer Engineering, University of Oklahoma, Norman, OK 73019, USA; sara.aminpour@ou.edu (S.A.); bana@ou.edu (Y.M.B.); 2 Center for Quantum Research and Technology, University of Oklahoma, Norman, OK 73019, USA; 3 Intelligent Neuromorphic and Quantum Understanding for Innovative Research and Engineering (INQUIRE) Laboratory, Norman, OK 73019, USA

**Keywords:** hybrid quantum machine learning, classification task, classical minimization, binary classification, data reuploading algorithm, single qubit classifier, entangled qubits

## Abstract

Quantum machine learning integrates quantum computing with classical machine learning techniques to enhance computational power and efficiency. A major challenge in quantum machine learning is developing robust quantum classifiers capable of accurately processing and classifying complex datasets. In this work, we present an advanced approach leveraging data re-uploading, a strategy that cyclically encodes classical data into quantum states to improve classifier performance. We examine two cost functions, fidelity and trace distance, across various quantum classifier configurations, including single-qubit, two-qubit, and entangled two-qubit systems. Additionally, we evaluate four optimization techniques (L-BFGS-B, COBYLA, Nelder–Mead, and SLSQP) to determine their effectiveness in optimizing quantum circuits for both linear and non-linear classification tasks. Our results show that the choice of optimization method significantly impacts classifier performance, with L-BFGS-B and COBYLA often yielding superior accuracy. The two-qubit entangled classifier shows improved accuracy over its non-entangled counterpart, albeit with increased computational cost. Also, the two-qubit entangled classifier is the best option for real-world random datasets in terms of accuracy and computational cost. Linear classification tasks generally exhibit more stable performance across optimization techniques compared to non-linear tasks. Our findings highlight the potential of data re-uploading in quantum machine learning, outperforming existing quantum classifier models in terms of accuracy and robustness. This work contributes to the growing field of quantum machine learning by providing a comprehensive comparison of classification strategies and optimization techniques in quantum computing environments, offering a foundation for developing more efficient and accurate quantum classifiers.

## 1. Introduction

Since its inception in 1959 [1], machine learning (ML) has become one of the most transformative technologies of the modern era, revolutionizing how we classify, cluster, and recognize patterns in vast datasets. Today, ML is deeply integrated into various sectors of society, and even small advancements in the field can yield significant economic and technological benefits. In recent years, a natural extension of ML has emerged within the framework of quantum mechanics, leading to the rise of Quantum Machine Learning (QML). By the mid-2010s [2], QML began gaining momentum as researchers explored the potential of quantum computing to enhance classical ML techniques [3,4]. Quantum computing leverages the principles of quantum mechanics, specifically entanglement, superposition, and interference, to execute computations [5]. Quantum information processing offers advantages in communication and computational tasks, such as solving algebraic problems, reducing sample complexity, and enhancing optimization processes. Notably, even simplified models of quantum computation can solve complex tasks, thereby holding promise for advancements in machine learning and artificial intelligence [6]. At the heart of contemporary QML practices is the training of quantum circuits, aimed at processing both classical and quantum [7,8,9,10,11,12,13].

In the emerging field of QML, quantum neural networks (QNNs) adapt this concept by leveraging quantum mechanics to process information [14,15]. These networks undergo a training process akin to their classical counterparts, where data is input into the quantum system, a cost function is computed based on the output, and the parameters of the QNN are iteratively adjusted through classical optimization techniques to minimize cost functions [16].

A notable advancement in quantum machine learning is the concept of data re-uploading, which cyclically encodes classical information into a quantum system and enables repeated incorporation of data throughout the quantum processing workflow [17,18]. Data re-uploading enables the construction of universal quantum classifiers [17,19], in which a quantum circuit is carefully organized into a sequence of stages dedicated to data integration and single-qubit operations [18,20]. Although several encoding approaches have been reported in the literature, including amplitude encoding [21,22], basis encoding [23], angle encoding [24,25,26,27,28], entangled feature maps [8,29], ZZ feature map [8,30,31], and N-local circuits [32], this study specifically focuses on data re-uploading, a promising method well-suited for practical deployment in future real-world applications [17,33,34]. This approach not only enhances the flexibility and adaptability of quantum classifiers but also significantly boosts their accuracy and efficiency in handling various classification tasks.

Several studies have explored various optimization techniques to enhance the performance of quantum classifiers [15,35,36,37]. Lockwood [38] presents a comprehensive empirical review of optimization techniques for quantum variational circuits, comparing 46 different optimizer setups, including minimization methods such as L-BFGS-B, Nelder-Mead, and SLSQP, across different QML problems such as Variational Quantum Eigensolver [39], Quantum Approximate Optimization Algorithm [40], and Moon binary classification [41]. Similarly, Lee et al. [42] propose an iterative layerwise optimization strategy for the quantum approximate optimization algorithm to reduce optimization costs while maintaining high approximation ratios. Their numerical simulations compare the performance of L-BFGS-B and Nelder–Mead optimizers in conjunction with the proposed strategy on the Max-cut problem. Although these studies provide valuable insights, there is still a lack of research comparing different cost functions, minimization methods, and classification patterns in combination with data reuploading techniques. The impact of data reuploading on the performance of various minimization methods remains largely unexplored. Further investigation into the interplay between data reuploading and different optimization techniques could potentially lead to more efficient and effective QML algorithms.

In addition, there is a notable gap in the literature considering random datasets that closely mimic real-world scenarios [38,42,43,44,45]. By using random datasets that approximate actual data, we can assess how well QML algorithms perform under conditions that are more representative of real-world applications, identify potential weaknesses or limitations in current QML techniques when faced with diverse and unpredictable data patterns, and develop more resilient and adaptable QML algorithms that can handle a wider range of data types and structures. Our initial results indicate that the proposed methodology shows promise when applied to a randomized dataset [46]. This encouraging outcome suggests that further investigation is warranted to validate the effectiveness of the methodology across a broader range of random datasets, assess its generalizability to various types of real-world data and applications, and compare its performance against existing QML techniques.

To comprehensively evaluate our proposed model, we are considering applying it to random datasets that simulate real-world data for potential applications. Additionally, we plan to conduct comparative analyses between fixed datasets and random datasets across various situations. This comparison will help us identify any discrepancies in the model’s performance between structured (fixed) and unstructured (random) data, assess the model’s ability to generalize across different data distributions and patterns, and determine the robustness of the model when faced with unexpected or noisy data. This approach will contribute to the development of more efficient, reliable, and versatile QML techniques that can address a wide range of practical challenges.

Moreover, we introduce the trace distance cost function as an alternative to the fidelity cost function, highlighting its distinct advantages in quantum classification tasks for the first time [47]. Unlike fidelity, which measures the overlap between quantum states, trace distance directly quantifies how distinguishable two states are, ranging from 0 (indistinguishable) to 1 (perfectly distinguishable) [48]. This makes it particularly effective for applications where state differentiation is crucial. Moreover, the trace distance cost function helps address the barren plateau problem, where gradients of random parameterized quantum circuits vanish exponentially with the number of qubits and layers [49,50,51]. This issue is especially pronounced with global cost functions like fidelity. By employing trace distance, the classifier becomes less prone to this vanishing gradient effect, providing a more stable and scalable training process. Through this exploration, we aim to assess the classifier’s adaptability and generalization potential under varying optimization criteria, offering valuable insights into its robustness and effectiveness across different conditions. Building on previous research that primarily examined the ‘L-BFGS-B’ method for fidelity cost function and fixed data set [19], this paper significantly expands the scope by incorporating three additional minimization techniques: ‘COBYLA,’ ‘Nelder–Mead,’ and ‘SLSQP’ for trace distance cost function, considering both fixed and random datasets. In this study, we employed three distinct optimization methods—COBYLA, Nelder–Mead, and SLSQP—in addition to L-BFGS-B to explore a range of approaches suited to different problem characteristics. COBYLA was chosen for its ability to handle non-linear constraints without requiring derivative information, making it versatile for complex constraint landscapes [52,53,54,55]. Nelder–Mead, a derivative-free method, was selected for its effectiveness with potentially non-smooth functions and simplicity in low-dimensional spaces [38,56,57,58]. SLSQP was included for its efficiency in handling both constrained and unconstrained problems, particularly when gradient information is available [59]. This diverse selection allows us to compare the performance of gradient-based and derivative-free methods, as well as those specialized for constrained optimization, providing a more comprehensive understanding of our problem’s optimization landscape than a single method like L-BFGS-B could offer. This dual evaluation of cost functions, fidelity and trace distance, allows for a more nuanced analysis of classifier behavior, revealing how different optimization methods interact with varied performance criteria.

Finally, we use linear classification patterns (LCP) and non-linear classification patterns (non-LCP) as a fundamental starting point for evaluating the performance of various optimization methods in quantum classifiers. This allows for a clear and controlled analysis of how quantum classifiers manage distinct types of data relationships. While more complex patterns can be studied, we consider fundamental line patterns for more intricate linear relationships and the circle pattern for more advanced non-linear patterns.

The structure of the paper unfolds as follows: The Section 2 presents a comprehensive analysis of our quantum classifier’s performance across 52 various configurations. We evaluate single-qubit, two-qubit, and two-qubit entangled classifiers using four optimization methods (L-BFGS-B, COBYLA, Nelder–Mead, and SLSQP, two cost functions (fidelity and trace distance), two classification patterns (LCP and non-LCP) as well as two datasets (fix and random). We present findings on accuracy, computational efficiency, and the impact of increasing layers and training samples. We examine the trade-offs between accuracy and computational cost, the advantages of entanglement in quantum classifiers, and the relative performance of different optimization methods across various classification tasks. This section also discusses the potential applications of our findings and their contribution to the broader field of quantum computing. Finally, the Section 4 elucidates our experimental approach, detailing the implementation of data re-uploading strategies, the construction of quantum circuits for different classifier types, and the specifics of our optimization techniques. We also describe our data generation processes for both fixed and random datasets and explain our evaluation metrics and statistical analysis methods.

In addition to the main manuscript, we have released, Supplemental Documents providing preliminary analysis to identify an appropriate number of training samples and layers, more details about the result for each section, detailed exploration of the process of reuploading, including how it occurs and is handled within the quantum classifier framework, methods and methodology of modeling cost functions, as well as minimization methods. We also released our main code for public use.

## 2. Results

In this study, we explored a range of models and methodological approaches to evaluate the performance of quantum classifiers in binary classification tasks. Two primary cost functions, fidelity and trace distance, were examined in combination with four optimization methods—L-BFGS-B, COBYLA, Nelder–Mead, and SLSQP—to enable a comprehensive assessment. In addition, we considered three quantum circuit configurations, single-qubit, two-qubit, and entangled two-qubit systems, to examine performance differences across distinct quantum architectures.

Both fixed and random datasets were considered to evaluate the robustness and adaptability of the classifiers. We generate a random dataset for non-LCP on a plane with coordinates $\vec{x}=(x_{1},x_{2})$ with $x_{i}\in [-1,1]$ defined by $x_{1}^{2}+x_{2}^{2}<r^{2}$, aiming to classify these data based on whether they fall inside or outside a circle of radius $r=\sqrt{\frac{2}{\pi }}$. The radius is chosen in a way that ensures equal areas for the regions inside and outside the circle. This setup results in a balanced dataset, where randomly assigning labels to data points would yield a 50 percent accuracy rate by chance. To ensure uniformity across our experiments, a consistent seed was utilized for generating all data points when dataset is fixed. Conversely, for analyses involving random data, data points were generated entirely at random for each of the 20 iterations to ascertain the average accuracy.

The outcomes of these runs were averaged to present a more reliable and statistically significant assessment of the classifiers’ performance. This approach enabled us to evaluate the quantum classifiers across diverse scenarios, capturing their true capabilities in both stable and unpredictable data environments.

**Figure 1:**
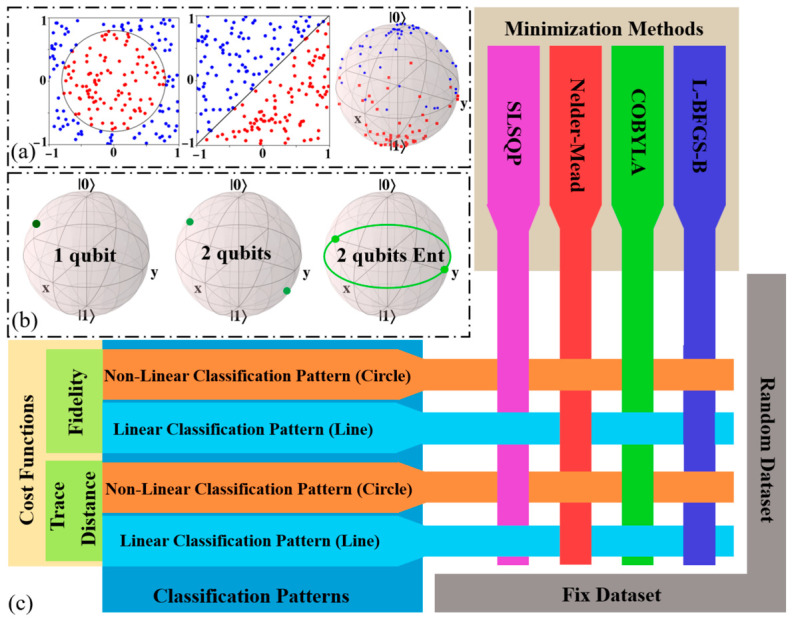
(**a**) Linear and non-linear classification pattern represented by a circle and line, and mapping of data points onto the Bloch sphere for quantum classification. Blue and red points represent different classes. (**b**) Illustrations of states on the Bloch sphere for a single qubit, two qubits, and two qubits entangled. On each Bloch sphere, x and y denote the equatorial Cartesian axes, while |0⟩ and |1⟩ denote the computational basis states of a qubit, located at the north and south poles, respectively. For the two-qubit configurations, the spheres show the reduced single-qubit states obtained by tracing out the second qubit. (**c**) Visualization of quantum classification concepts and schematics of 52 different cases studied in this paper. The figure outlines two cost functions, two classification patterns, and four minimization methods under two fixed and random datasets.

We studied all models with a training sample size of up to 250, and we eliminated the results of overfitting, so the results presented vary from 50 to 250. This careful selection of data points ensured that the findings accurately reflected the performance of the classifiers without being skewed by overfitting. A conceptual overview of the studied models and methodologies is provided in Figure 1, illustrating the key components of this investigation.

Our next focus was on benchmarking the selection of classifiers across varying numbers of layers, with a particular emphasis on configurations comprising five layers. This emphasis was based on the hypothesis that a five-layer architecture could potentially achieve enhanced performance and accuracy.

The subsequent sections delve deeper into this exploration, providing detailed insights into the performances of specific algorithms when implemented using single-qubit and 2-qubit classifiers with the innovative technique of data re-uploading. This methodical approach not only enhances our understanding of the quantum classifier’s potential but also sets the stage for future advancements in the field of QML, spotlighting the critical role of algorithmic diversity and adaptability in navigating the complexities of quantum data classification.

Before delving into the accuracy metrics of the 52 unique scenarios depicted in Figure 1, we embarked on a preliminary analysis to identify an appropriate number of training samples and layers. This preparatory step was crucial not only for establishing a consistent baseline for comparing training and test accuracies across various configurations but also for ensuring that our simulations remained feasible on our desktop computer with limited configurations. As illustrated in Figures S1.1 and S1.2 in the Supplementary Note S1, we conducted a series of runs with our algorithm, varying the number of layers from 1 to 5 and using up to 250 training samples, to determine the conditions under which our algorithm would reach a test accuracy around 90%. This exploration led us to conclude on selecting 5 layers of training for our study. To maintain a uniform evaluation framework, we subsequently used these values for all simulated cases.

### 2.1. Evaluating Linear and Non-Linear Classification Approaches for Fidelity in Fixed and Random Datasets for a 1-Qubit Classifier for Four Different Minimization Methods

In our study, we devised a methodology to assess the performance of a single-qubit classifier across various conditions by constructing training datasets of varying sizes. For certain minimization methods, we employed different ranges of sample sizes to effectively control for overfitting, ensuring that the results accurately reflected the classifier’s true performance.

The classifier’s efficacy was then evaluated using a comprehensive test dataset consisting of 4000 data points.

**Figure 2:**
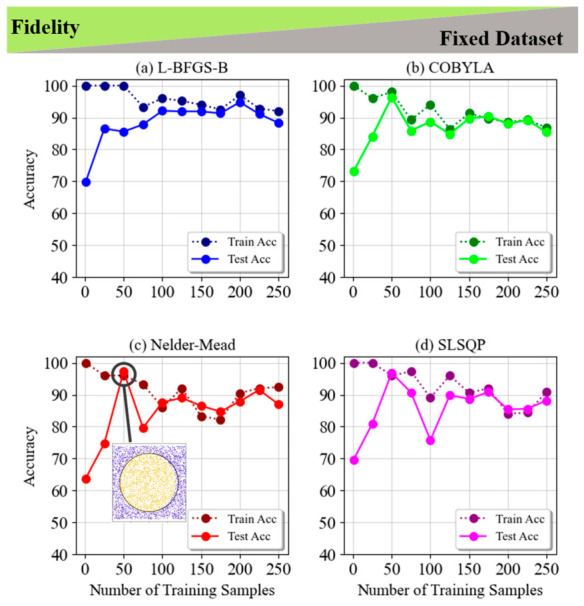
Train and test accuracy of fidelity for the 5-layer model of non-LCP and fixed dataset for (**a**) L-BFGS-B, (**b**) COBYLA, (**c**) Nelder–Mead and (**d**) SLSQP minimization methods. The inset image in subplot (**c**) in the graph shows a visualization of a circle classification task with the highest accuracy of 97.3% in the Nelder–Mead minimization method.

**Figure 3:**
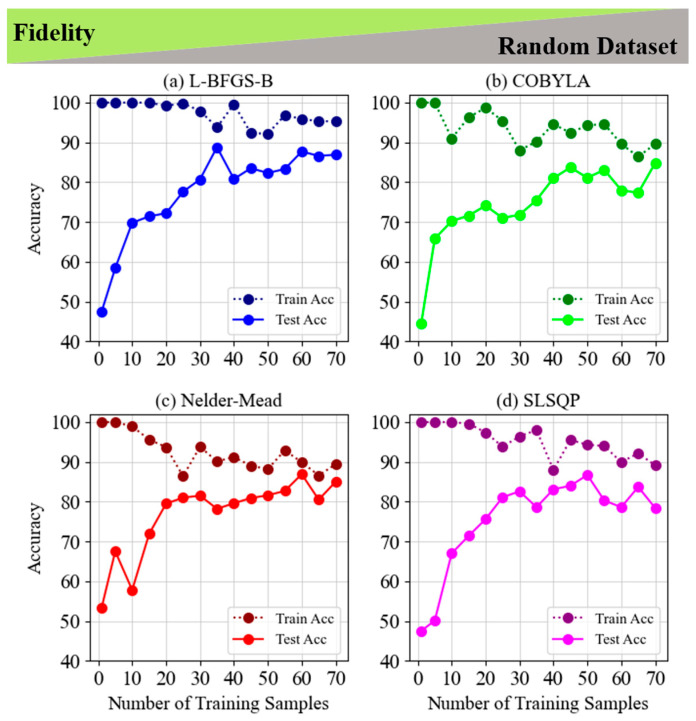
Train and test accuracy of fidelity for the 5-layer model of non-LCP and random dataset for (**a**) L-BFGS-B, (**b**) COBYLA, (**c**) Nelder–Mead and (**d**) SLSQP minimization methods.

This section is focused on the Fidelity cost function, and we presented the comparison of four optimization techniques (L-BFGS-B, COBYLA, Nelder–Mead, and SLSQP) for different datasets and classification patterns. Figure 2 and Figure 3 show the results for the non-LCP for fixed and random datasets, respectively. Initially, all methods achieve perfect training accuracy, but L-BFGS-B shows greater resistance to overfitting, maintaining high training accuracy as the sample size increases. In contrast, COBYLA, Nelder–Mead, and SLSQP display higher susceptibility to overfitting and performance fluctuations, especially in test accuracy. Notably, L-BFGS-B achieves peak accuracy with more training samples, while the others perform better with fewer samples, underscoring the importance of careful sample selection. These insights emphasize the need to balance the number of training samples and method choice to optimize accuracy and prevent overfitting.

**Figure 4:**
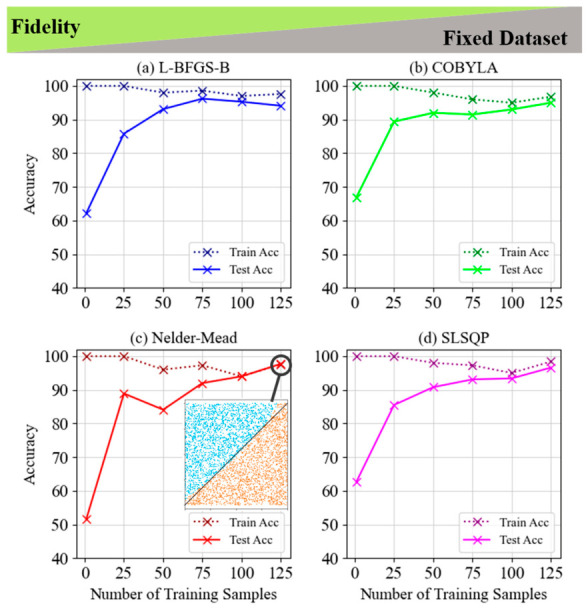
Train and test accuracy of fidelity for the 5-layer model of LCP and fixed dataset for (**a**) L-BFGS-B, (**b**) COBYLA, (**c**) Nelder–Mead and (**d**) SLSQP minimization methods. The inset graph in subplot (**c**) shows the visualization of a line classification pattern with the highest accuracy of 97.7% in the Nelder–Mead minimization method.

**Figure 5:**
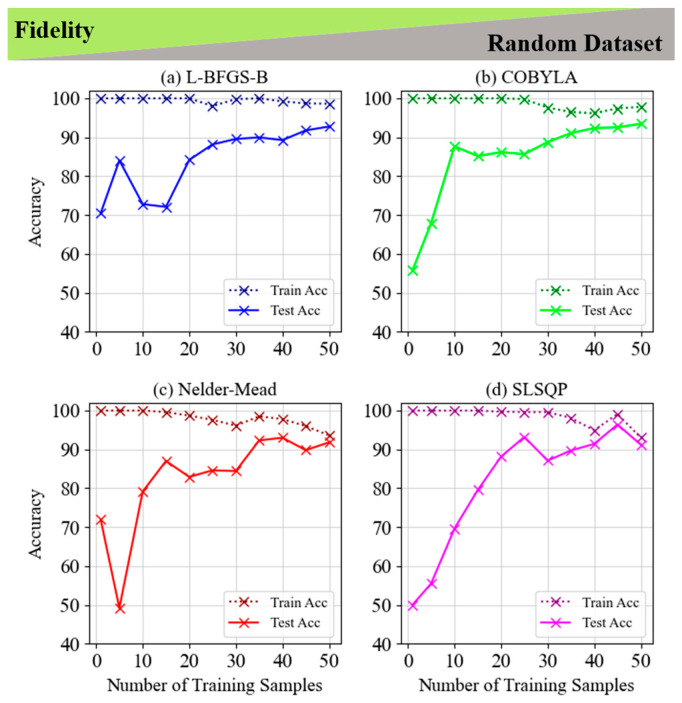
Train and test accuracy of fidelity for the 5-layer model of LCP and random dataset for (**a**) L-BFGS-B, (**b**) COBYLA, (**c**) Nelder–Mead and (**d**) SLSQP minimization methods.

Figure 4 and Figure 5 compare four optimization techniques (L-BFGS-B, COBYLA, Nelder–Mead, and SLSQP) for classifying LCP using the fidelity cost function, fixed and random datasets. In Figure 4, all methods achieve near-perfect training accuracy, with test accuracy improving as the number of training samples increases, peaking around 94–98% with 125 samples. L-BFGS-B and COBYLA show a reduction in the training-test accuracy gap as sample size grows, while Nelder–Mead achieves precise classification with minimal gaps between train and test accuracies. Figure 5 demonstrates a similar trend on random datasets, with all methods surpassing 90% accuracy with 50 training samples. COBYLA and SLSQP exhibit superior generalization, while Nelder–Mead achieves the smallest gap between train and test accuracy for 50 training samples, underscoring its balance between training and generalization. Both figures highlight the importance of selecting an optimal number of training samples for effective performance and overfitting mitigation across methods.

A comparison of Figure 2 and Figure 4 highlights that LCP exhibits more stable and consistent accuracy curves across all optimization techniques, while non-LCP shows greater variability and fluctuations. This difference may arise from several factors: (1) LCP likely aligns better with linear decision boundaries, making it easier for classifiers to generalize, whereas non-LCP involves more complex, non-linear patterns that challenge generalization and lead to overfitting or underfitting. (2) The algorithms’ adaptability to specific classification tasks may also affect their performance. Similarly, Figure 3 and Figure 5 reveal that the classification problem’s complexity impacts accuracy, with LCP requiring fewer samples for stable accuracy, while non-LCP fluctuates more, underscoring the importance of matching optimization methods to problem complexity. We explained more about the non-linear and linear classification approaches for fidelity in fixed and random datasets for the 1-qubit classifier in Supplementary Note S2.

### 2.2. Evaluating Linear and Non-Linear Classification Approaches for Trace Distance in Fixed and Random Datasets for a 1-Qubit Classifier for Four Different Minimization Methods

**Figure 6:**
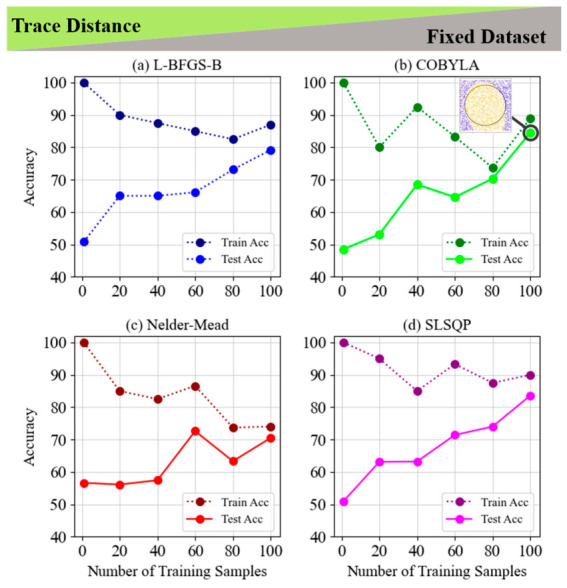
Train and test accuracy of trace distance for the 5-layer model of non-LCP and fixed dataset for (**a**) L-BFGS-B, (**b**) COBYLA, (**c**) Nelder–Mead and (**d**) SLSQP minimization methods. The inset graph in subplot (**b**) shows the visualization of a circle classification pattern with the highest accuracy of 84.6% in the COBYLA minimization method.

**Figure 7:**
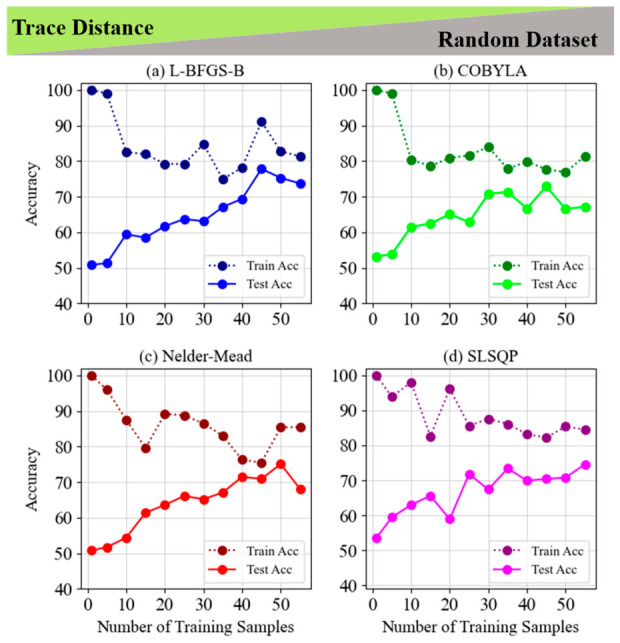
Train and test accuracy of trace distance for the 5-layer model of non-LCP and random dataset for (**a**) L-BFGS-B, (**b**) COBYLA, (**c**) Nelder–Mead and (**d**) SLSQP minimization methods.

Figure 6 and Figure 7 examine the use of the trace distance cost function for classifying non-LCP on fixed and random datasets, comparing four optimization techniques: L-BFGS-B, COBYLA, Nelder–Mead, and SLSQP. In Figure 6, all methods achieve perfect training accuracy with relatively few samples, but their test accuracy varies. COBYLA performs best, reaching 84.6% with 100 samples and demonstrating strong generalization. L-BFGS-B achieves 79.2%, while Nelder–Mead and SLSQP show more variability, with Nelder–Mead experiencing overfitting. Figure 7 reveals a similar trend on random datasets, with test accuracy improving as the number of training samples increases. L-BFGS-B reaches the highest test accuracy (77.8%) with 45 samples, followed by COBYLA at 72.9%, and SLSQP and Nelder–Mead showing moderate fluctuations. Overall, COBYLA excels in generalization for fixed datasets, while L-BFGS-B stands out for random datasets. Both figures highlight the importance of selecting appropriate optimization methods and training sample sizes based on the task complexity and dataset type.

**Figure 8:**
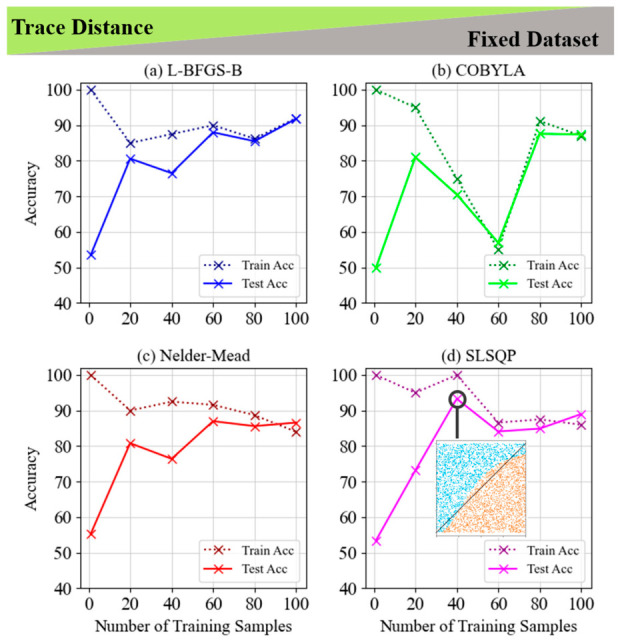
Train and test accuracy of trace distance for the 5-layer model of LCP and fixed dataset for (**a**) L-BFGS-B, (**b**) COBYLA, (**c**) Nelder–Mead and (**d**) SLSQP minimization methods. The inset graph in subplot (**c**) shows the visualization of a line classification pattern with the highest accuracy of 93.3% in the SLSQP minimization method.

**Figure 9:**
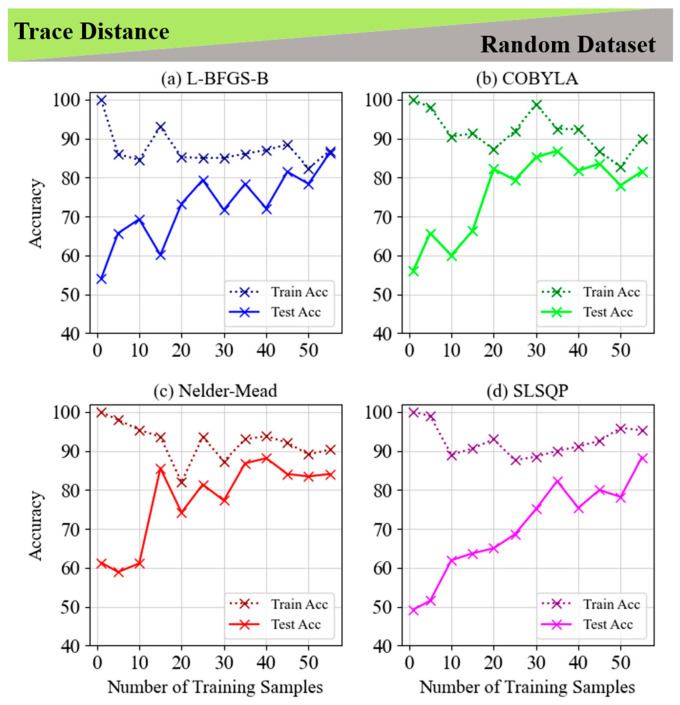
Train and test accuracy of trace distance for the 5-layer model of LCP and random dataset for (**a**) L-BFGS-B, (**b**) COBYLA, (**c**) Nelder–Mead and (**d**) SLSQP minimization methods.

Figure 8 and Figure 9 compare the performance of four optimization methods—L-BFGS-B, COBYLA, Nelder–Mead, and SLSQP—for LCP using a trace distance cost function across both fixed and random datasets. In both figures, SLSQP stands out, achieving the highest test accuracy with minimal overfitting and consistent generalization, peaking at 93.3% in the fixed dataset and 88.3% in the random dataset. L-BFGS-B also shows strong performance, particularly with larger datasets, while COBYLA and Nelder–Mead exhibit fluctuations and signs of overfitting as the number of training samples increases for both datasets. Overall, SLSQP is the most robust method, handling both datasets effectively and maintaining accuracy without overfitting. For a more detailed analysis of the LCP and non-LCP approaches using the trace distance cost function with fixed and random datasets for the 1-qubit classifier, please refer to Supplementary Note S3. Also, for a comprehensive performance comparison of 5-layer single-qubit quantum classifiers using fidelity and trace distance cost functions across various classification tasks and dataset types, please refer to Supplementary Note S4.

### 2.3. Evaluating Non-Linear and Linear Classification Approaches for Fidelity in Fixed and Random Datasets for 2-Qubit and 2-Qubit Entangled Classifiers

**Figure 10:**
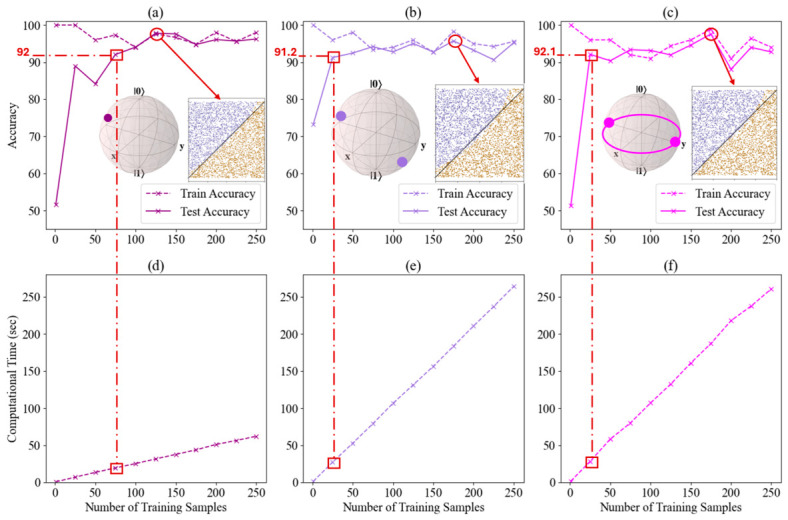
Comparative analysis of quantum classifiers for LCP using single-qubit, two-qubit, and two-qubit entangled systems for Nelder–Mead minimization method. The upper panels (**a**–**c**) display train and test accuracies as a function of training sample size, showcasing robust classification performance across all quantum configurations. In panels (**a**–**c**) the red circle highlights the highest test accuracy achieved, while the red square marks the first training-sample size at which the test accuracy exceeds 90%. The red dashed vertical line drops from the red square in each upper panel to the corresponding lower panel (**d**–**f**), indicating the computational time required to first reach a test accuracy above 90%. The Bloch-sphere insets in panels (**a**–**c**) illustrate the qubit configuration used in each case, where x and y denote the equatorial Cartesian axes of the Bloch sphere, and |0⟩ and |1⟩ denote the computational basis states of a qubit, located at the north and south poles, respectively. The lower panels (**d**–**f**) present the relationship between computational time and the number of training samples, highlighting a substantial increase in computational complexity for two-qubit systems relative to the single-qubit implementation. This comprehensive evaluation elucidates the balance between classification accuracy and computational efficiency in quantum machine learning approaches.

Building on our findings from Figure 4, where we analyzed four distinct minimization methods, we identified that the Nelder–Mead minimization method achieved the highest accuracy of 97.3% when assessed using both fidelity and trace distance cost functions for LCP. This observation prompted us to extend our investigation to 2-qubit and 2-qubit entangled systems, focusing specifically on the fidelity cost function in combination with the Nelder–Mead minimization method. To assess the efficiency of these classifiers for LCP, we analyzed the computational time required to achieve the highest accuracy using the fidelity cost function and the Nelder–Mead minimization method across 1-qubit, 2-qubit, and 2-qubit entangled classifiers. This approach allowed us to gain a comprehensive understanding of the performance and computational efficiency of the fidelity cost function paired with the Nelder–Mead minimization method across various quantum configurations. Figure 10a–c presents a comparative analysis of accuracy in quantum classifiers for LCP using three different quantum systems: single-qubit, two-qubit, and two-qubit entangled configurations, while Figure 10d–f represents the computational time vs. number of training samples. Figure 10a, the single-qubit system shows a steep learning curve, with accuracy rising from 51.6% to 92% after just 75 training samples, and stabilizing between 92% and 97.7% as the sample size increases to 250. To assess the computational efficiency of the quantum classifier, we extended the original implementation provided by Pérez-Salinas et al. [60] with the datetime library in Python (version 3.13.1; Python Software Foundation, Wilmington, DE, USA). Our modified version incorporates time measurement commands to quantify the runtime of the classification algorithm across various problem instances and classifier configurations. This enhancement allows us to analyze the computational cost of the quantum approach. As shown in Figure 10d, its computational time reaches 62.15 s for 250 samples, making it both accurate and computationally efficient. In contrast, Figure 10b presents the 2-qubit classifier, which starts with higher accuracy (73.2%) and gradually peaks at 95.7% with 175 samples, but with a significantly higher computational time of 260 s for 250 samples, indicated in Figure 10e. Figure 10c illustrates the performance of the 2-qubit entangled classifier, which, while achieving the highest peak accuracy (97.5%), also exhibits pronounced fluctuations in accuracy and matches the non-entangled system’s computational cost of 260 s in Figure 10f. This comparison highlights trade-offs: the single-qubit system offers stability and efficiency, the 2-qubit classifier provides robust initial accuracy with more resource demands, and the 2-qubit entangled system offers the highest peak accuracy but at the cost of increased computational complexity and performance variability. The choice of system depends on whether stability, efficiency, or peak performance is prioritized.

**Figure 11:**
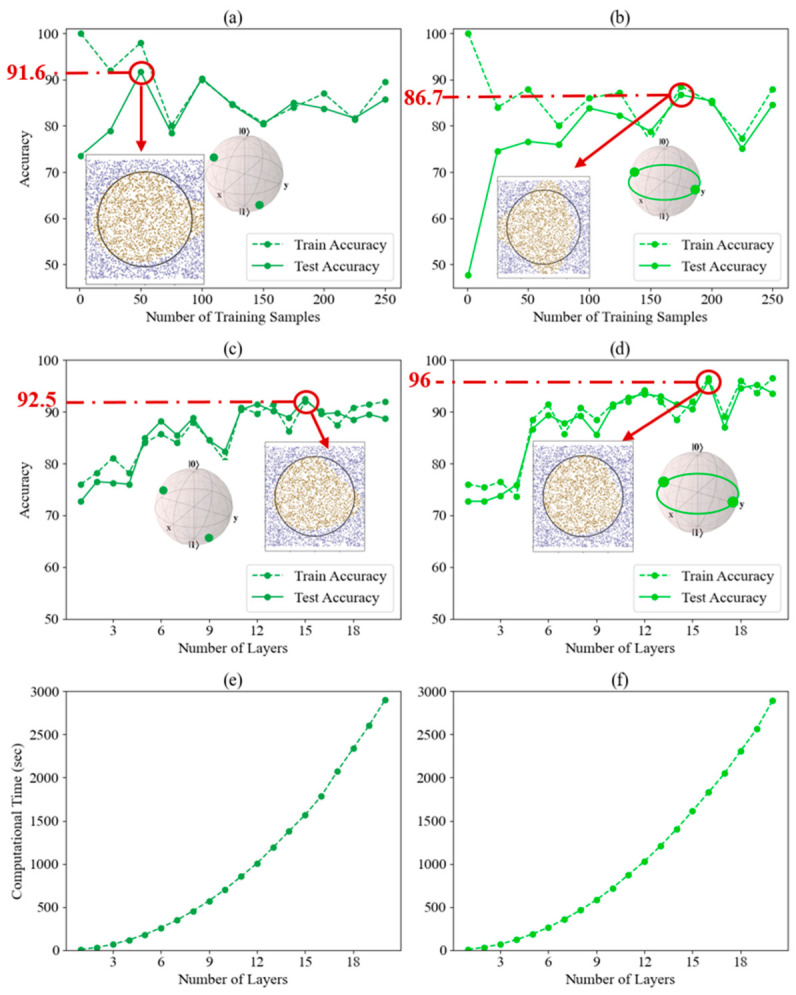
Performance analysis of 2-qubit and 2-qubit entangled classifiers for non-linear classification with Nelder–Mead minimization method for fixed dataset. (**a**,**b**) Accuracy vs. number of training samples. (**c**,**d**) Accuracy vs. number of layers. (**e**,**f**) Computational time vs. number of layers. Left column (**a**,**c**,**e**) shows results for the 2-qubit classifier, right column (**b**,**d**,**f**) for the 2-qubit entangled classifier. Subplots (**c**–**f**) use 175 training samples based on the accuracy convergence observed in (**a**,**b**). In panels (**a**–**d**) the red circle highlights the data point with the highest test accuracy.

For the one-qubit case, our results indicate that the highest accuracy is achieved when the number of layers is set to 5. This configuration was therefore maintained as the initial condition for both the 2-qubit and 2-qubit entangled classifiers. We then explored the optimal number of training samples required to achieve the highest accuracy without inducing overfitting, identifying 175 samples as the threshold for the fixed dataset. We then incrementally increased the number of layers from 1 to 20, using the optimal training sample size of 175, as illustrated in Figure 11. Figure 11a,b reveal that both classifiers improve in train and test accuracy as training samples increase, with the 2-qubit classifier showing higher initial test accuracy (73.5%) and more stable performance, while the 2-qubit entangled classifier starts lower (47.6%) but improves significantly with more samples. Figure 11c,d demonstrate that increasing the number of layers in the quantum circuit boosts accuracy for both classifiers, though the 2-qubit entangled classifier benefits more dramatically, especially early on. Both classifiers plateau in performance after 12–15 layers. Figure 11e,f show that computational time grows exponentially with the number of layers for both classifiers, reflecting a similar scaling trend regardless of entanglement use. The 2-qubit classifier generally achieves better accuracy with fewer training samples and maintains stable performance, while the 2-qubit entangled classifier, though more volatile, demonstrates greater potential for capturing complex patterns with more layers and samples. However, this advantage comes with higher computational costs and sensitivity to changes in training conditions.

**Figure 12:**
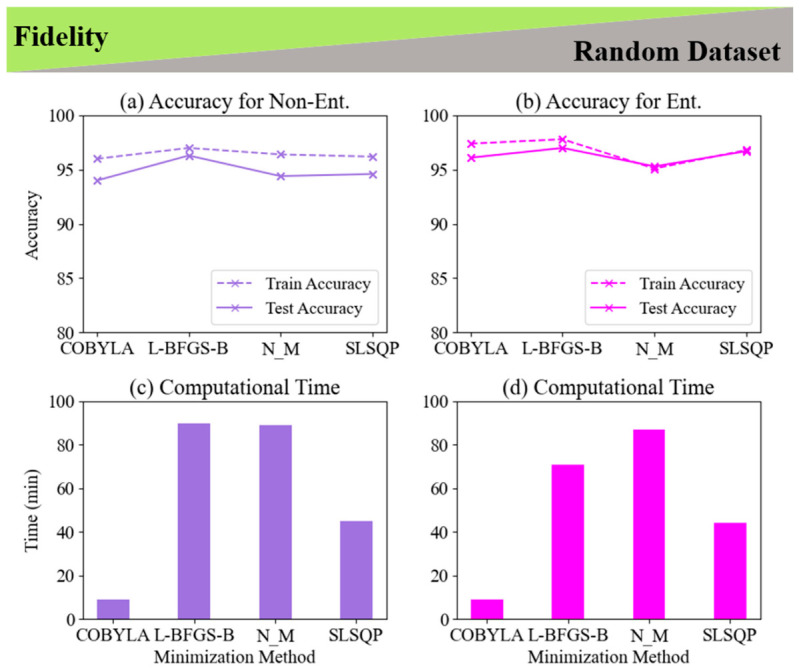
Performance analysis of 2-qubit quantum classifiers for linear classification using a random dataset with 250 training samples. (**a**) Accuracy for the 2-qubit classifier, (**b**) Accuracy for the 2-qubit entangled classifier, (**c**) Computational time for 2-qubit classifier, and (**d**) Computational time for 2-qubit entangled classifier. Results compare four optimization methods (COBYLA, L-BFGS-B, NELDER–MEAD, SLSQP) using a fidelity cost function, illustrating trade-offs between accuracy and computational efficiency.

**Figure 13:**
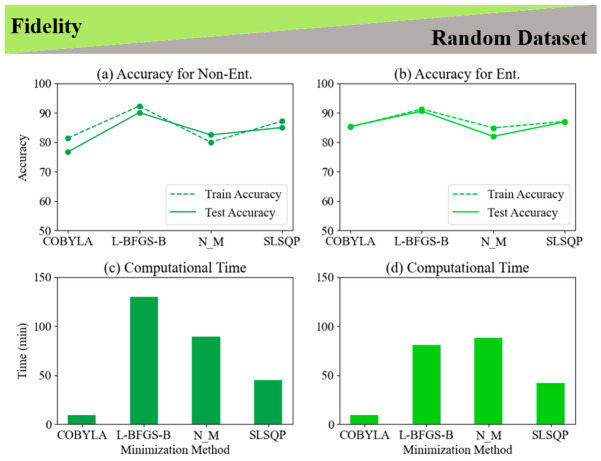
Performance comparison of 2-qubit quantum classifiers for non-linear classification using a random dataset with 250 training samples. (**a**) Accuracy for non-entangled classifier, (**b**) Accuracy for entangled classifier, (**c**) Computational time for non-entangled classifier, and (**d**) Computational time for entangled classifier. Results compare four optimization methods (COBYLA, L-BFGS-B, NELDER–MEAD, SLSQP) using a fidelity cost function, demonstrating the trade-offs between classification accuracy and computational efficiency for non-linear (circular) decision boundaries.

The exploration of random datasets within the context of data reuploading has not been previously reported, prompting us to address this gap in the literature. To ensure a comprehensive analysis, we examined the fidelity cost function alongside four minimization methods: COBYLA, L-BFGS-B, Nelder–Mead, and SLSQP. This evaluation was conducted for both LCP and non-LCP scenarios, focusing on 2-qubit and 2-qubit entangled classifiers. To assess the effectiveness of these minimization methods, we also analyzed the associated computational time for each case. Figure 12 and Figure 13 present a comparison of the train and test accuracy, as well as computational time for four optimization methods (COBYLA, L-BFGS-B, NELDER_MEAD, and SLSQP). Concluding the result from Figure 10 and Figure 11, we kept the number of training samples and the number of layers constant at 250 and 5, respectively. Figure 12 shows the results when we applied the fidelity cost function to the LCP task and a random dataset using 2-qubit and 2-qubit entangled classifiers. The results show, on average, the 2-qubit entangled classifier achieves approximately 2% higher test accuracy than the non-entangled classifier. In addition, L-BFGS-B not only has the highest test accuracy in comparison with 1-qubit (Figure 5), but it also provides the highest accuracy for both 2-qubit and 2-qubit entangled for the amount of 96.3% and 97%, respectively.

### 2.4. Evaluating Non-Linear and Linear Classification Approaches for Fidelity in Fixed and Random Datasets

Comparing the computational time, remarkably, the COBYLA minimization method completed the tasks for 2-qubit and 2-qubit entangled in just 9 min for both classifiers. While the COBYLA minimization method achieved the lowest test accuracy (94% for non-entangled and 95.3% for entangled), its computational time was approximately 10 times faster than the L-BFGS-B and Nelder–Mead methods, and 5 times faster than the SLSQP method, making it the fastest option for evaluating random datasets using the fidelity cost function and random dataset for data reuploading with a 2-qubit classifier. In contrast, L-BFGS-B and NELDER MEAD take the most time, with 90 and 89 min for the non-entangled classifier, respectively, and reduced times of 71 and 87 min for the entangled version, respectively. This suggests that the 2-qubit entangled classifier offers better overall performance and generalization but requires more computational resources, highlighting the trade-off between accuracy and time in selecting the optimal minimization method in the presence of real data analysis. Figure 13 presents a comparison of four optimization methods (COBYLA, L-BFGS-B, NELDER_MEAD, and SLSQP) for non-LCP using 2-qubit and 2-qubit entangled quantum classifiers, evaluating the accuracy and computational time with 250 training samples. In terms of accuracy, the 2-qubit classifier shows L-BFGS-B as the best performer, exceeding 90% in both train and test accuracies, while COBYLA has the lowest test accuracy at 76.7%. NELDER_MEAD and SLSQP offer intermediate results, with test accuracies between 82–87%. The 2-qubit entangled classifier shows improved accuracy overall, with L-BFGS-B still leading, and COBYLA notably improving to 85.4%, with smaller accuracy gaps between training and testing, indicating better generalization. On the computational side, COBYLA is the fastest for both classifiers, taking just 9 min, while L-BFGS-B is the slowest for the 2-qubit classifier at 130 min, but reduces to 81 min in the entangled system. NELDER_MEAD and SLSQP show moderate times, with SLSQP remaining stable across both classifiers at around 42–45 min. The analysis highlights the trade-offs between accuracy and computational time, with the 2-qubit entangled classifier offering superior overall performance. L-BFGS-B provides the highest accuracy but with greater computational cost, while COBYLA is more efficient, making it a balanced choice for quantum classification tasks, especially in entangled systems. A detailed examination of LCP and non-LCP approaches for fidelity in fixed and random datasets for 2-qubit and 2-qubit entangled classifiers is provided in Supplementary Note S5.

## 3. Discussion

This work presents a pioneering investigation into enhancing quantum classifier performance through strategic data re-uploading, exploring its impact across both linear and non-linear classification patterns. Although we focused on straight-line and circular decision boundaries as representative linear and nonlinear cases, the universality of the data-reuploading ansatz ensures that—with a sufficient number of layers—it can approximate arbitrarily complex separatrices. Straight lines and circles give us a solid foundation for understanding linear and nonlinear data spreads. For even more complex or higher-dimensional patterns, future work should look at adaptive, layer-by-layer encoding schemes and smarter, geometry-aware circuit designs. By integrating novel cost functions and employing various new optimization methods, we significantly advanced the accuracy and robustness of quantum classifiers. Our approach, which leverages the unique properties of quantum mechanics, demonstrates substantial improvements over traditional models, particularly in handling complex patterns within minimal datasets. Classical models such as neural networks and SVMs typically require over 1000 training samples to reach comparable accuracy on classification tasks [61,62,63]. In contrast, our quantum classifier achieves similar performance with as few as 250 samples, demonstrating superior sample efficiency. Through comprehensive comparisons across diverse datasets and classification tasks, we underscore the adaptability of our methodology to different learning scenarios, thereby offering a versatile tool for QML applications.

Our findings contribute to the theoretical foundations of QML while also providing practical insights into the design and optimization of quantum classifiers. The comparison of different cost functions demonstrates that each influences model performance in a distinct manner, underscoring the importance of selecting an appropriate objective function for a given task. Furthermore, our results highlight the effectiveness of data re-uploading in enhancing model expressivity, which is a critical factor in achieving high classification accuracy with relatively few training samples, particularly when representing real-world datasets.

Future work will focus on extending these methodologies to more complex quantum systems and exploring their application in broader quantum computing tasks. By continuing to unravel the capabilities of quantum classifiers and refining their design, we move closer to realizing the full potential of quantum computing in addressing some of the most challenging problems in machine learning and beyond. Our study also initialized the fastest method with respect to the minimization method, a number of qubits, and the present data sets, which show promising results using the introduced methods for real-world data sets, which we will consider for our next research work.

This research not only paves the way for further advancements in QML but also highlights the transformative impact quantum computing can have across various scientific and technological domains.

## 4. Method

**Figure 14:**
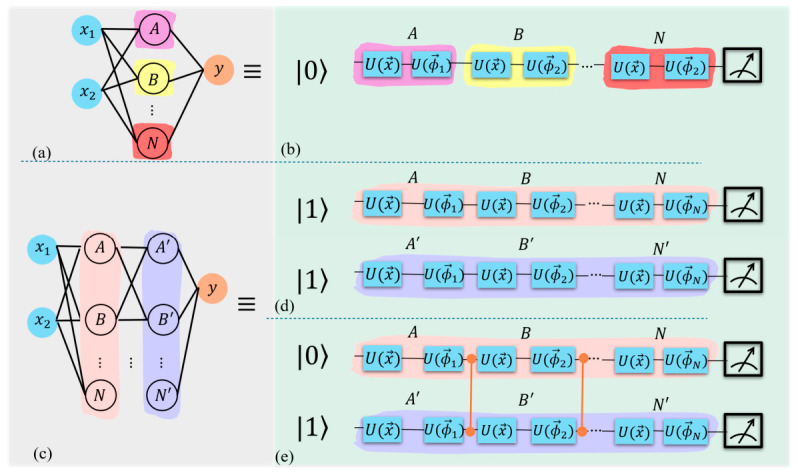
Comparison of classical neural networks and their quantum counterparts for classification tasks. (**a**) Classical neural network with one hidden layer (gray background) and (**b**) its single-qubit quantum circuit equivalent with 2 processing units (green background). The quantum circuit shows alternating data upload U(x) and learnable unitary operations U(ϕ), the detailed architecture of which is provided in Supplementary Note S6. E. (**c**) Classical neural network with 2 hidden layers and (**d**) corresponding two-qubit quantum circuit implementation, demonstrating data re-uploading strategy. (**e**) Two-qubit entangled quantum circuit, showcasing potential for enhanced quantum processing. |0⟩ and |1⟩ represent the initial qubit states, and the measurement symbol indicates the final readout. U(x) denotes data encoding, while U(ϕ) represents trainable quantum operations.

The methodology employed in this study utilizes data re-uploading, a technique that enables sophisticated integration of data input and processing within a unified quantum circuit. The circuit’s efficacy is enhanced through the optimization of rotational angles, which are governed by classical parameters. These parameters are refined iteratively by minimizing a specific cost function. This function quantifies the circuit’s proficiency in categorizing data points into distinct regions on the Bloch sphere, with each region corresponding to a unique class. The final stage of the process involves quantum measurement, wherein the overlap between the resultant quantum state and predefined label states is determined, facilitating classification decisions. The architecture and operational principles of this quantum classifier can be elucidated through comparisons with classical neural networks, as illustrated in Figure 14.

To encode classical data into the quantum circuit, we use the universal data re-uploading strategy [19]. This method encodes classical inputs using parameterized single-qubit rotations of the form U(θ+ω. x), where x is the input vector, ω is a learnable weight vector, and θ is a trainable bias. These values are embedded into alternating rotation gates—typically $R_{X}(x)$ and $R_{Z}(x)$—so that each input influences multiple non-commuting axes, enhancing the circuit’s expressivity. For more details, please visit Supplementary Note S6. E, “Variational Circuit Architecture and Parameterization”.

This re-uploading mechanism allows classical data to be injected multiple times throughout the circuit, analogous to how data flows through multiple neurons in a classical neural network. Each layer in our quantum circuit can be viewed as a composite of a data-encoding gate and a trainable unitary operation, similar in spirit to layers in a deep neural network.

This approach avoids costly multi-qubit data preparations and is compatible with current NISQ devices, as it uses only single-qubit operations for data encoding. It has been theoretically shown that such circuits can approximate arbitrary functions, similar to the universal approximation theorem in classical machine learning. Consequently, this encoding strategy supports universal function representation with a shallow quantum circuit.

Figure 14a depicts a rudimentary two-input classical neural network, which finds its quantum counterpart in the single-qubit circuit shown in Figure 14b. This quantum circuit employs alternating data upload operations U(x) and trainable unitary operations U(ϕ). The classical network’s single hidden layer corresponds to a quantum classifier utilizing a solitary qubit. The neurons in the classical hidden layer are analogous to the processing units or “layers” in the quantum classifier, denoted as “A”, “B”, through “N” in Figure 14. In this quantum circuit, |0⟩ represents the initial qubit state, U(x) denotes data encoding, U(ϕ) represents trainable quantum operations, and the measurement symbols indicate the final readout process. The combination of U(x) and U(ϕ) constitutes a single layer in the quantum framework.

More intricate classical networks featuring additional layers correspond to multi-qubit quantum circuits implementing data re-uploading strategies. Advanced quantum circuits can incorporate entanglement between qubits, demonstrating the potential for enhanced quantum processing capabilities that surpass classical limitations.

Figure 14c illustrates a two-input classical neural network with two hidden layers, which is equivalent to a two-qubit quantum classifier shown in Figure 14d. The number of hidden layers in the classical neural network corresponds to the number of qubits in the quantum classifier. Similarly, the number of neurons in the classical hidden layers indicates the number of processing units or layers in the quantum classifier, represented as “A”, ”B”, through “N”, for 2-qubit as well as “A′ “, ”B′ “, through “N′” for 2-qubit entangled. In this quantum circuit, |0⟩ and |1⟩ represent the initial qubit states.

Figure 14e depicts a two-qubit entangled classifier. This configuration is similar to the two-qubit classifier but incorporates a CZ gate after each processing unit or layer to induce entanglement between the two qubits, thereby enhancing the quantum circuit’s computational capabilities. Supplementary Note S6 presents a thorough and detailed exploration of the process of reuploading, including how it occurs and is handled within the quantum classifier framework. It also delves into the application of various cost functions, explaining their role in optimizing the classifier’s performance. Additionally, the note outlines the universality of the single-qubit classifier and transition from using a single-qubit classifier to a two-qubit classifier, discussing the steps involved, the challenges faced, and the improvements in performance that arise from the inclusion of an additional qubit. This discussion provides valuable insights into the evolution and scaling of quantum classifiers. For LCP and non-LCP approaches, and cost functions for the quantum classifier, please refer to Supplementary Note S6. All random datasets were generated using a standardized and reproducible procedure across experiments. Specifically, we used fixed sampling distributions, predefined input dimensionality, and enforced class balance. For reproducibility, we applied a fixed random seed (30) for all fixed datasets, while allowing stochasticity in randomized runs by omitting the seed. A concise summary of the dataset generation methodology, circuit design, parameterization, and optimization techniques is presented in Table 1, providing a structured overview of the key features discussed throughout this section.

Optimizing a single-qubit classifier involves minimizing a function across a complex parameter space. This paper evaluates four distinct minimization techniques: L-BFGS-B, COBYLA, Nelder–Mead, and Sequential Least Squares Programming (SLSQP). L-BFGS-B, a quasi-Newton method, efficiently handles large-scale problems with linear memory usage. COBYLA, designed for constrained optimization, does not require derivative calculations. Nelder–Mead, a direct search method, is effective for problems lacking derivative information. SLSQP minimizes functions while adhering to specific constraints, using a quadratic approximation of the objective function. Each method has unique strengths and limitations, with its effectiveness varying based on the specific classification task and dataset characteristics. The choice of optimization method significantly impacts the classifier’s performance, especially when dealing with smaller training sets and the inherent challenges of local minima in quantum circuits. For completeness and reproducibility, formal mathematical formulations and algorithmic descriptions of the four optimization methods used in this study—L-BFGS-B, COBYLA, Nelder–Mead, and SLSQP—are provided in Supplementary Note S7 (Subsections A–D). This section includes the rules, constraint-handling strategies, and the key characteristics of each optimizer to support a deeper understanding of their role in quantum circuit training.

In addition, Supplementary Note S8 contains the primary Python (version 3.13.1; Python Software Foundation, Wilmington, DE, USA) implementation, accompanied by a comparative analysis with the related work presented in the study [19,55].

## Figures and Tables

**Table 1:** Summary of the main methodological components used in this study, including dataset properties, quantum circuit architecture, parameter structure, and optimization routines.

Category	Aspect	Detail
Dataset Generation	Sampling and Dimensionality	Uniform sampling in ${\left[-1,\;1\right]}^{2}$ using scaled random values.
	Class Balance	Circle (balanced via r=2/π); Line (split by x1=x2).
	Reproducibility	A fixed random seed (30) was used for fixed datasets, whereas it was omitted for randomized runs.
	Task Types	Two classification tasks: line (LCP) and circle (non-LCP).
Model Architecture	Circuit Design	Layered re-uploading structure with optional qubit entanglement.
	Gate Types	U(ϕ) single-qubit gates; CZ gates for inter-qubit entanglement.
	Parameterization	θ and α tensors shape; randomly initialized.
	Circuit Variants	1Q: 3 × layers. 2Q: 6 × layers. 2Q + ent: +CZ.
	Data Re-uploading	Encoding uses $\theta_{encoded}=\theta +\alpha ⊗x$ per input.
	Optimization Methods	The optimization methods L-BFGS-B, COBYLA, Nelder–Mead, and SLSQP were used to minimize the fidelity- and trace-distance-based loss functions.

## Data Availability

All data generated or analyzed during this study are included in this published article and its Supplementary Information Files.

## Supplementary Materials

The following supporting information can be downloaded at: https://www.mdpi.com/article/10.3390/e28050550/s1.
