# Supplementary material for: Strategic Data Re-Uploads: A Pathway to Improved Quantum Classification Data Re-Uploading Strategies for Improved Quantum Classifier Performance
Source: Entropy (Basel). 2026 May 13;28(5):550. doi: 10.3390/e28050550 (PMC13205981; doi:10.3390/e28050550)
Supplement: Supplementary file 1 [file entropy-28-00550-s001.zip › entropy-4186102-supplementary.pdf]

Strategic Data Re-Uploads: A Pathway to Improved Quantum Classification
Data Re-Uploading Strategies for Improved Quantum Classifier Performance

Supplementary Documentation

Authors: Sara Aminpour[1,2,3], Yaser M. Banad[1,3], Sarah S. Sharif[1,2,3]*

Author Affiliations:
[1] School of Electrical and Computer Engineering, University of Oklahoma,
Norman, OK 73019, USA
[2]Center for Quantum Research and Technology, University of Oklahoma,
Norman, OK 73019 USA
[3]Intelligent Neuromorphic and Quantum Understanding for Innovative Research and Engineering (INQUIRE) Laboratory, Norman, OK 73019, USA

*Corresponding author: Sarah Sharif (email: s.sh@ou.edu)

Table of contents

- Supplementary Note S1. Range of training samples and number of layers
- Supplementary Note S2. Evaluating LCP and non-LCP approaches for fidelity cost function in fixed and random datasets for 1-qubit classifier for four different minimization methods
- Supplementary Note S3: Evaluating LCP and non-LCP approaches for trace distance cost function in fixed and random datasets for 1-qubit classifier
- Supplementary Note S4: performance comparison of 5-layer single-qubit quantum classifiers using fidelity and trace distance cost functions across various classification tasks and dataset types
- Supplementary Note S5: Evaluating LCP and non-LCP approaches for fidelity in fixed and random datasets for 2-qubit and 2-qubit entangled classifiers
- Supplementary Note S6: Method
- Supplementary Note S7: Optimization Methods
- Supplementary Note S8: Comparing our developed code with original reference

Supplementary Note S1: Range of training samples and number of layers

Figure S1.1 illustrates the performance of a quantum classifier utilizing a fidelity cost function within a five-layer framework for circular pattern classification in a fixed dataset, employing the L-BFGS-B optimization method. The analysis encompasses training data up to 250 samples to benchmark our algorithm against the findings from the reference [1]. The diagram depicts training accuracy with a blue dashed line and test accuracy with a solid blue line, underscoring the algorithm's efficacy. A red dot highlights a notable benchmark from the reference, showing an 89% accuracy with 200 training samples, demonstrating parity with this published result. The inset provides a visual representation of the classification process. Notably, test accuracy begins at approximately 70%, rising impressively to 96% for a slightly expanded dataset of 210 samples. Remarkably, with as few as 60 training samples, the model achieves a test accuracy of 91.8%, and the discrepancy between training and test accuracy diminishes with the inclusion of 90 samples. This

observation underscores the efficiency of our approach, highlighting its capability to reach high accuracy levels without necessitating extensive training data.

Figure S1.2 showcases a systematic evaluation of a circular pattern classification model across a spectrum of architectural depths, ranging from 1 to 5 layers. The graphical analysis reveals that models with a solitary layer lag in performance compared to those with increased layer counts, marking a clear trend: as the number of layers escalates, so does the model's classification accuracy. Specifically, a single-layer setup achieves a peak accuracy of 61.9%, whereas a more complex five-layer configuration significantly elevates this metric to 88.8%, even when limited to only 35 training samples. This observation underscores a critical insight—enhancing the model's depth systematically improves its predictive capabilities, a phenomenon consistent with the advantages afforded by the data reuploading strategy integral to our approach. Given this marked improvement in model efficacy with layer augmentation, the paper prioritizes an in-depth investigation and discourse on the five-layer model's architecture, focusing on its ability to optimize classification accuracy with efficient utilization of training data.

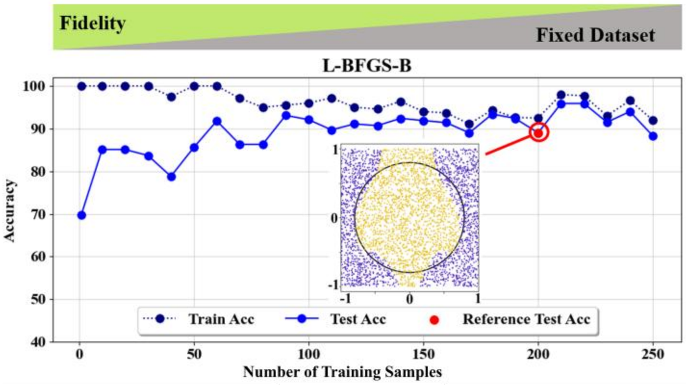

Figure S1.1 Train and test accuracy of fidelity for the 5-layer model of circle classification and fixed dataset for L-BFGS-B minimization method. The inset graph shows the visualization of a nonlinear classification reported on[1].

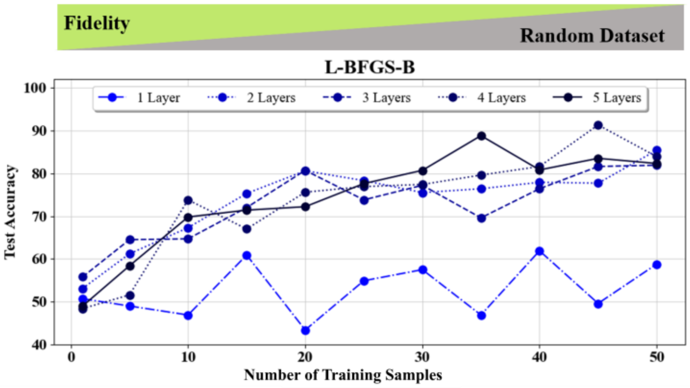

Figure S1.2. Evaluate the test accuracy of fidelity for circle classification and random dataset for L-BFGS-B minimization method, ranging from 1 to 5 layers.

Supplementary Note S2: Evaluating non-linear and linear classification approaches for fidelity cost function in fixed and random datasets for 1-qubit classifier for four different minimization methods

Figure 2 illustrates a comparison of four distinct optimization techniques, namely L-BFGS-B, COBYLA, Nelder-Mead, and SLSQP, applied to the task of classifying the circle pattern. The comparison evaluates both training and test accuracies using a fixed dataset of 4000 test samples and 5 layers. Initially, all algorithms demonstrate a perfect training accuracy of 100% with just a single sample, a result that aligns with expectations. However, as we increase the sample size, a divergence in performance becomes evident for these four minimization methods. The L- BFGS-B method maintains a training accuracy close to 90%, showcasing its robustness against overfitting. In contrast, COBYLA, Nelder-Mead, and SLSQP show significant variability and a decline in training accuracy, indicating a susceptibility to overfitting. Interestingly, the peak accuracy for COBYLA, Nelder-Mead, and SLSQP is achieved with merely 50 samples, beyond which overfitting becomes a significant issue. This observation suggests that, unlike L-BFGS-B, which requires a minimum of 100 samples to achieve an accuracy of 92%, the other three methods can attain over 95% accuracy with only 50 samples. L-BFGS-B does not reach this high accuracy level at 100 samples, and its performance slightly declines with an increase in training samples after 150 training samples. This analysis highlights the critical importance of carefully selecting the number of training samples based on the minimization method used. The right choice can

effectively prevent overfitting, thereby enhancing classification accuracy. This insight is crucial for optimizing machine learning models and ensuring their generalizability and efficiency in practical applications.

Figure 3 delves into the accuracy of these four distinct minimization methods —L-BFGS-B, COBYLA, Nelder-Mead, and SLSQP— when applied to a fidelity cost function and a random dataset for circle classification. This analysis underscores a consistent trend across all methods: an initial increase in test accuracy corresponding to the rise in the number of training samples, yet fails to surpass a peak accuracy of 90%. This trend highlights the inherent challenges faced by these minimization methods when dealing with random datasets. In the L-BFGS-B method as depicted in figure 3(a), showcases a notable performance, achieving its highest test accuracy of 88.8% with 35 training samples. This point also marks the narrowest gap of 5% between training and test accuracy, indicating a relatively high level of model efficiency and generalization at this sample size. However, as the analysis progresses, it becomes apparent that increasing the number of training samples beyond this optimal point does not translate to improved performance. The gap between the train and test accuracy remains notably constant at around 10% even as the sample size is increased to 70 training samples. Transitioning to the COBYLA method, as depicted in figure 3(b), a different performance pattern emerges. Contrary to L-BFGS-B, COBYLA achieves its best test accuracy at 84.8% with a higher training sample equal to 70. This method experiences fluctuations, yet it is noteworthy that the gap between training and test accuracies exhibits a decreasing trend, suggesting a gradual improvement in model generalization compared to the initial stability seen with L-BFGS-B. Figure 3(c) focuses on the Nelder-Mead method, highlighting a decrease in the gap between training and test accuracies as the number of training samples increases, culminating in a maximum accuracy of 86.9% with 60 training samples. Figure 3(d) examines the SLSQP method, which shows an increase in test accuracy up to 50 training samples before demonstrating a decline in both training and test accuracies. This shows the SLSQP method is more prone to overfitting. The SLSQP method reaches a maximum accuracy of 86.7% when applied to a dataset of 50 samples. These results, as detailed in figure 6, provide vital insights into the performance of various minimization methods when working with a fidelity cost function and a random dataset. The diverse outcomes emphasize the importance of choosing an optimal number of training samples to prevent overfitting and enhance accuracy. This underlines the delicate balance needed to fully leverage these computational methods in practical scenarios.

Figure 4 illustrates a comparison of four different optimization techniques applied to the task of classifying line patterns, using fidelity-based cost function and the fixed dataset. The subplot (a) focuses on the performance of the L-BFGS-B method. Here, the training accuracy starts at a perfect 100% and impressively remains above 97% even as the number of training samples increases. Conversely, the test accuracy initiates at a relatively lower rate of 62.2% with just a single sample yet it progressively improves, reaching approximately 95% accuracy with 75 training samples and slightly declines for larger training samples. An initial notable gap between the training and test accuracy is evident, but this gap diminishes significantly as the dataset expands with more training data, indicating an improvement in the model's ability to generalize from the training to the unseen test data. The subplot (b) depicts the results obtained using the COBYLA algorithm, which exhibits a performance pattern similar to that of the L-BFGS-B method, consistently achieving 100% accuracy on the training data. The accuracy on the test set starts at 66.9% and steadily improves as more training samples are added, ultimately reaching 95% when 125 samples are used for training. The disparity between training and test set accuracies mirrors the pattern observed with the L-BFGS-B method, consistently manifesting across all training dataset sizes. The Nelder-Mead approach, shown in figure 4(c), achieves a notable test accuracy of 97.7% with 125 training samples. The inset provides a graphical visualization of line classification using this minimization method at this specific point, illustrating that the line classification performance is exceptionally well. The visualization clearly demonstrates the method's effectiveness in accurately separating the data points into distinct classes, highlighting the Nelder-Mead method's precision and robustness in handling line classification tasks with a substantial number of training samples. Furthermore, the training and test accuracy curves show a notably smaller gap, converging to the same value with training sets of 100 and 125 samples. The final subplot (d) evaluates the performance of the SLSQP method, which closely aligns with the results from the COBYLA method. The test set accuracy exhibits a progressive increase, rising from 62.7% to 96.6%. The disparity between the training and test accuracies is similar to that observed with the COBYLA method. In

summary, all four optimization techniques demonstrate a reduction in overfitting as the training dataset size increases, ultimately achieving a test accuracy of at least 95% when training with 125 samples for this line classification task.

Figure 5 showcases an analysis of the classification accuracy obtained using the same minimization methods across random datasets. Consistently, a rise in the number of training samples correlates with an increase in test accuracy across all methods evaluated. Notably, with just 50 training samples, all methods surpass the 90% accuracy threshold. Specifically, in figure 5(a), the L-BFGS-B method reaches the peak accuracy of 92.8% with 50 training samples. It was observed that as the number of samples increased, the disparity between train and test accuracies for the L-BFGS-B method began to narrow, although this gap persisted in being slightly wider than that observed in the other methods. Figure 5(b) demonstrates that the COBYLA method, with the same number of samples, attains a superior accuracy of 93.5%. This suggests that COBYLA not only reaches high classification accuracy with a minimal dataset but also demonstrates better generalization compared to L-BFGS-B, as reflected by the narrower gap between its training and test accuracies. Figure 5(c) examines the Nelder-Mead method, showing its peak accuracy of 93% with 40 training samples, after which its accuracy slightly declines. Interestingly, the smallest disparity between training and test accuracies—only 1.8%—occurs in 50 training samples. Despite slightly lower accuracy at this point, this smallest gap signifies that the Nelder-Mead method achieves a remarkable balance between learning from the training data and generalizing to unseen data, highlighting its efficiency and potential for precise model tuning. Figure 5(d) illustrates that the SLSQP method achieves an impressive peak test accuracy of 96.4% for line classification using a random dataset, attained with 45 training samples. At this juncture, the discrepancy between training and test accuracies is notably small, indicating a high level of model precision and generalization. Like the Nelder-Mead method, the SLSQP method exhibits a nonmonotonic increment in test accuracy as a function of training samples, as indicated by the irregular slope of test accuracy. This fluctuation suggests that for these methods, adding more training samples does not straightforwardly translate to higher test accuracies, highlighting the complexity of optimizing model performance across different minimization techniques.

A comparison of figures 2 and 4 reveals that the accuracy curves for line classification are more stable and consistent across all optimization techniques when compared to those for circle classification. The accuracy values for classifying circle patterns display greater variability and fluctuations than those observed in the line classification task. The observed differences in performance between circle and line classification could stem from several technical factors: (1) Line classification likely represents a more straightforward pattern that aligns better with the linear decision boundaries most classifiers are adept at identifying. In contrast, circle classification involves recognizing more complex, non-LCP, which can challenge the classifiers' ability to generalize from the training data without overfitting or underfitting. (2) The algorithms applied for circle classification might be more prone to getting trapped in local minima due to the more intricate decision boundaries required to accurately classify circular patterns. This can hinder the optimization process, leading to increased fluctuations in classification accuracy as the model struggles to find the global optimum. (3) The differences in performance may also reflect the inherent adaptability of the algorithms to the specific types of classification tasks with the geometric properties. A comparative analysis of Figures 6 and 8 indicates that the specific characteristics of the classification problem significantly affect the potential to attain higher accuracy with fewer samples. The fluctuations in the line classification pattern are less pronounced than those in the circle classification pattern. This observation underscores the importance of selecting appropriate optimization methods based on the complexity of the classification problem.

Supplementary Note S3: Evaluating non-linear and linear classification approaches for trace distance cost function in fixed and random datasets for 1-qubit classifier

Figure 6 showcases the effectiveness of the trace distance cost function in classifying circular patterns within a fixed dataset. In subplot (a), the L-BFGS-B minimization method achieves its highest test accuracy at 79.2% with a dataset comprising 100 training samples. Subplot (b) examines the performance of the COBYLA method, which displays greater variability in training accuracy than L-BFGS-B but ultimately achieves a higher peak test accuracy of 84.6%, also with 100 training samples. Notably, COBYLA demonstrates enhanced generalization capabilities relative to other methods, as indicated by the narrower margin between its training and testing accuracies. This performance suggests that, when applied alongside the trace distance cost function, the COBYLA method is particularly adept at optimizing parameters for improved generalization to unseen testing data. An accompanying visualization within the inset illustrates the

classification of circular patterns at this accuracy peak. In subplot (c), the analysis shifts to the performance of the Nelder-Mead method, which records its optimal test accuracy at 72.6% utilizing 60 training samples. This method exhibits signs of overfitting, a condition where the model learns the training data too closely and fails to generalize well to new, unseen data. Despite a narrowing gap between training and testing accuracies as the number of training samples grows, a concurrent decline in training accuracy is observed, which adversely affects the overall test accuracy. This pattern suggests a limitation in the Nelder-Mead method's capacity to effectively handle the trace distance cost function, likely due to its inherent characteristics such as reliance on simplex-based optimization, which might struggle with the complexity of the trace distance landscape. Consequently, this method appears less suited for tasks requiring robust generalization from the trace distance cost function, particularly in scenarios demanding accurate classification of complex patterns with a limited dataset. In subplot (d), the focus turns to the SLSQP method which attains its peak test accuracy at 83.6% with a dataset of 100 training samples. The disparity between training and testing accuracy contracts by increasing the training samples, indicating an improvement in the model's ability to generalize from the training to the testing dataset. However, even at the point of 100 training samples, the gap between training and testing accuracies, while reduced, remains significant. This persistent gap suggests that while the SLSQP method is effective at learning and generalizing from the given data, there is still a margin for optimization to further bridge the difference in accuracies. Each optimization technique successfully minimizes the cost function and attains perfect accuracy on the training set using a comparatively small number of samples. However, their performance varies considerably when it comes to generalizing to the test set. This highlights the crucial role played by the choice of optimization algorithm in determining the overall effectiveness of the model. In conclusion, when considering the fixed dataset and the trace distance cost function, the COBYLA method demonstrates superior performance in optimizing the parameters to generalize effectively to unseen test data. Compared to the other techniques evaluated, it necessitates fewer training samples to achieve satisfactory accuracy on the test set.
Figure 7 illustrates how the accuracy on both the training and test sets evolves as the number of training samples grows, specifically for the task of classifying circular patterns using the trace distance cost function, evaluated on a randomly generated dataset. Similar to all scenarios analyzed so far, a common pattern emerges where test accuracy begins at a relatively low level for all minimization methods but demonstrates a consistent increase as more training data is provided. This trend highlights the methods' capacity to effectively learn distinguishing features, thereby enhancing their ability to generalize to unseen data. Specifically, in subplot (a), the L-BFGS-B method illustrates impressive learning efficiency, with test accuracy exceeding 70% after incorporating just 40 training samples and achieving its highest test accuracy of 77.8% with 45 training samples. In subplot (b), the COBYLA method's performance is slightly lower compared to L-BFGS-B, plateauing at a test accuracy of 72.8% with 45 training samples. This performance indicates that while COBYLA may be susceptible to some degree of overfitting, it nonetheless achieves a reasonable level of generalization. Subplot (c) explores the Nelder-Mead method, which reaches its peak test accuracy of 75.1% with 50 training samples. Subplot (d) utilizes the SLSQP method, which shows fluctuations in its training accuracy remaining above 80%. The test accuracy for SLSQP was enhanced significantly, reaching 74.6% with 50 samples. This fluctuation and eventual rise in test accuracy underscores the method's potential for optimizing classification tasks, despite the initial variability. In sum, the L-BFGS-B method stands out for achieving the highest test accuracy among the methods evaluated, requiring only 45 training samples to reach this optimum on a random dataset. Summarily, employing the trace distance cost function across these various minimization strategies yields test accuracy ranging from 65% to 78% on the random dataset, illustrating the function's effectiveness and the distinct performance capabilities of each minimization method.

Figure 8 offers a comparative analysis of the accuracy achieved by four different optimization methods when applied to a trace distance cost function for line pattern classification using a fixed dataset. Subplot (a) highlights the L-BFGS-B method, showcasing its high level of stability in training accuracy. The test accuracy shows a steady increase, reaching 91.8% with 100 training samples. While there is a substantial gap between the accuracies of the training and test sets at the outset, this difference gradually narrows as more training samples are introduced. This highlights the L-BFGS-B method's capacity to adapt and learn more complex patterns effectively, demonstrating robustness and in leveraging larger datasets for improved generalization. The subplot (b) illustrates the results obtained using the COBYLA method.

In contrast to the L-BFGS-B approach, the accuracy of the training set shows greater fluctuations, even experiencing a drop to 56.9% at one instance before rebounding. The test accuracy follows a similar pattern to that seen in L-BFGS-B, beginning at 49.8% and increasing to 87.4%. Once the training set size reaches 80 samples, both the training and test accuracies seem to reach a plateau, slightly below the 90% mark. In subplot (c), the Nelder-Mead method starts with a modest test accuracy of 55.3%, which significantly improves to 87% with the addition of 60 training samples demonstrating a similar trend as the L-BFGS-B method. Initially, a pronounced gap exists between training and test accuracies, which persists until the dataset is expanded to include 80 training samples. Beyond this point, the sign of overfitting emerges, as demonstrated by a decline in training accuracy while test accuracy plateaus. For 100 training samples, the test accuracy interestingly becomes 2% higher than the training accuracy, indicating a unique inversion where the model performs slightly better on unseen data than on the training set itself, a rare occurrence that may suggest the model has reached a point of optimization where it generalizes exceptionally well to new data. The subplot (d) of figure 11 presents the results of the SLSQP method. Notably, this technique achieves the highest accuracy on the test set, reaching 93.3% using just 40 training examples. The SLSQP method appears to be the most appropriate choice for trace distance classification tasks, as it exhibits a smaller discrepancy between its performance on the training and test datasets. The inset provides a visual representation of the SLSQP's performance at this specific point. To summarize, all optimization methods demonstrate an upward trajectory in test accuracy as the size of the training dataset increases, suggesting enhanced generalization capabilities of the model. Among the four techniques evaluated, the SLSQP method seems to strike the most favorable balance between its performance on the training and test sets.

Figure 9 presents a comparison of different optimization techniques when applied to the task of classifying line pattern using a randomly generated dataset and a cost function based on trace distance. In subplot (a), we examine the performance of the L-BFGS-B method, which attains its peak test accuracy of 86.3% with 55 training samples. Before reaching this point, the method's test accuracy demonstrated considerable variability, oscillating between 70% and 80% as the number of training samples ranged from 20 to 50. However, a notable improvement occurs when the dataset is expanded to 55 training samples, at which the test accuracy leaps to 86.3%, effectively surpassing the earlier fluctuation band. This pivotal moment also marks the occurrence of the smallest gap between training and test accuracies, showcasing a significant enhancement in the model's ability to generalize from the training dataset to unseen data, thereby achieving an optimal balance at this specific training sample size. Subplot (b) delves into the efficacy of the COBYLA optimization method, which achieves its highest test accuracy of 86.8% with a relatively smaller dataset of 35 training samples. Beyond this optimal threshold, signs of overfitting become apparent, as both training and test accuracies start to decline. This pattern suggests that while the COBYLA method is highly effective up to a certain point, adding more training samples beyond this number paradoxically hampers the model's performance. The decline in accuracy indicates that the model begins to memorize the training data rather than learning to generalize, leading to a decrease in its ability to accurately predict outcomes on unseen data. This observation underscores the importance of identifying the ideal number of training samples to maximize the effectiveness of the COBYLA method without crossing into the territory of overfitting. In subplot (c), the focus is on the Nelder-Mead optimization method, which shows some fluctuations in performance before reaching its maximum test accuracy. It successfully achieves a test accuracy of 88.1% with 40 training samples. However, akin to the pattern observed with the COBYLA method, the Nelder-Mead method also sees a decline in both training and test accuracies when additional training samples are added beyond this optimal number. This decline serves as a clear indication of the onset of overfitting, suggesting that while the Nelder-Mead method can efficiently utilize a certain number of training samples to improve its predictive accuracy, exceeding this number leads to a reduction in model performance. In subplot (d), a more continuous and stable increase in test accuracy is observed with each increase in the number of training samples. This trend results in the highest test accuracy being recorded at 88.3% with 55 training samples. Unlike the previous methods discussed, this subplot suggests a method that maintains its efficiency and ability to generalize well without showing immediate signs of overfitting up to this point. The gradual and consistent improvement in test accuracy highlights the method's effective learning curve and suggests an optimal balance between learning from the training data and applying this knowledge to unseen data.

Supplementary Note S4: performance comparison of 5-Layer single-qubit quantum classifiers using fidelity and trace distance cost functions across various classification tasks and dataset types

Figure S4.1 offers a comparative analysis of the highest accuracies achieved for two distinct classification patterns – linear (line) and non-linear (circle) – across the four distinct minimization methods when applied to both random and fixed datasets within the context of a fidelity cost function. The analysis reveals a notable trend: in circle classification tasks, the fixed dataset consistently yields higher accuracies than their random counterparts for all tested minimization methods. This suggests that the inherent geometric complexities of non-LCP may align more closely with the simpler structure of fixed datasets, thereby facilitating more accurate classification. Similarly, for line classification, the fixed dataset leads to enhanced accuracies with the L-BFGS-B and SLSQP methods, indicating these methods' effectiveness in leveraging structured data to accurately discern linear relationships. However, the random dataset achieves better accuracy when classified using the Nelder-Mead method. This could suggest that the Nelder-Mead method, known for its simplicity and direct search approach, might be particularly adept at navigating the stochastic nature of random datasets to identify linear patterns. Across all algorithms, the task of classifying non-LCP, especially within random datasets, emerges as inherently challenging. This complexity likely stems from the algorithms' varying abilities to parse and learn from the unpredictable variance found in random datasets, as well as the added difficulty of accurately modeling non-linear relationships. The findings underscore the critical importance of selecting the appropriate minimization method based on the dataset's nature and the classification task's geometric complexity to optimize classification accuracy.

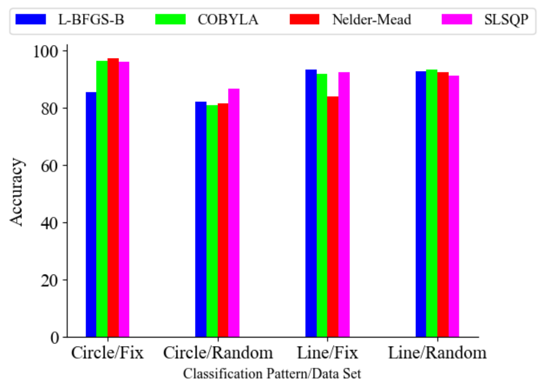

Figure S4.1. Evaluating of Fidelity cost function test accuracy of 5-layer model across 50 samples for LCP and non-LCP problems for random and fixed datasets in four minimization methods.

Figure S4.2 provides the performance comparison of two distinct classification patterns—line and circle—across four different minimization methods when applied to both random and fixed datasets, this time employing the trace distance cost function. A pivotal observation emerges when comparing the performance of circle classification with a fixed dataset (circle/fixed) against the fidelity cost function results presented in figure S4.1. It is evident that the accuracies achieved using the trace distance cost function are notably lower across all minimization methods compared to those obtained with the fidelity cost function. This discrepancy highlights the inherent challenges and differences in how each cost function interacts with the underlying data and the classification task at hand. The trace distance cost function, known for quantifying the distinguishability between quantum states, may present a more complex landscape for optimization, particularly when applied to classical data patterns such as lines and circles. This complexity could lead to lower classification accuracy as the minimization methods struggle to navigate the nuances of the trace distance landscape effectively. Such an observation underscores the importance of cost function selection in machine learning tasks, emphasizing that the

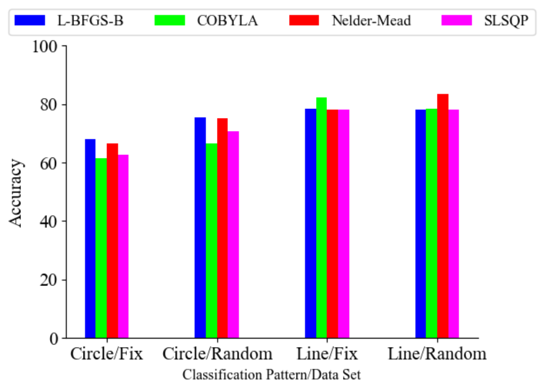

Figure S4.2. Evaluating of trace distance test accuracy of 5-layer model across 50 samples for LCP and non-LCP problems for random and fixed datasets in four minimization methods.

choice of cost function can significantly impact the model's ability to learn and generalize from the data. The comparative analysis in figure S4.2 serves as a testament to the nuanced interplay between cost functions, dataset types (fixed vs. random), and the geometric nature of the classification patterns, offering valuable insights into optimizing classification accuracy through strategic method and cost function selection.

In addition, the fixed dataset achieves superior accuracy specifically when employing the COBYLA minimization method, indicating a unique synergy between COBYLA's optimization strategy and the structured nature of fixed datasets for LCP. Conversely, for the random dataset, there's a notable trend where it consistently outperforms the fixed dataset across all other minimization methods, suggesting that the stochastic characteristics of random datasets may be better suited to the optimization landscapes these methods navigate, particularly for LCP. In circle classification tasks, the random dataset not only demonstrates improved accuracy over the fixed dataset for all minimization methods but also reinforces the observation that random datasets generally offer a more favorable context for the trace distance cost function across both classification patterns. This enhancement in accuracy with random datasets could be attributed to the trace distance cost function's sensitivity to the variances within the dataset, allowing for more effective differentiation and classification of non-LCP like circles when the data is less predictable.

Supplementary Note S5: Evaluating non-linear and linear classification approaches for fidelity in fixed and random datasets for 2-qubit and 2-qubit entangled classifiers

Focusing on figure 10(a), we observe the performance of a single-qubit system applied to a LCP pattern. The system demonstrates a steep initial learning curve, with accuracy rapidly increasing from 51.6% to 92% after just 75 training samples. This sharp rise highlights the single-qubit system's ability to efficiently learn and generalize from a relatively small dataset. The notable jump in accuracy suggests that a properly trained single-qubit classifier can capture the essential features of the LCP task with high precision. After reaching 92% accuracy at 75 training samples, the system stabilizes, maintaining a test accuracy consistently in the range of 92% to 97.7% as the training sample size increases to 125. The minimal fluctuation in accuracy indicates a robust performance, with the single-qubit system effectively avoiding overfitting even as the training data expands. The stable test accuracy underscores the system's reliability and suitability for LCP tasks where computational simplicity and consistent performance are crucial. In terms of computational cost, as shown in figure 1(d), the single-qubit system exhibits a gentle increase in computational time, reaching 62.15 seconds for 250 training samples. This computational efficiency, coupled with the system's stable accuracy, makes the single-qubit classifier an appealing option for linear problems, particularly in scenarios where computational resources are limited but high accuracy is still required.

In figure 10(b), the performance of the 2-qubit classifier in a LCP task shows a more gradual improvement in accuracy compared to the single-qubit system. The initial accuracy is relatively high, starting at 73.2% with just one training sample, which suggests that the additional qubit provides a more robust representation of the problem space even with minimal training. As the number of training samples increases to 75, the accuracy rises steadily, reaching 94.1%. This gradual improvement, as opposed to the sharp jump seen in the single-qubit system, highlights the ability of the 2-qubit classifier to build on its already strong initial performance with increasing training data. Beyond 50 training samples, the 2-qubit classifier continues to demonstrate incremental gains, eventually peaking at around 95.7% test accuracy with 175 training samples. Notably, the test accuracy fluctuates between 92% and 96% throughout this range, suggesting that while the system performs consistently well, there are slight variations in how the test data is classified as more training samples are introduced. These fluctuations could indicate that the system is sensitive to the nature of the training data or potentially approaching the limits of its capacity for linear classification. From a computational perspective, shown in Figure 1(e), the 2-qubit classifier exhibits a significant increase in computational time as the number of training samples grows. By the time the training sample size reaches 250, the computational time extends to around 260 seconds. This is a sharp contrast to the single-qubit system, illustrating the tradeoff between the enhanced accuracy and robustness offered by the 2-qubit classifier and the increased computational demands. For LCP tasks, this suggests that while the 2-qubit classifier provides higher initial accuracy and steady performance improvements, it comes at the cost of a much higher computational burden, making it potentially less suitable for scenarios where time or resources are constrained.

Examining figure 10(c), the performance of the 2-qubit entangled classifier in a LCP task reveals a distinctive pattern when compared to non-entangled systems. The initial accuracy is relatively low, starting at 51.3% with just one training sample. This suggests that the entanglement introduces complexities that make the system less effective in identifying patterns from very limited data. However, as the number of training samples increases to 75, the system exhibits a steep improvement in accuracy, reaching 93.3%. This rapid climb indicates that while the entangled system may struggle with very small datasets, it quickly capitalizes on additional training samples to enhance its classification performance. As the training samples continue to increase beyond 75, the 2-qubit entangled classifier shows notable fluctuations in accuracy, ranging between 88% and 97.5%. These fluctuations, which are more pronounced than those seen in the single-qubit or non-entangled 2-qubit classifier, suggest that entanglement introduces both benefits and challenges. On one hand, the system achieves the highest peak accuracy (97.5%) among all three systems, demonstrating its potential for superior performance. On the other hand, the variability in test accuracy highlights the sensitivity of the entangled system to the training data, possibly indicating overfitting or instability when processing larger datasets. In terms of computational cost, as shown in figure 10(f), the 2-qubit entangled classifier mirrors the trend seen in the non-entangled 2-qubit classifier, with computational time increasing significantly as the number of training samples rises. At 250 training samples, the computational time reaches 260 seconds, similar to the non-entangled classifier. Despite this computational burden, the 2-qubit entangled classifier offers a potential advantage in terms of peak accuracy, making it a compelling choice for applications where achieving the highest possible accuracy is paramount, even if it comes with the tradeoff of greater computational complexity and variability in performance.

In comparing the classifier, we observe clear tradeoffs between simplicity, stability, and computational complexity. The single-qubit classifier is the most stable and computationally efficient but may not reach the same peak accuracies as the more complex systems. The 2-qubit classifier offers higher initial accuracy and consistent improvement but requires significantly more computational resources. Finally, the 2-qubit entangled system, while achieving the highest peak accuracy, also introduces greater instability and computational demands, making it best suited for scenarios where peak performance is the priority, and computational cost is less of a concern. Ultimately, the choice of system depends on the specific requirements of the classification task, such as whether stability, computational efficiency, or peak accuracy is the primary objective.

Figure 11 presents a comprehensive analysis of two quantum classifiers - a 2-qubit classifier and a 2-qubit entangled classifier for non-LCP. The results are displayed across six subplots, labeled (a) through (f), which provide insights into the performance and characteristics of these classifiers under various conditions. Subplots (a) and (b) show the train and test accuracies as a function of the number of training samples for the 2-qubit and the 2-qubit entangled classifiers, respectively. In both cases, we observe that the accuracies generally improve as the number of training samples increases. However, the 2-qubit classifier (a) shows higher initial test accuracy, 73.5%, and a more stable performance across different sample sizes. The 2-qubit entangled classifier (b) starts with lower test accuracy, 47.6% but shows significant improvement as the sample size increases. Both classifiers seem to converge in terms of train and test accuracy around 175 training samples, which explains why this number was chosen for subsequent analyses. Subplots (c) and (d) illustrate how the number of layers in the quantum circuit affects the accuracies of the classifiers for a specific number of training samples. For the 2-qubit classifier (c), we see a general upward trend in both train and test accuracies as the number of layers increases, with some fluctuations. The 2-qubit entangled classifier (d) shows a more pronounced improvement with increasing layers, especially in the early stages. Both classifiers appear to reach a plateau in performance after about 12-15 layers, suggesting that further increases in circuit depth may not yield significant improvements. Subplots (e) and (f) depict the computational time required as the number of layers increases for the 2-qubit and the 2-qubit entangled classifiers, respectively. Both show a clear exponential growth in computational time as the number of layers increases. This trend is consistent across both classifiers, indicating that the computational cost scales similarly regardless of whether entanglement is used. Comparing the classifiers overall, we can see that the 2-qubit classifier generally achieves higher accuracies with fewer training samples and maintains more consistent performance across different numbers of layers. The 2-qubit entangled classifier, while starting with lower accuracy, shows more dramatic improvements as both the number of training samples and layers increase. This

suggests that entanglement might provide additional expressive power to the classifier, allowing it to capture more complex patterns in the data as the circuit depth increases. However, this potential advantage comes at the cost of increased sensitivity to the number of training samples and layers, as evidenced by the more volatile accuracy curves in subplots (b) and (d). The computational time plots (e) and (f) remind us that increasing the number of layers quickly becomes computationally expensive for both classifiers, which is an important consideration in practical applications. In conclusion, these results provide valuable insights into the trade-offs between accuracy, circuit complexity, and computational cost for quantum classifiers, highlighting the potential benefits and challenges of using entanglement in quantum machine learning tasks.

Figure 12 presents a comparative analysis of four optimization algorithms (COBYLA, L-BFGS-B, NELDER MEAD, and SLSQP) applied to a LCP using a quantum circuit with 2 qubits. The experiment uses a random dataset with 250 training samples and employs a fidelity cost function to measure the performance. The figure includes subplots depicting accuracy and computational time for both 2-qubit and 2-qubit entangled classifiers. In terms of accuracy, both training and test accuracies are generally high across all algorithms. However, there are subtle differences between the algorithms. As shown in figure 12(a), for the 2-qubit entangled classifier, the average test accuracy is approximately 2% higher than the 2-qubit non-entangled classifier. In terms of individual performance, the L-BFGS-B minimization method consistently achieves the highest test accuracy, reaching 96.3% for non-entangled and 97% for entangled classifiers. The overall variation in test accuracy between the highest and lowest performing algorithms is 2.3%. For 2-qubit non-entangled classifier, COBYLA exhibits the lowest test accuracy at 94%, while for 2-qubit entangled classifier, NELDER MEAD achieves the lowest test accuracy of 95.3%. Computational time analysis reveals interesting patterns across both classifiers. In figure 12(c) the 2-qubit classifier, computational time varies widely from 9 to 90 minutes. COBYLA stands out as the fastest method, completing the task in just 9 minutes, while L-BFGS-B and NELDER_MEAD are the most time-consuming at 90 and 89 minutes respectively. SLSQP occupies a middle ground, requiring 45 minutes. In figure 12(d) the 2-qubit entangled classifier generally shows improved computational efficiency. While COBYLA maintains its swift performance at 9 minutes, other methods see reduced execution times. Most notably, L-BFGS-B improves from 90 to 71 minutes, a significant reduction, while NELDER_MEAD and SLSQP methods remain at 87 and 44 minutes respectively. In conclusion, this analysis reveals that the 2-qubit entangled classifier generally outperforms the 2-qubit non-entangled classifier in both accuracy and computational efficiency. The L-BFGS-B method consistently provides the highest accuracy, albeit at a higher computational cost. COBYLA emerges as a well-balanced option, offering good accuracy with minimal computational time, particularly in the 2-qubit entangled classifier. These findings underscore the significant impact of minimization method selection on both accuracy and computational time in quantum machine learning tasks. Furthermore, the 2-qubit entangled classifier's closer alignment of train and test accuracies suggests enhanced generalization capabilities, a crucial factor in practical machine learning applications.

Figure 13 shows a comprehensive comparison of different optimization methods for non-LCP using both 2-qubit and 2-qubit entangled classifiers for a specific random dataset. This analysis encompasses four optimization techniques: COBYLA, L-BFGS-B, NELDER_MEAD, and SLSQP, evaluating their performance based on accuracy and computational time for 250 number of training samples. In the accuracy graphs (a) and (b), we observe distinct performance patterns between the 2-qubit and 2-qubit entangled classifiers. For the 2-qubit classifier, L-BFGS-B demonstrates the highest accuracy, with both train and test accuracies exceeding 90%. COBYLA shows the lowest performance, with a test accuracy of 76.7% and train accuracy 81.4%. NELDER_MEAD and SLSQP exhibit intermediate performance, with test accuracies in the 82-87% range. The 2-qubit entangled classifier, depicted in graph (b), shows overall improved accuracy across all methods. L-BFGS-B maintains its superior performance, while COBYLA shows significant improvement, reaching accuracies to 85.4%. Notably, the gap between train and test accuracies is generally smaller in the 2-qubit entangled classifier, suggesting better generalization. The computational time graphs (c) and (d) reveal interesting efficiency patterns. In the 2-qubit classifier, COBYLA is the fastest method, requiring only 9 minutes. L-BFGS-B, despite its high accuracy, is the most time-consuming at 130 minutes. NELDER_MEAD takes 89 minutes, while SLSQP requires 45 minutes. The 2-qubit entangled classifier (graph d)

shows generally reduced computational times. COBYLA remains the fastest, maintaining its 9-minute runtime. L-BFGS-B shows the most dramatic improvement, reducing its time to 81 minutes. Interestingly, NELDER_MEAD in the 2-qubit entangled classifier takes slightly longer than L-BFGS-B, at 88 minutes. SLSQP maintains a consistent performance of about 42 minutes in both systems. These results highlight the trade-offs between accuracy and computational efficiency in quantum machine learning tasks. The 2-qubit entangled classifier demonstrates superior performance in both accuracy and computational time across all methods. L-BFGS-B consistently provides the highest accuracy but at a higher computational cost, especially in the 2-qubit classifier. COBYLA emerges as a balanced option, offering good accuracy with minimal computational time, particularly in the entangled system. This analysis underscores the importance of choosing appropriate optimization methods and leveraging entanglement to enhance the performance of quantum classification tasks.

Supplementary Note S6: Method

Quantum computing manipulates quantum systems to enhance information processing, leveraging superposition to simultaneously operate on multiple states for faster and more complex computation. At its core is the qubit, represented in a two-dimensional Hilbert space, with operations governed by quantum gates. These gates, essential for altering quantum states, must be unitary to ensure the conservation of probability, a fundamental principle of quantum dynamics[2].

The framework of a quantum circuit unfolds in three key phases: encoding classical data into quantum format, manipulating the quantum state using quantum gates, and measuring the quantum state post-transformation. This process transitions from preparing an initial quantum state, through strategic alterations via quantum gates affecting computation outcomes, to a final probabilistic measurement—distinguishing quantum computing's potential and challenges from deterministic classical computing.

Achieving optimal performance in quantum computing requires a nuanced understanding of these phases, including the initial state preparation, the strategic selection and application of quantum gates, and the final measurement process. Each component must be meticulously optimized to perform specific tasks, such as classification, highlighting the intricate interplay between quantum mechanics and computational logic in the design and execution of quantum algorithms.

A. RE-UPLOADING CLASSICAL INFORMATION AND PROCESSING

The integration of classical information into quantum computing represents a groundbreaking approach to data processing and analysis. This process begins with the strategic encoding of data into the initial wave function’s coefficients within a quantum circuit[3]. In simpler terms, data is initially uploaded through the manipulation of qubits via rotational operations on a computational basis. This foundational step sets the stage for executing sophisticated quantum algorithms, including those designed for classification tasks.

The most successful programming paradigm in machine learning is predicated on artificial neural networks, which represent a highly abstracted and simplified model inspired by the human brain [4]. An artificial neural network comprises interconnected units or nodes known as artificial neurons, often arranged in layers [5]. These networks are characterized by their diverse architectures and the ability to learn from data through the adjustment of a vast network of parameters during the training phase. Among the various types of neural networks, feed-forward neural networks exemplify the process of sequential data processing, where input data is transformed layer by layer, simulating a form of data re-uploading at each neuron. This mechanism of data re-uploading and processing in ANNs provides a parallel to the innovative approach of constructing a universal quantum classifier using a single qubit. The essence of this quantum computing strategy lies in the repeated introduction of classical data at different stages of computation, analogous to the data processing in a single hidden layer neural network. This process can be visualized diagrammatically, as shown in figure 14 in the main paper. the neural network architecture is depicted, where data points are fed into individual processing units, analogous to neurons within the hidden layer. These neurons collectively process these input data, culminating in the activation of a final neuron responsible for constructing the output for subsequent analysis. Similarly, in the quantum domain, the single-qubit classifier incorporates data points into each stage of the computation through unitary rotations. These rotations are not isolated; rather, each one builds upon the transformations applied by its predecessors, effectively integrating the input data multiple times throughout the computation. The culmination of this process is a quantum state that encapsulates the computational outcome.

To construct a universal quantum classifier with only a single qubit, a complex integration of data input and computational processing within a single quantum circuit is crucial. We achieve this objective through the deployment of parametrized quantum circuits (PQCs). In these circuits, certain rotational angles are meticulously adjusted based on

classical parameters, which are refined through an optimization process aimed at minimizing a specifically defined cost function.

The cost function plays a pivotal role in the operational efficacy of the quantum classifier. It quantitatively assesses the circuit's performance in segregating data points into distinct categories, which are represented as separate regions on the Bloch sphere. Each of these regions corresponds to a different class, and the classifier's goal is to assign data points to the correct class based on their features.

B. Dataset Generation Methodology

In this section, we provide a detailed and standardized description of how both fixed and random datasets were constructed and evaluated throughout the study.

- **Sampling Distribution and Dimensionality:**

All data points were sampled independently and uniformly from the interval $[-1,1]^2$, corresponding to the two-dimensional input space used in all classification problems. The sampling was performed using np.random.rand(2) and scaled via the transformation $x \mapsto 2x - 1$ to ensure full coverage of the $[-1,1]$ range along both axes.

- **Class Balance and Geometric Design:**

We carefully selected geometric parameters to maintain balanced class distributions. In the circle classification task (non-LCP), we used a radius of $r = \sqrt{(2/\pi)}$ such that the area inside and outside the circle is equal, yielding a 50/50 class distribution. For the linear classification task (LCP), we defined the decision boundary as x1=x2, which symmetrically divides the domain $[-1,1]^2$ and likewise ensures class balance by design.

- **Reproducibility and Standardization:**

To ensure consistency across experiments, we fixed the random seed at 30 for all fixed dataset runs. The training set sizes varied from 1 to 200 samples depending on model complexity, while each test set consisted of 4000 uniformly sampled points. For randomized datasets, we deliberately omitted the use of a fixed seed, ensuring that each of the 20 iterations generated a new sample set from the same distribution. This approach allowed us to test the classifier's generalization ability and robustness under different data realizations. Accuracy and runtime were averaged across these 20 independent runs to obtain statistically meaningful results.

- **Dataset Types and Parameters:**

We focused on two primary classification tasks: (1) a line, representing linear separability (LCP), and (2) a circle, representing a basic non-linear separability case (non-LCP). These were chosen as fundamental and interpretable decision boundaries to evaluate the baseline performance of the quantum classifiers. All geometric parameters, such as the radius for non-LCP and the slope/intercept for LCP, were held fixed across all trials to ensure consistency and enable fair comparison across circuit designs and optimization methods.

C. Applying Cost Functions

In the realm of quantum computing, a quantum circuit is distinguished by its processing angles θ_i and associated weights w_i, leading to the generation of a final state $|\psi>$. The measurement outcomes from this state are used to compute a classification error metric, defined as χ^2. The goal is to minimize this error metric by adjusting the circuit's classical parameters, a process that can be effectively managed through various supervised machine learning techniques.

At the heart of using quantum measurement for classification tasks lies the approach of optimally aligning observed outputs with specific target classes. This alignment is primarily facilitated by the principle of maximizing orthogonality between the output states, ensuring clear distinction[6]. In the context of binary (dichotomous) classification, this means categorizing each observation into one of two predefined classes—referred to here as class A and class B. The decision criterion involves comparing the probabilities of observing the quantum state $P(0)$ for outcome 0 and $P(1)$ for outcome 1. If $P(0) > P(1)$, the observation is assigned to class A; otherwise, it falls under class B. To enhance this binary classification scheme, one can introduce a bias (λ), adjusting the threshold for classification such that observation is deemed part of class A if $P(0)$ is greater than λ, and class B if it falls below. The value of λ is chosen to maximize classification accuracy on a training dataset. The effectiveness of this approach is then confirmed through evaluation on a separate validation dataset.

Viewed through a geometric lens, the single-qubit classifier operates within a 2-dimensional Hilbert space —the Bloch sphere—where data encoding and classification decisions are delineated through specific rotational parameters. Any operation $L(i)$ is a rotation on the Bloch sphere surface. From this viewpoint, any point can be classified using just one unitary operation. Consequently, we can transfer any point to another point on the Bloch sphere by precisely selecting the rotation angles. However, when dealing with multiple data points, a single rotation may not suffice due to differing optimal rotation requirements. The solution lies in introducing additional layers into the quantum circuit, enabling distinct

rotation and fostering a richer feature map. Within this enhanced feature space, data points can be effectively segregated into their respective classes based on their positioning within the Bloch sphere's regions, thereby enabling a sophisticated and adaptable approach to quantum classification.

1) FIDELITY COST FUNCTION

The goal is to align the quantum states ($|\psi(\vec{\theta},\vec{w},\vec{x})>$) as closely as possible to a designated target state on the Bloch sphere, as outlined in [1]. This alignment can be quantitatively assessed by measuring the angular distance between the labeled state and the data state, using the metric of relative fidelity [7]. The primary objective focuses on maximizing the average fidelity between the quantum states produced by the circuit and the label states corresponding to their respective classes. To facilitate this, a cost function is introduced and mathematically formulated as Equation S1:

$$\chi_f^2(\vec{\theta},\vec{\omega}) = \sum_{\mu-1}^{M} \left(1 - \left|\left\langle \tilde{\psi}_s | \psi\left(\vec{\theta},\vec{\omega},\vec{x}_\mu\right)\right\rangle\right|^2\right) \qquad \text{(S1)}$$

where $|\tilde{\psi}_s\rangle$ is the correct label state of the μ data point, which will correspond to one of the classes.

2) TRACE DISTANCE COST FUNCTION

In quantum information theory, quantifying the dissimilarity between two quantum states is a fundamental problem. Various distance measures have been proposed, each with its unique properties and applications. One such measure is the trace distance, which captures the distinguishability between two quantum states [7]. Perez-Salinas et al. have analyzed the fidelity cost function with data re-uploading [1]. However, the authors do not consider the case of the trace distance cost function, which is what we focus on in this section. We will explore the definition and properties of the trace distance, particularly in the context of single-qubit systems, and discuss its potential as a cost function for quantum classifiers.

Despite the different mathematical formulations of trace distance and fidelity, these two measures share many similar properties and are widely used in the quantum computing and quantum information community. However, depending on the specific application, one measure may be more convenient or easier to work with than the other. This versatility and widespread adoption of both trace distance and fidelity in the field motivates our decision to discuss and compare these two important distance measures in the context of quantum classifiers. The trace distance between quantum states ρ and σ can be defined as,

$$D(\rho,\sigma) \equiv \frac{1}{2} tr\left|\rho - \sigma\right|^2 \qquad \text{(S2)}$$

The trace distance between two single-qubit states, represented by their respective Bloch vectors $\vec{r}$ and $\vec{s}$, is equal to one-half of the Euclidean distance between these vectors on the Bloch sphere. [7]

$$D(\rho,\sigma) = \frac{\left|\vec{r} - \vec{s}\right|}{2}. \qquad \text{(S3)}$$

This relation provides a geometric interpretation of the trace distance for single-qubit systems, linking it to the intuitive notion of distance in three-dimensional space.

D. From Universality of the Single-Qubit Classifier to the Expansion into Multi-Qubit Quantum Classification

A key challenge in Quantum Machine Learning (QML) involves creating quantum circuits that efficiently handle complex tasks like classification without excessive use of quantum resources. The Universal Approximation Theorem (UAT) [8] is crucial for tackling this issue, demonstrating that a single-layer neural network with an appropriate activation function can approximate any continuous function to a desired accuracy, assuming enough hidden neurons are available. This UAT finds a compelling parallel in the quantum computing domain, particularly when considering the dynamics of quantum circuits. Here, the classical activation function is analogously performed by a unitary rotation acting upon a qubit. Specifically, a single-qubit quantum classifier, enhanced by the technique of data re-uploading, emerges as a universal approximator for any conceivable classification function. This universality hinges on the frequency of data re-uploading throughout the circuit's span [1], underscoring that even a solitary qubit is capable of encoding and processing multifaceted high-dimensional data. This is achieved through the execution of multiple rotations, each characterized by distinct angles and weights. The culmination of these processes is a final quantum state, which is then analyzed against a predefined target state correlating to each class. Optimization of the circuit's parameters is pursued through the minimization of a cost function, which is indicative of the fidelity or trace distance between the comparative states.

By establishing the UAT within the context of quantum classifiers, a robust theoretical foundation is laid, alongside practical guidelines for the design and implementation of quantum circuits adept at sophisticated and non-LCP tasks with minimal quantum resource expenditure. This breakthrough not only forges a theoretical link between quantum circuits and neural networks but also paves the way for innovative approaches in QML. Through this lens, quantum circuits are envisioned not merely as computational tools but as entities with the potential to parallel, and possibly surpass, the capabilities of their classical neural network counterparts, inspiring a new wave of methodologies in the realm of QML.

To enhance the performance of the single-qubit classifier, it is proposed to extend it to a multi-qubit system. Adding more qubits, especially with entanglement, can improve the classifier's effectiveness, similar to how adding layers enhances neural networks. Entanglement may provide a quantum advantage in classification, though the analogy between multi-qubit classifiers and neural networks with entanglement is not fully understood and requires further exploration. Perez et al. propose a measurement strategy for multi-qubit classifiers, which extends the single-qubit approach. These strategies utilize a fidelity-based cost function.

E. Variational Circuit Architecture and Parameterization

To fully specify the architecture of the quantum classifier and support reproducibility, we detail here the structure of the variational circuits used in this study. The models are built using a data re-uploading framework, in which classical input data is embedded into the quantum circuit by modifying gate parameters via a linear transformation. Each circuit is composed of multiple layers; each layer includes data-dependent single-qubit gates followed by optional entanglement gates between qubits.

The primary quantum gates used are $U(\phi)$ gates, which are universal single-qubit rotation gates parameterized by three angles $\phi = (\theta, \varphi, \lambda)$. These gates are used for both trainable processing and data encoding. When entanglement is introduced, Controlled-Z (CZ) gates are applied between qubit pairs.

The parameter set for each circuit is divided into two categories:

- θ, the base rotation angles, organized as a tensor of shape (qubits, layers, 3),
- α, the data encoding weights, shaped as (qubits, layers, data dimension).

The total number of trainable parameters scales with both the number of qubits and the number of re-uploading layers. For example, the single-qubit configuration contains 3×layers trainable parameters. The two-qubit configuration without entanglement uses two parallel $U(\phi)$ gates per layer (one on each qubit), resulting in 6×layers parameters. When entanglement is included, the same number of $U(\phi)$ gates are used, along with (layers−1) Controlled-Z gates placed between adjacent qubit layers.

The data encoding follows the transformation $\theta_{encoded} = \theta + \alpha \otimes x$, where x is the input feature vector. This allows the same circuit structure to dynamically adapt to different input data points while preserving trainable components.

Class label encoding differs based on the cost function used. For fidelity-based classification, labels are represented as computational basis states such as $|0\rangle$ or $|1\rangle$. For trace-distance-based classification, target class states are defined using Bloch sphere coordinates.

Supplementary Note S7: Optimization Methods

In practice, deploying a parameterized quantum classifier involves a process of minimizing within the parameter space that delineates the circuit's configuration. The process is often termed a hybrid algorithm, denoting the symbiotic relationship and advantages derived from combining quantum logic and classical logic. In particular, the ensemble of angles (θ_i) and weights (w_i) defines a parameter space that requires systematic exploration to achieve the minimization of χ^2.

The occurrence of local minima is unavoidable e. The arrangement of rotation gates results in an intricate multiplication of independent trigonometric functions, suggesting that our problem is characterized by a widespread distribution of minima.

The primary challenge boils down to minimizing a function that is defined by a vast array of parameters. In the case of a single-qubit classifier, the total number of parameters can be expressed as, where represents the problem's dimension (that is, the dimension of), and signifies the number of layers. Among these parameters, three are rotational angles, while the rest pertain to the weight [1]. To identify the most effective solution, we evaluate the performance of four distinct minimization techniques: the L-BFGS-B method, the COBYLA method, the Nelder-Mead method, and the Sequential Least Squares Programming (SLSQP) method.

The key challenge in optimizing a single-qubit classifier involves minimizing a function across a complex parameter space, calculated as $(3 + d)N$, where "d" is the problem's dimension and "N" is the number of layers. Also, in addition,

we need to consider rotational angles and the weight ($\vec{w}_i$) corresponding to the dimension [1]. To discover the optimal solution, we delve into the efficiency of four diverse minimization strategies: the L-BFGS-B, COBYLA, Nelder-Mead, and Sequential Least Squares Programming (SLSQP) methods.

A. L-BFGS-B METHOD

The L-BFGS-B technique, part of the quasi-Newton optimization methods, refines the Broyden–Fletcher–Goldfarb–Shanno (BFGS) approach by efficiently using limited computer memory [10]. Its design excels in handling optimization tasks involving numerous variables, offering a linear memory usage advantage, making it highly effective for large-scale problems [11].

The L-BFGS-B method is widely recognized as a cornerstone technique across various advanced applications in the field of graphics [12,13]. It specializes in minimizing a scalar function of one or several variables by initiating with a preliminary estimate of the optimum value. Through iterative refinement, it progressively improves upon this initial estimate to approach an optimal solution. The method employs function derivatives to determine the direction of steepest descent and approximates the Hessian matrix (second-order derivatives) using limited memory. The parameter update rule is given by[14]:

$$\theta_{k+1} = \theta_k - \alpha_k H_k^{-1} \nabla f(\theta_k)$$

where θ_k is the current parameter vector, $\nabla f(\theta_k)$ is the gradient, H_k^{-1} is an approximation of the inverse Hessian, and α_k is a step size typically determined by line search. This method is particularly efficient in handling large-scale problems due to its low memory usage and fast matrix-vector multiplications.

B. CONSTRAINED OPTIMIZATION BY LINEAR APPROXIMATION METHOD

COBYLA (Constrained Optimization BY Linear Approximation) is an optimization algorithm designed to minimize a scalar objective function that depends on one or more variables, subject to constraints [15,16]. One of the key features of COBYLA is that it does not require the calculation of derivatives, such as gradients or Hessians, of the objective function or constraints. This makes COBYLA particularly useful in situations where the derivatives are unknown, unreliable, or computationally expensive to obtain [15]. Instead of requiring gradients or Hessians, COBYLA constructs linear approximations of both the objective function and constraints within a trust region framework. At each iteration, it solves a subproblem defined by: $\min_{\theta} f(\theta)$ subject to $c_i(\theta) \geq 0$ and approximates the objective function locally as:

$$f(\theta + \Delta\theta) \approx f(\theta) + \nabla f(\theta)^T \Delta\theta$$

although $\nabla f(\theta)$ is never explicitly calculated—its effect is estimated using linear interpolation.

COBYLA has been effectively utilized in quantum computing, especially as a classical optimization routine within Variational Hybrid Quantum-Classical Algorithms (VHQCAs) [17]. These algorithms employ a parameterized quantum circuit, or ansatz, which is refined through a dynamic interchange between a classical computer and a quantum device. The classical computer adjusts the ansatz's parameters to minimize a cost function, which the quantum device efficiently evaluates. Through iterative updates based on the cost function outcomes, the VHQCA aims to discover the most effective ansatz configuration for specific problems. The derivative-free characteristic of COBYLA makes it particularly advantageous for this setting, where the cost functions often lack easily computable or analytically defined derivatives.

C. NELDER-MEAD METHOD

The Nelder-Mead algorithm, introduced by John Nelder and Roger Mead in 1965, is a widely used direct search method for unconstrained optimization problems [18]. The algorithm operates by maintaining a simplex of n+1 points in an n-dimensional space, iteratively moving the simplex toward the optimal solution through a series of transformations, including reflection, expansion, contraction, and shrinkage [18]. These operations are defined as follows:

- **Reflection:**

$$\theta_r = \bar{\theta} + \alpha(\bar{\theta} - \theta_h)$$

- **Expansion:**

$$\theta_e = \bar{\theta} + \gamma(\theta_r - \bar{\theta})$$

- **Contraction:**

$$\theta_c = \bar{\theta} + \rho(\theta_r - \bar{\theta})$$

- **Shrinkage:**

$$\theta_i = \theta_l + \sigma(\theta_i - \theta_l)$$

Here, $\bar{\theta}$ is the centroid of the best n points, θ_h is the worst-performing point, and $\alpha, \gamma, \rho,$ and σ are user-defined coefficients controlling the behavior of each transformation. This method is especially effective in low-dimensional, non-

convex optimization landscapes and is widely used when the objective function is noisy, non-differentiable, or discontinuous.

Recent studies have focused on enhancing the Nelder-Mead algorithm to improve its efficiency and adaptability. Gao and Han [19]proposed an implementation of the Nelder-Mead algorithm with adaptive parameters, which can automatically adjust the parameter values based on the optimization progress. This adaptive approach has been shown to improve the algorithm's convergence speed and solution quality [19].

Its capacity to address problems in which derivative information is not readily accessible renders it a favorable option for numerous applications in QML. However, it is essential to conduct comprehensive evaluations to scrutinize the method's accuracy, efficiency, and sensitivity to the initial guess for each unique application [20,21].

D. SEQUENTIAL LEAST SQUARES PROGRAMMING METHOD

The Sequential Least Squares Programming (SLSQP) method is an optimization technique that minimizes functions while adhering to specific constraints [22]. It is based on Sequential Quadratic Programming (SQP), which simplifies the optimization problem into a series of smaller, more manageable quadratic subproblems. In each subproblem, a quadratic approximation of the objective function and constraints is constructed using a second-order parabolic curve to model the function's behavior near a specific point. SLSQP updates this approximation using the quasi-Newton method. Specifically, the subproblem it solves takes the form:

$$\min_{\Delta\theta} \left[1/2\ \Delta\theta^T B_k \Delta\theta + \nabla f(\theta_k)^T \Delta\theta \right]$$

subject to:

$$c_i(\theta_k) + \nabla c_i(\theta_k)^T \Delta\theta \geq 0 \text{ (inequality constraints)}$$

$$h_j(\theta_k) + \nabla h_j(\theta_k)^T \Delta\theta = 0 \text{ (equality constraints)}$$

where B_k is an approximation to the Hessian of the Lagrangian, and $\nabla f, \nabla c_i$, and ∇h_j are gradients of the objective and constraint functions.

Additionally, SLSQP applies a least-squares method to solve these quadratic subproblems, striving to minimize the total squared deviations between the approximation and actual function values. This method can handle both equality and inequality constraints, including variable bounds, by integrating a penalty function that imposes additional costs for any constraint or bound violations. SLSQP ensures efficient convergence by terminating the optimization process upon meeting a predefined convergence criterion, typically related to changes in the objective function value or the gradient vector's norm. This safeguard prevents indefinite computations, ensuring timely solutions.

Local minima are common challenges in both neural networks and quantum classifiers due to their complex mathematical structures—neural networks with compounded nonlinear functions and quantum circuits with prevalent trigonometric functions. This complexity increases the likelihood of encountering local minima during optimization. Moreover, with smaller training sets, the choice of optimization method is crucial. For instance, the Nelder-Mead method is noted for its robustness, particularly its lower susceptibility to local minima.

It is also critical to recognize that minimization methods are sensitive to noise, which can significantly impact their effectiveness, especially in practical quantum computing applications [17].

Data Availability

All data generated or analyzed during this study are included in this published article and its supplementary information files.

References

1 Pérez-Salinas, A., Cervera-Lierta, A., Gil-Fuster, E. & Latorre, J. I. Data re-uploading for a universal quantum classifier. *Quantum* **4**, 226 (2020).

2 Schuld, M., Sinayskiy, I. & Petruccione, F. An introduction to quantum machine learning. *Contemporary Physics* **56**, 172-185 (2015).

3 Schuld, M., Bocharov, A., Svore, K. M. & Wiebe, N. Circuit-centric quantum classifiers. *Physical Review A* **101**, 032308 (2020).

4 LeCun, Y., Bengio, Y. & Hinton, G. Deep learning. *nature* **521**, 436-444 (2015).

5 Li, W. & Deng, D.-L. Recent advances for quantum classifiers. *Science China Physics, Mechanics & Astronomy* **65**, 220301 (2022).

6 Helstrom, C. W. Quantum detection and estimation theory. *Journal of Statistical Physics* **1**, 231-252 (1969).

7 Nielsen, M. A. & Chuang, I. L. *Quantum computation and quantum information*. (Cambridge university press, 2010).

8 Hornik, K. Approximation capabilities of multilayer feedforward networks. *Neural networks* **4**, 251-257 (1991).

9 Cerezo, M., Verdon, G., Huang, H.-Y., Cincio, L. & Coles, P. J. Challenges and opportunities in quantum machine learning. *Nature Computational Science* **2**, 567-576 (2022).
10 Liu, D. C. & Nocedal, J. On the limited memory BFGS method for large scale optimization. *Mathematical programming* **45**, 503-528 (1989).
11 Zhu, C., Byrd, R. H., Lu, P. & Nocedal, J. Algorithm 778: L-BFGS-B: Fortran subroutines for large-scale bound-constrained optimization. *ACM Transactions on mathematical software (TOMS)* **23**, 550-560 (1997).
12 Liu, Y. *et al.* On centroidal Voronoi tessellation—energy smoothness and fast computation. *ACM Transactions on Graphics (ToG)* **28**, 1-17 (2009).
13 Wang, L., Zhou, K., Yu, Y. & Guo, B. Vector solid textures. *ACM Transactions on Graphics (TOG)* **29**, 1-8 (2010).
14 Byrd, R. H., Lu, P., Nocedal, J. & Zhu, C. A limited memory algorithm for bound constrained optimization. *SIAM Journal on scientific computing* **16**, 1190-1208 (1995).
15 Virtanen, P. *et al.* SciPy 1.0: fundamental algorithms for scientific computing in Python. *Nature methods* **17**, 261-272 (2020).
16 Bonet-Monroig, X. *et al.* Performance comparison of optimization methods on variational quantum algorithms. *Physical Review A* **107**, 032407 (2023).
17 Pellow-Jarman, A., Sinayskiy, I., Pillay, A. & Petruccione, F. A comparison of various classical optimizers for a variational quantum linear solver. *Quantum Information Processing* **20**, 202 (2021).
18 Nelder, J. A. & Mead, R. A simplex method for function minimization. *The computer journal* **7**, 308-313 (1965).
19 Gao, F. & Han, L. Implementing the Nelder-Mead simplex algorithm with adaptive parameters. *Computational Optimization and Applications* **51**, 259-277 (2012).
20 Abel, S., Blance, A. & Spannowsky, M. Quantum optimization of complex systems with a quantum annealer. *Physical Review A* **106**, 042607 (2022).
21 Lockwood, O. An empirical review of optimization techniques for quantum variational circuits. *arXiv preprint arXiv:2202.01389* (2022).
22 Kraft, D. A software package for sequential quadratic programming. *Forschungsbericht- Deutsche Forschungs- und Versuchsanstalt fur Luft- und Raumfahrt* (1988).

Supplementary Note S 8: Comparing the developed code for this research with original reference.

Left: developed code

```
# coding=utf-8
#######################################################################
#Quantum classifier
#Sara Aminpour, Mike Banad, Sarah Sharif
#September 25th 2024

#School of Electrical and Computer Engineering/ Center for Quantum and Technology, University of Oklahoma, Norman, OK 73019 USA,
#######################################################################
#IMPORTANT_NOTE:
#The code on the left was developed by Sara Aminpour, while the code on the right serves as the reference implementation by Adrián Pérez-Salinas.
#The code on the left has been restructured to handle random data. So some certain sections has been deleted from the reference code.
#Additionally, our code on the left developed to analyze trace distance cost function and linear classification problem
#as well as necessary modification to apply COBYLA, L-BFGS-B, NELDER-MEAD, and SLSQP minimization methods.
#######################################################################
# so that the usage is automatized
import datetime
from data_gen import data_generator
from problem_gen import problem_generator, representatives, representatives_tr
from fidelity_minimization import fidelity_minimization
from trace_minimization import trace_minimization
from weighted_fidelity_minimization import weighted_fidelity_minimization
from test_data import Accuracy_test, tester
from save_data import write_summary, read_summary, name_folder, samples_paint, samples_paint_worldmap, laea_x, laea_y

from save_data import write_epochs_file, write_epoch, close_epochs_file, create_folder, write_epochs_error_rate

import numpy as np
import matplotlib.pyplot as plt
from circuitery import code_coords, circuit
from matplotlib.cm import get_cmap
from matplotlib.colors import Normalize

def minimizer(chi, problem, qubits, entanglement, layers, method, name,
              epochs=3000, batch_size=20,  eta=0.1):

        """
        This function creates data and minimizes whichever problem (from the selected ones)
        INPUT:
            -chi: cost function, to choose between 'fidelity_chi' or 'weighted_fidelity_chi'
            -problem: name of the problem, to choose among
                ['circle', '3 circles', 'hypersphere', 'tricrown', 'non convex', 'crown', 'sphere', 'squares', 'wavy lines']
            -qubits: number of qubits, must be an integer
            -entanglement: whether there is entanglement or not in the Ansätze, just 'y'/'n'
            -layers: number of layers, must be an integer. If layers == 1, entanglement is not taken in account

            -method: minimization method, to choose among ['SGD', another valid for function scipy.optimize.minimize]

            -name: a name we want for our our files to be save with
            -seed: seed of numpy.random, needed for replicating results
            -epochs: number of epochs for a 'SGD' method. If there is another method, this input has got no importance

            -batch_size: size of the batches for stochastic gradient descent, only for 'SGD' method
            -eta: learning rate, only for 'SGD' method
        OUTPUT:
            This function has got no outputs, but several files are saved in an appropiate folder. The files are

            -summary.txt: Saves useful information for the problem
            -theta.txt: saves the theta parameters as a flat array
            -alpha.txt: saves the alpha parameters as a flat array
            -weight.txt: saves the weights as a flat array if they exist
        """

        data, drawing = data_generator(problem)
        if problem == 'sphere':
            train_data = data[:500]
            test_data = data[500:]
        elif problem == 'hypersphere':
            train_data = data[:1000]
            test_data = data[1000:]
        else:
            train_data = data[:250]
            test_data = data[250:]

        if chi == 'fidelity_chi':
            Accuracy_tr=0
            Accuracy_te=0
            i=1
            while i<21:
                qubits_lab = qubits
                theta, alpha, reprs = problem_generator(problem,qubits, layers, chi,
                                                    qubits_lab=qubits_lab)

                theta, alpha, f = fidelity_minimization(theta, alpha, train_data, reprs,
                                                    entanglement, method,
                                                    batch_size, eta, epochs)

                acc_train = tester(theta, alpha, train_data, reprs, entanglement, chi)
                Accuracy_tr+=acc_train

                acc_test = tester(theta, alpha, test_data, reprs, entanglement, chi)
                Accuracy_te+=acc_test

                text_file_nn = open('acc.txt', mode='a+')
                text_file_nn.write(str(i) + problem +'_'+ chi +'_'+ str(qubits) +'Qubits_' + str(layers) +'Layers_' + entanglement +'_'+ method +'_'+'acc_train'+' = '+ str(acc_train))
                text_file_nn.write('\n')
                text_file_nn.write(str(i) + problem +'_'+ chi +'_'+ str(qubits) +'Qubits_' + str(layers) +'Layers_' + entanglement +'_'+ method +'_'+'acc_test'+' = '+ str(acc_test))
                text_file_nn.write('\n')
                text_file_nn.write('=====================================================================')
                text_file_nn.write('\n')
                text_file_nn.close()

                i+=1
            print(i-1)
            atr=Accuracy_tr/(i-1)
            ate=Accuracy_te/(i-1)


            text_file_nn = open('AverageAcc.txt', mode='a+')
            text_file_nn.write(problem +'_'+ chi +'_'+ str(qubits) +'Qubits_' + str(layers) +'Layers_' + entanglement +'_'+ method +'_'+'Ave_acc_train'+' = '+ str(atr))
            text_file_nn.write('\n')
            text_file_nn.write(problem +'_'+ chi +'_'+ str(qubits) +'Qubits_' + str(layers) +'Layers_' + entanglement +'_'+ method +'_'+ 'Ave_acc_test'+' = '+ str(ate))
            text_file_nn.write('\n')
            text_file_nn.write('=====================================================================')
            text_file_nn.write('\n')
            text_file_nn.close()

            write_summary(chi, problem, qubits, entanglement, layers, method, name,
                     theta, alpha, 0, f, atr, ate, epochs=epochs)
        elif chi == 'trace_chi':

            Accuracy_tr=0
            Accuracy_te=0
            i=1
            while i<21:
                qubits_lab = qubits
                theta, alpha, reprs = problem_generator(problem,qubits, layers, chi,
                                                    qubits_lab=qubits_lab)
                theta, alpha, f = trace_minimization(theta, alpha, train_data, reprs,
                                                    entanglement, method,
                                                    batch_size, eta, epochs)


                acc_train = tester(theta, alpha, train_data, reprs, entanglement, chi)
                Accuracy_tr+=acc_train

                acc_test = tester(theta, alpha, test_data, reprs, entanglement, chi)
                Accuracy_te+=acc_test

                text_file_nn = open('acc.txt', mode='a+')
                text_file_nn.write(str(i) + problem +'_'+ chi +'_'+ str(qubits) +'Qubits_' + str(layers) +'Layers_' + entanglement +'_'+ method +'_'+'acc_train'+' = '+ str(acc_train))
                text_file_nn.write('\n')
                text_file_nn.write(str(i) + problem +'_'+ chi +'_'+ str(qubits) +'Qubits_' + str(layers) +'Layers_' + entanglement +'_'+ method +'_'+'acc_test'+' = '+ str(acc_test))
                text_file_nn.write('\n')
                text_file_nn.write('=====================================================================')
                text_file_nn.write('\n')
                text_file_nn.close()


                i+=1
            print(i-1)
            atr=Accuracy_tr/(i-1)
            ate=Accuracy_te/(i-1)


            text_file_nn = open('AverageAcc.txt', mode='a+')
            text_file_nn.write(problem +'_'+ chi +'_'+ str(qubits) +'Qubits_' + str(layers) +'Layers_' + entanglement +'_'+ method +'_'+'Ave_acc_train'+' = '+ str(atr))
            text_file_nn.write('\n')
            text_file_nn.write(problem +'_'+ chi +'_'+ str(qubits) +'Qubits_' + str(layers) +'Layers_' + entanglement +'_'+ method +'_'+'Ave_acc_test'+' = '+ str(ate))
            text_file_nn.write('\n')
            text_file_nn.write('=====================================================================')
            text_file_nn.write('\n')
            text_file_nn.close()


            write_summary(chi, problem, qubits, entanglement, layers, method, name,
                    theta, alpha, 0, f, atr, ate, epochs=epochs)
        elif chi == 'weighted_fidelity_chi':


            Accuracy_tr=0
            Accuracy_te=0
            i=1
            while i<21:
                qubits_lab = 1
                theta, alpha, weight, reprs = problem_generator(problem,qubits, layers, chi,
                                                    qubits_lab=qubits_lab)
                theta, alpha, weight, f = weighted_fidelity_minimization(theta, alpha, weight, train_data, reprs,

                                                    entanglement, method)



                acc_train = tester(theta, alpha, train_data, reprs, entanglement, chi, weights=weight)
                Accuracy_tr+=acc_train

                acc_test = tester(theta, alpha, test_data, reprs, entanglement, chi, weights=weight)
                Accuracy_te+=acc_test

                text_file_nn = open('acc.txt', mode='a+')
                text_file_nn.write(str(i) + problem +'_'+ chi +'_'+ str(qubits) +'Qubits_' + str(layers) +'Layers_' + entanglement +'_'+ method +'_'+'acc_train'+' = '+ str(acc_train))
                text_file_nn.write('\n')
                text_file_nn.write(str(i) + problem +'_'+ chi +'_'+ str(qubits) +'Qubits_' + str(layers) +'Layers_' + entanglement +'_'+ method +'_'+'acc_test'+' = '+ str(acc_test))
                text_file_nn.write('\n')
                text_file_nn.write('=====================================================================')
                text_file_nn.write('\n')
                text_file_nn.close()


                i+=1
            print(i-1)
            atr=Accuracy_tr/(i-1)
            ate=Accuracy_te/(i-1)


            text_file_nn = open('AverageAcc.txt', mode='a+')
            text_file_nn.write(problem +'_'+ chi +'_'+ str(qubits) +'Qubits_' + str(layers) +'Layers_' + entanglement +'_'+ method +'_'+'Ave_acc_train'+' = '+ str(atr))
            text_file_nn.write('\n')
            text_file_nn.write(problem +'_'+ chi +'_'+ str(qubits) +'Qubits_' + str(layers) +'Layers_' + entanglement +'_'+ method +'_'+'Ave_acc_test'+' = '+ str(ate))
            text_file_nn.write('\n')
            text_file_nn.write('=====================================================================')
            text_file_nn.write('\n')
            text_file_nn.close()

            write_summary(chi, problem, qubits, entanglement, layers, method, name,
                    theta, alpha, weight, f, acc_train, acc_test, epochs=epochs)




def painter(chi, problem, qubits, entanglement, layers, method, name,
            standard_test = True, samples = 4000, bw = False, err = False):
    a=datetime.datetime.now()
    """
    This function takes written text files and paint the results of the problem
    INPUT:
        -chi: cost function, to choose between 'fidelity_chi' or 'weighted_fidelity_chi'
        -problem: name of the problem, to choose among
            ['circle', '3 circles', 'hypersphere', 'tricrown', 'non convex', 'crown', 'sphere', 'squares', 'wavy lines']

        -qubits: number of qubits, must be an integer
        -entanglement: whether there is entanglement or not in the Ansätze, just 'y'/'n'
        -layers: number of layers, must be an integer. If layers == 1, entanglement is not taken in account

        -method: minimization method, to choose among ['SGD', another valid for function scipy.optimize.minimize]

        -name: a name we want for our our files to be save with
        -seed: seed of numpy.random, needed for replicating results
        -standard_test: Whether we want to paint the set test used for checking when minimizing. If True, seed and samples are not taken in account
        -samples: number of samples of the test set
        -bw: painting in black and white
    OUTPUT:
        This function has got no outputs, but a file containing the representation of the test set is created

    """

    if chi == 'fidelity_chi':
        qubits_lab = qubits

    elif chi == 'trace_chi':
        qubits_lab = qubits

    elif chi == 'weighted_fidelity_chi':
        qubits_lab = 1

    if standard_test == True:
        data, drawing = data_generator(problem)
        if problem == 'sphere':
            test_data = data[500:]
        elif problem == 'hypersphere':
            test_data = data[1000:]
        else:
            test_data = data[250:]

    elif standard_test == False:
        test_data, drawing = data_generator(problem, samples = samples)

    if problem in ['circle','line', '2 lines', 'wavy circle','sphere', 'non convex', 'crown', 'hypersphere']:
        classes = 2
    if problem in ['tricrown']:
        classes = 3
    if problem in ['6squares']:
        classes = 6
    elif problem in ['3 circles','wavy lines','squares']:
        classes = 4

    #reprs = representatives(classes, qubits_lab)

    params = read_summary(chi, problem, qubits, entanglement, layers, method, name)

    if chi == 'fidelity_chi':
        reprs = representatives(classes, qubits_lab)
        theta, alpha = params
        sol_test, acc_test = Accuracy_test(theta, alpha, test_data, reprs, entanglement, chi)


    if chi == 'trace_chi':
        reprs = representatives_tr(classes, qubits_lab)
        theta, alpha = params
        sol_test, acc_test = Accuracy_test(theta, alpha, test_data, reprs, entanglement, chi)

    if chi == 'weighted_fidelity_chi':
        reprs = representatives(classes, qubits_lab)
        theta, alpha, weight = params
        sol_test, acc_test = Accuracy_test(theta, alpha, test_data, reprs,
                                    entanglement, chi, weights = weight)

    foldname = name_folder(chi, problem, qubits, entanglement, layers, method)
    samples_paint(problem, drawing, sol_test, foldname, name, bw)



    b=datetime.datetime.now()
    c=b-a
    text_file_nn = open('time.txt', mode='a+')
    text_file_nn.write(problem +'_'+ chi +'_'+ str(layers) +'Layers' +'_'+ 'painter' +' = '+ str(c))
    text_file_nn.write('\n')
    text_file_nn.close()
'''
def paint_world(chi, problem, qubits, entanglement, layers, method, name,
        seed = 30, standard_test = True, samples = 4000, bw = False, err = False):
    np.random.seed(seed)

    if chi == 'fidelity_chi':
        qubits_lab = qubits
    if chi == 'trace_chi':
        qubits_lab = qubits
    elif chi == 'weighted_fidelity_chi':
        qubits_lab = 1

    if standard_test == True:
        data, drawing = data_generator(problem)
        if problem == 'sphere':
            test_data = data[500:]
        elif problem == 'hypersphere':
            test_data = data[1000:]
        else:
            test_data = data[:250]

    elif standard_test == False:
        test_data, drawing = data_generator(problem, samples=samples)

    if problem in ['circle', 'line', '2 lines', 'wavy circle', 'sphere', 'non convex', 'crown', 'hypersphere']:
        classes = 2
    if problem in ['tricrown']:
        classes = 3
    if problem in ['6squares']:
        classes = 6
    elif problem in ['3 circles', 'wavy lines', 'squares']:
        classes = 4

    #reprs = representatives(classes, qubits_lab)

    params = read_summary(chi, problem, qubits, entanglement, layers, method, name)

    if chi == 'fidelity_chi':
        reprs = representatives(classes, qubits_lab)
        theta, alpha = params
        sol_test, acc_test = Accuracy_test(theta, alpha, test_data, reprs, entanglement, chi)

    if chi == 'trace_chi':
        reprs = representatives_tr(classes, qubits_lab)
        theta, alpha = params
        sol_test, acc_test = Accuracy_test(theta, alpha, test_data, reprs, entanglement, chi)

    if chi == 'weighted_fidelity_chi':
        reprs = representatives(classes, qubits_lab)
        theta, alpha, weight = params
        sol_test, acc_test = Accuracy_test(theta, alpha, test_data, reprs,
                                    entanglement, chi, weights=weight)

    foldname = name_folder(chi, problem, qubits, entanglement, layers, method)
    angles = np.zeros((len(sol_test), 2))
    for i, x in enumerate(sol_test[:, :2]):
        theta_aux = code_coords(theta, alpha, x)
        C = circuit(theta_aux, entanglement)
        angles[i, 0] = np.arccos(np.abs(C.psi[0])**2 - np.abs(C.psi[1])**2) - np.pi/2
        angles[i, 1] = np.angle(C.psi[1] / C.psi[0])
        print(angles[i])

    if bw == False:
        colors_classes = get_cmap('plasma')
        norm_class = Normalize(vmin=-.5, vmax=np.max(sol_test[:, -3]) + .5)

        colors_rightwrong = get_cmap('RdYlGn')
        norm_rightwrong = Normalize(vmin=-.1, vmax=1.1)

    if bw == True:
        colors_classes = get_cmap('Greys')
        norm_class = Normalize(vmin=-.1, vmax=np.max(sol[:, -3]) + .1)

        colors_rightwrong = get_cmap('Greys')
        norm_rightwrong = Normalize(vmin=-.1, vmax=1.1)

    fig, ax = plt.subplots(nrows=2)
    ax[0].plot(laea_x(np.pi, np.linspace(0, np.pi)), laea_y(np.pi, np.linspace(0, np.pi)), color='k')
    ax[0].plot(laea_x(-np.pi, np.linspace(0, -np.pi)), laea_y(-np.pi, np.linspace(0, -np.pi)), color='k')

    ax[1].plot(laea_x(np.pi, np.linspace(0, np.pi)), laea_y(np.pi, np.linspace(0, np.pi)), color='k')
    ax[1].plot(laea_x(-np.pi, np.linspace(0, -np.pi)), laea_y(-np.pi, np.linspace(0, -np.pi)), color='k')

    ax[0].scatter(laea_x(angles[:, 1], angles[:, 0]), laea_y(angles[:, 1], angles[:, 0]), c=sol_test[:, -2],

                  cmap=colors_classes, s=2, norm=norm_class)
    ax[1].scatter(laea_x(angles[:, 1], angles[:, 0]), laea_y(angles[:, 1], angles[:, 0]), c=sol_test[:,-1], cmap = colors_rightwrong, s=2, norm=norm_rightwrong)
    plt.show()
'''

def SGD_step_by_step_minimization(problem, qubits, entanglement, layers, name,
                                  epochs = 3000, batch_size = 20, eta = .1, err=False):
    """
    This function creates data and minimizes whichever problem using a step by step SGD and saving all results from accuracies for training and test sets
    INPUT:
        -problem: name of the problem, to choose among
            ['circle', '3 circles', 'hypersphere', 'tricrown', 'non convex', 'crown', 'sphere', 'squares', 'wavy lines']

        -qubits: number of qubits, must be an integer
        -entanglement: whether there is entanglement or not in the Ansätze, just 'y'/'n'
        -layers: number of layers, must be an integer. If layers == 1, entanglement is not taken in account

        -method: minimization method, to choose among ['SGD', another valid for function scipy.optimize.minimize]

        -name: a name we want for our our files to be save with
        -seed: seed of numpy.random, needed for replicating results
        -epochs: number of epochs for a 'SGD' method. If there is another method, this input has got no importance

        -batch_size: size of the batches for stochastic gradient descent, only for 'SGD' method
        -eta: learning rate, only for 'SGD' method
    OUTPUT:
        This function has got no outputs, but several files are saved in an appropiate folder. The files are

        -summary.txt: Saves useful information for the problem
        -theta.txt: saves the theta parameters as a flat array
        -alpha.txt: saves the alpha parameters as a flat array
        -error_rates: accuracies for training and test sets as flat arrays
    """
    chi = 'fidelity_chi'
    method = 'SGD'


    data, drawing = data_generator(problem, err=err)
    if problem == 'sphere':
        train_data = data[:500]
        test_data = data[500:]
    elif problem == 'hypersphere':
        train_data = data[:1000]
        test_data = data[1000:]
    else:
        train_data = data[:250]
        test_data = data[250:]

    if chi == 'fidelity_chi':
        qubits_lab = qubits
    if chi == 'trace_chi':
        qubits_lab = qubits
    elif chi == 'weighted_fidelity_chi':
        qubits_lab = 1

    theta, alpha, reprs = problem_generator(problem, qubits, layers, chi,
                                            qubits_lab=qubits_lab)
    accs_test=[]
    accs_train=[]
    chis=[]
    acc_test_sol = 0
    acc_train_sol = 0
    fid_sol = 0
    best_epoch = 0
    theta_sol = theta.copy()
    alpha_sol = alpha.copy()

    file_text = write_epochs_file(chi, problem, qubits, entanglement, layers, method, name)
    for e in range(epochs):
        theta, alpha, fid = fidelity_minimization(theta, alpha, train_data, reprs,
                                            entanglement, method, batch_size, eta, 1)

        acc_train = tester(theta, alpha, train_data, reprs, entanglement, chi)
        acc_test = tester(theta, alpha, test_data, reprs, entanglement, chi)
        accs_test.append(acc_test)
        accs_train.append(acc_train)
        chis.append(fid)

        write_epoch(file_text, e, theta, alpha, fid, acc_train, acc_test)

        if acc_test > acc_test_sol:

            acc_test_sol = acc_test
            acc_train_sol = acc_train
            fid_sol = fid
            theta_sol = theta
            alpha_sol = alpha
            best_epoch = e

    close_epochs_file(file_text, best_epoch)
    write_summary(chi, problem, qubits, entanglement, layers, method, name,
           theta_sol, alpha_sol, None, fid_sol, acc_train_sol, acc_test_sol, epochs)
    write_epochs_error_rate(chi, problem, qubits, entanglement, layers, method, name,
                      accs_train, accs_test)

def overlearning_paint(chi, problem, qubits, entanglement, layers, method, name):
    """
    This function takes overlearning functions and paints them
    INPUT:
        -chi: cost function, just 'fidelity_chi'
        -problem: name of the problem, to choose among
            ['circle', '3 circles', 'hypersphere', 'tricrown', 'non convex', 'crown', 'sphere', 'squares', 'wavy lines']

        -qubits: number of qubits, must be an integer
        -entanglement: whether there is entanglement or not in the Ansätze, just 'y'/'n'
        -layers: number of layers, must be an integer. If layers == 1, entanglement is not taken in account

        -method: minimization method, 'SGD'
        -name: a name we want for our our files to be save with
    OUTPUT:
        This function has got no outputs, but saves a picture with the information of the overlearning rates

    """
    foldname = name_folder(chi, problem, qubits, entanglement, layers, method)
    create_folder(foldname)
    filename_train = foldname + '/' + name + '_train.txt'
    filename_test = foldname + '/' + name + '_test.txt'

    train_err_rate = np.loadtxt(filename_train)
    test_err_rate = np.loadtxt(filename_test)
    fig, ax = plt.subplots()
    ax.plot(range(len(train_err_rate)), train_err_rate, label = 'Training set')
    ax.plot(range(len(test_err_rate)), test_err_rate, label = 'Test set')
    ax.set_xlabel('Epochs', fontsize=16)
    ax.set_ylabel('Error rate', fontsize=16)
    ax.legend()
    filename = foldname + '/' + name + '_overlearning'
    fig.savefig(filename)
    plt.close('all')
```

Right: original reference

```
#######################################################################
#Quantum classifier
#Adrián Pérez-Salinas, Alba Cervera-Lierta, Elies Gil, J. Ignacio Latorre
#Code by APS
#Code-checks by ACL
#June 3rd 2019


#Universitat de Barcelona / Barcelona Supercomputing Center/Institut de Ciències del Cosmos

#######################################################################

#This file is a file taking many different functions from other files and mixing them all together
# so that the usage is automatized

from data_gen import data_generator
from problem_gen import problem_generator, representatives
from fidelity_minimization import fidelity_minimization
from weighted_fidelity_minimization import weighted_fidelity_minimization
from test_data import Accuracy_test, tester
from save_data import write_summary, read_summary, name_folder, samples_paint, samples_paint_worldmap, laea_x, laea_y
from save_data import write_epochs_file, write_epoch, close_epochs_file, create_folder, write_epochs_error_rate
import numpy as np
import matplotlib.pyplot as plt
from circuitery import code_coords, circuit
from matplotlib.cm import get_cmap
from matplotlib.colors import Normalize

def minimizer(chi, problem, qubits, entanglement, layers, method, name,
              seed = 30, epochs=3000, batch_size=20,  eta=0.1):
    """
    This function creates data and minimizes whichever problem (from the selected ones)
    INPUT:
        -chi: cost function, to choose between 'fidelity_chi' or 'weighted_fidelity_chi'
        -problem: name of the problem, to choose among
            ['circle', '3 circles', 'hypersphere', 'tricrown', 'non convex', 'crown', 'sphere', 'squares', 'wavy lines']
        -qubits: number of qubits, must be an integer
        -entanglement: whether there is entanglement or not in the Ansätze, just 'y'/'n'
        -layers: number of layers, must be an integer. If layers == 1, entanglement is not taken in account
        -method: minimization method, to choose among ['SGD', another valid for function scipy.optimize.minimize]
        -name: a name we want for our our files to be save with
        -seed: seed of numpy.random, needed for replicating results
        -epochs: number of epochs for a 'SGD' method. If there is another method, this input has got no importance
        -batch_size: size of the batches for stochastic gradient descent, only for 'SGD' method
        -eta: learning rate, only for 'SGD' method
    OUTPUT:
        This function has got no outputs, but several files are saved in an appropiate folder. The files are
        -summary.txt: Saves useful information for the problem
        -theta.txt: saves the theta parameters as a flat array
        -alpha.txt: saves the alpha parameters as a flat array
        -weight.txt: saves the weights as a flat array if they exist
    """
    np.random.seed(seed)
    data, drawing = data_generator(problem)
    if problem == 'sphere':
        train_data = data[:500]
        test_data = data[500:]
    elif problem == 'hypersphere':
        train_data = data[:1000]
        test_data = data[1000:]
    else:
        train_data = data[:200]
        test_data = data[200:]

    if chi == 'fidelity_chi':
        qubits_lab = qubits
        theta, alpha, reprs = problem_generator(problem,qubits, layers, chi,
                                                qubits_lab=qubits_lab)
        theta, alpha, f = fidelity_minimization(theta, alpha, train_data, reprs,
                                                entanglement, method,
                                                batch_size, eta, epochs)
        acc_train = tester(theta, alpha, train_data, reprs, entanglement, chi)
        acc_test = tester(theta, alpha, test_data, reprs, entanglement, chi)
        write_summary(chi, problem, qubits, entanglement, layers, method, name,
              theta, alpha, 0, f, acc_train, acc_test, seed, epochs=epochs)
    elif chi == 'weighted_fidelity_chi':
        qubits_lab = 1
        theta, alpha, weight, reprs = problem_generator(problem,qubits, layers, chi,
                                                qubits_lab=qubits_lab)
        theta, alpha, weight, f = weighted_fidelity_minimization(theta, alpha, weight, train_data, reprs,
                                                entanglement, method)
        acc_train = tester(theta, alpha, train_data, reprs, entanglement, chi, weights=weight)
        acc_test = tester(theta, alpha, test_data, reprs, entanglement, chi, weights=weight)
        write_summary(chi, problem, qubits, entanglement, layers, method, name,
              theta, alpha, weight, f, acc_train, acc_test, seed, epochs=epochs)


def painter(chi, problem, qubits, entanglement, layers, method, name,
            seed = 30, standard_test = True, samples = 4000, bw = False, err = False):
    """
    This function takes written text files and paint the results of the problem
    INPUT:
        -chi: cost function, to choose between 'fidelity_chi' or 'weighted_fidelity_chi'
        -problem: name of the problem, to choose among
            ['circle', '3 circles', 'hypersphere', 'tricrown', 'non convex', 'crown', 'sphere', 'squares', 'wavy lines']
        -qubits: number of qubits, must be an integer
        -entanglement: whether there is entanglement or not in the Ansätze, just 'y'/'n'
        -layers: number of layers, must be an integer. If layers == 1, entanglement is not taken in account
        -method: minimization method, to choose among ['SGD', another valid for function scipy.optimize.minimize]
        -name: a name we want for our our files to be save with
        -seed: seed of numpy.random, needed for replicating results
        -standard_test: Whether we want to paint the set test used for checking when minimizing. If True, seed and samples are not taken in account
        -samples: number of samples of the test set
        -bw: painting in black and white
    OUTPUT:
        This function has got no outputs, but a file containing the representation of the test set is created
    """
    np.random.seed(seed)

    if chi == 'fidelity_chi':
        qubits_lab = qubits
    elif chi == 'weighted_fidelity_chi':
        qubits_lab = 1

    if standard_test == True:
        data, drawing = data_generator(problem)
        if problem == 'sphere':
            test_data = data[500:]
        elif problem == 'hypersphere':
            test_data = data[1000:]
        else:
            test_data = data[200:]

    elif standard_test == False:
        test_data, drawing = data_generator(problem, samples = samples)

    if problem in ['circle','wavy circle','sphere', 'non convex', 'crown', 'hypersphere']:
        classes = 2
    if problem in ['tricrown']:
        classes = 3
    elif problem in ['3 circles','wavy lines','squares']:
        classes = 4

    reprs = representatives(classes, qubits_lab)

    params = read_summary(chi, problem, qubits, entanglement, layers, method, name)

    if chi == 'fidelity_chi':
        theta, alpha = params
        sol_test, acc_test = Accuracy_test(theta, alpha, test_data, reprs, entanglement, chi)

    if chi == 'weighted_fidelity_chi':
        theta, alpha, weight = params
        sol_test, acc_test = Accuracy_test(theta, alpha, test_data, reprs,
                                    entanglement, chi, weights = weight)

    foldname = name_folder(chi, problem, qubits, entanglement, layers, method)
    samples_paint(problem, drawing, sol_test, foldname, name, bw)

def paint_world(chi, problem, qubits, entanglement, layers, method, name,
        seed = 30, standard_test = True, samples = 4000, bw = False, err = False):
    np.random.seed(seed)

    if chi == 'fidelity_chi':
        qubits_lab = qubits
    elif chi == 'weighted_fidelity_chi':
        qubits_lab = 1

    if standard_test == True:
        data, drawing = data_generator(problem)
        if problem == 'sphere':
            test_data = data[500:]
        elif problem == 'hypersphere':
            test_data = data[1000:]
        else:
            test_data = data[200:]

    elif standard_test == False:
        test_data, drawing = data_generator(problem, samples=samples)

    if problem in ['circle', 'wavy circle', 'sphere', 'non convex', 'crown', 'hypersphere']:
        classes = 2
    if problem in ['tricrown']:
        classes = 3
    elif problem in ['3 circles', 'wavy lines', 'squares']:
        classes = 4

    reprs = representatives(classes, qubits_lab)

    params = read_summary(chi, problem, qubits, entanglement, layers, method, name)

    if chi == 'fidelity_chi':
        theta, alpha = params
        sol_test, acc_test = Accuracy_test(theta, alpha, test_data, reprs, entanglement, chi)

    if chi == 'weighted_fidelity_chi':
        theta, alpha, weight = params
        sol_test, acc_test = Accuracy_test(theta, alpha, test_data, reprs,
                                    entanglement, chi, weights=weight)

    foldname = name_folder(chi, problem, qubits, entanglement, layers, method)
    angles = np.zeros((len(sol_test), 2))
    for i, x in enumerate(sol_test[:, :2]):
        theta_aux = code_coords(theta, alpha, x)
        C = circuit(theta_aux, entanglement)
        angles[i, 0] = np.arccos(np.abs(C.psi[0])**2 - np.abs(C.psi[1])**2) - np.pi/2
        angles[i, 1] = np.angle(C.psi[1] / C.psi[0])
        print(angles[i])

    if bw == False:
        colors_classes = get_cmap('plasma')
        norm_class = Normalize(vmin=-.5, vmax=np.max(sol_test[:, -3]) + .5)

        colors_rightwrong = get_cmap('RdYlGn')
        norm_rightwrong = Normalize(vmin=-.1, vmax=1.1)

    if bw == True:
        colors_classes = get_cmap('Greys')
        norm_class = Normalize(vmin=-.1, vmax=np.max(sol[:, -3]) + .1)

        colors_rightwrong = get_cmap('Greys')
        norm_rightwrong = Normalize(vmin=-.1, vmax=1.1)

    fig, ax = plt.subplots(nrows=2)
    ax[0].plot(laea_x(np.pi, np.linspace(0, np.pi)), laea_y(np.pi, np.linspace(0, np.pi)), color='k')
    ax[0].plot(laea_x(-np.pi, np.linspace(0, -np.pi)), laea_y(-np.pi, np.linspace(0, -np.pi)), color='k')
    ax[1].plot(laea_x(np.pi, np.linspace(0, np.pi)), laea_y(np.pi, np.linspace(0, np.pi)), color='k')
    ax[1].plot(laea_x(-np.pi, np.linspace(0, -np.pi)), laea_y(-np.pi, np.linspace(0, -np.pi)), color='k')
    ax[0].scatter(laea_x(angles[:, 1], angles[:, 0]), laea_y(angles[:, 1], angles[:, 0]), c=sol_test[:, -2],
                  cmap=colors_classes, s=2, norm=norm_class)
    ax[1].scatter(laea_x(angles[:, 1], angles[:, 0]), laea_y(angles[:, 1], angles[:, 0]), c=sol_test[:,-1], cmap = colors_rightwrong, s=2, norm=norm_rightwrong)
    plt.show()


def SGD_step_by_step_minimization(problem, qubits, entanglement, layers, name,
                                  seed = 30, epochs = 3000, batch_size = 20, eta = .1, err=False):
    """
    This function creates data and minimizes whichever problem using a step by step SGD and saving all results from accuracies for training and test sets
    INPUT:
        -problem: name of the problem, to choose among
            ['circle', '3 circles', 'hypersphere', 'tricrown', 'non convex', 'crown', 'sphere', 'squares', 'wavy lines']
        -qubits: number of qubits, must be an integer
        -entanglement: whether there is entanglement or not in the Ansätze, just 'y'/'n'
        -layers: number of layers, must be an integer. If layers == 1, entanglement is not taken in account
        -method: minimization method, to choose among ['SGD', another valid for function scipy.optimize.minimize]
        -name: a name we want for our our files to be save with
        -seed: seed of numpy.random, needed for replicating results
        -epochs: number of epochs for a 'SGD' method. If there is another method, this input has got no importance
        -batch_size: size of the batches for stochastic gradient descent, only for 'SGD' method
        -eta: learning rate, only for 'SGD' method
    OUTPUT:
        This function has got no outputs, but several files are saved in an appropiate folder. The files are
        -summary.txt: Saves useful information for the problem
        -theta.txt: saves the theta parameters as a flat array
        -alpha.txt: saves the alpha parameters as a flat array
        -error_rates: accuracies for training and test sets as flat arrays
    """
    chi = 'fidelity_chi'
    method = 'SGD'

    np.random.seed(seed)
    data, drawing = data_generator(problem, err=err)
    if problem == 'sphere':
        train_data = data[:500]
        test_data = data[500:]
    elif problem == 'hypersphere':
        train_data = data[:1000]
        test_data = data[1000:]
    else:
        train_data = data[:200]
        test_data = data[200:]

    if chi == 'fidelity_chi':
        qubits_lab = qubits
    elif chi == 'weighted_fidelity_chi':
        qubits_lab = 1

    theta, alpha, reprs = problem_generator(problem, qubits, layers, chi,
                                            qubits_lab=qubits_lab)
    accs_test=[]
    accs_train=[]
    chis=[]
    acc_test_sol = 0
    acc_train_sol = 0
    fid_sol = 0
    best_epoch = 0
    theta_sol = theta.copy()
    alpha_sol = alpha.copy()

    file_text = write_epochs_file(chi, problem, qubits, entanglement, layers, method, name)
    for e in range(epochs):
        theta, alpha, fid = fidelity_minimization(theta, alpha, train_data, reprs,
                                            entanglement, method, batch_size, eta, 1)

        acc_train = tester(theta, alpha, train_data, reprs, entanglement, chi)
        acc_test = tester(theta, alpha, test_data, reprs, entanglement, chi)
        accs_test.append(acc_test)
        accs_train.append(acc_train)
        chis.append(fid)

        write_epoch(file_text, e, theta, alpha, fid, acc_train, acc_test)

        if acc_test > acc_test_sol:

            acc_test_sol = acc_test
            acc_train_sol = acc_train
            fid_sol = fid
            theta_sol = theta
            alpha_sol = alpha
            best_epoch = e

    close_epochs_file(file_text, best_epoch)
    write_summary(chi, problem, qubits, entanglement, layers, method, name,
           theta_sol, alpha_sol, None, fid_sol, acc_train_sol, acc_test_sol, seed, epochs)
    write_epochs_error_rate(chi, problem, qubits, entanglement, layers, method, name,
                      accs_train, accs_test)

def overlearning_paint(chi, problem, qubits, entanglement, layers, method, name):
    """
    This function takes overlearning functions and paints them
    INPUT:
        -chi: cost function, just 'fidelity_chi'
        -problem: name of the problem, to choose among
            ['circle', '3 circles', 'hypersphere', 'tricrown', 'non convex', 'crown', 'sphere', 'squares', 'wavy lines']
        -qubits: number of qubits, must be an integer
        -entanglement: whether there is entanglement or not in the Ansätze, just 'y'/'n'
        -layers: number of layers, must be an integer. If layers == 1, entanglement is not taken in account
        -method: minimization method, 'SGD'
        -name: a name we want for our our files to be save with
    OUTPUT:
        This function has got no outputs, but saves a picture with the information of the overlearning rates
    """
    foldname = name_folder(chi, problem, qubits, entanglement, layers, method)
    create_folder(foldname)
    filename_train = foldname + '/' + name + '_train.txt'
    filename_test = foldname + '/' + name + '_test.txt'

    train_err_rate = np.loadtxt(filename_train)
    test_err_rate = np.loadtxt(filename_test)
    fig, ax = plt.subplots()
    ax.plot(range(len(train_err_rate)), train_err_rate, label = 'Training set')
    ax.plot(range(len(test_err_rate)), test_err_rate, label = 'Test set')
    ax.set_xlabel('Epochs', fontsize=16)
    ax.set_ylabel('Error rate', fontsize=16)
    ax.legend()
    filename = foldname + '/' + name + '_overlearning'
    fig.savefig(filename)
    plt.close('all')
```

Text Compare

```python
# coding=utf-8
###########################################################################
#Quantum classifier
#Sara Aminpour, Mike Banad, Sarah Sharif
#September 25th 2024

#School of Electrical and Computer Engineering/ Center for Quantum and Technology, University of Oklahoma, Norman, OK 73019 USA,
###########################################################################
#IMPORTANT_NOTE:
#The code on the left was developed by Sara Aminpour, while the code on the right serves as the reference implementation by Adrián Pérez-Salinas.
#The code on the left has been restructured to handle random data. So some certain sections has been deleted from the reference code.
#Additionally, our code on the left developed to analyze trace distance cost function and linear classification problem
#as well as necessary modification to apply COBYLA, L-BFGS-B, NELDER-MEAD, and SLSQP minimization methods.
###########################################################################

## This file creates the data points for the different problems to be tackled by the quantum classifier



import numpy as np

problems = ['circle', 'line', '3 circles', 'wavy circle', 'hypersphere', 'tricrown', 'non convex', 'crown', 'sphere', 'squares', 'wavy lines']

def data_generator(problem, samples=None):
    """
    This function generates the data for a problem
    INPUT:
        -problem: Name of the problem, one of: 'circle', '3 circles', 'hypersphere', 'tricrown', 'non convex', 'crown', 'sphere', 'squares', 'wavy lines'
        -samples Number of samples for the data
    OUTPUT:
        -data: set of training and test data
        -settings: things needed for drawing
    """
    problem = problem.lower()
    if problem not in problems:
        raise ValueError('problem must be one of {}'.format(problems))
    if samples == None:
        if problem == 'sphere':
            samples = 4500
        elif problem == 'hypersphere':
            samples = 5000
        else:
            samples = 4250

    if problem == 'circle':
        data, settings = _circle(samples)

    if problem == '3 circles':
        data, settings = _3_circles(samples)

    if problem == 'wavy lines':
        data, settings = _wavy_lines(samples)

    if problem == 'squares':
        data, settings = _squares(samples)

    if problem == 'sphere':
        data, settings = _sphere(samples)

    if problem == 'non convex':
        data, settings = _non_convex(samples)

    if problem == 'crown':
        data, settings = _crown(samples)

    if problem == 'tricrown':
        data, settings = _tricrown(samples)

    if problem == 'hypersphere':
        data, settings = _hypersphere(samples)
#==========================================================================
    if problem == 'line':
        data, settings = _line(samples)
#==========================================================================

    return data, settings

def _circle(samples):
    centers = np.array([[0, 0]])
    radii = np.array([np.sqrt(2/np.pi)])
    data=[]
    dim = 2
    for i in range(samples):
        x = 2 * (np.random.rand(dim)) - 1
        y = 0
        for c, r in zip(centers, radii):
            if np.linalg.norm(x - c) < r:
                y = 1

        data.append([x, y])

    return data, (centers, radii)

def _3_circles(samples):
    centers = np.array([[-1, 1], [1, 0], [-.5, -.5]])
    radii = np.array([1, np.sqrt(6/np.pi - 1), 1/2])
    data=[]
    dim = 2
    for i in range(samples):
        x = 2 * (np.random.rand(dim)) - 1
        y = 0
        for j, (c, r) in enumerate(zip(centers, radii)):
            if np.linalg.norm(x - c) < r:
                y = j + 1

        data.append([x, y])


    return data, (centers, radii)


def _wavy_lines(samples, freq = 1):
    def fun1(s):
        return s + np.sin(freq * np.pi * s)

    def fun2(s):
        return -s + np.sin(freq * np.pi * s)
    data=[]
    dim=2
    for i in range(samples):
        x = 2 * (np.random.rand(dim)) - 1
        if x[1] < fun1(x[0]) and x[1] < fun2(x[0]): y = 0
        if x[1] < fun1(x[0]) and x[1] > fun2(x[0]): y = 1
        if x[1] > fun1(x[0]) and x[1] < fun2(x[0]): y = 2
        if x[1] > fun1(x[0]) and x[1] > fun2(x[0]): y = 3
        data.append([x, y])

    return data, freq

def _squares(samples):
    data=[]
    dim=2
    for i in range(samples):
        x = 2 * (np.random.rand(dim)) - 1
        if x[0] < 0 and x[1] < 0: y = 0
        if x[0] < 0 and x[1] > 0: y = 1
        if x[0] > 0 and x[1] < 0: y = 2
        if x[0] > 0 and x[1] > 0: y = 3
        data.append([x, y])

    return data, None

#==========================================================================
def _line(samples):
    data=[]
    dim=2
    for i in range(samples):
        x = 2 * np.random.rand(dim) -1
        #x = np.random.rand(dim)
        if x[0] < x[1] : y = 0
        if x[0] > x[1] : y = 1

        data.append([x, y])

    return data, None
#==========================================================================

def _non_convex(samples, freq = 1, x_val = 2, sin_val = 1.5):
    def fun(s):
        return -x_val * s + sin_val * np.sin(freq * np.pi * s)

    data = []
    dim = 2
    for i in range(samples):
        x = 2 * (np.random.rand(dim)) - 1
        if x[1] < fun(x[0]): y = 0
        if x[1] > fun(x[0]): y = 1
        data.append([x, y])

    return data, (freq, x_val, sin_val)

def _crown(samples):
    c = [[0,0],[0,0]]
    r = [np.sqrt(.8), np.sqrt(.8 - 2/np.pi)]
    data = []
    dim = 2
    for i in range(samples):
        x = 2 * (np.random.rand(dim)) - 1
        if np.linalg.norm(x - c[0]) < r[0] and np.linalg.norm(x - c[1]) > r[1]:
            y = 1
        else:
            y=0
        data.append([x, y])

    return data, (c, r)


def _tricrown(samples):
    centers = [[0,0],[0,0]]
    radii = [np.sqrt(.8 - 2/np.pi), np.sqrt(.8)]
    data = []
    dim = 2
    for i in range(samples):
        x = 2 * (np.random.rand(dim)) - 1
        y=0
        for j,(r,c) in enumerate(zip(radii, centers)):
            if np.linalg.norm(x - c) > r:
                y = j + 1
        data.append([x, y])

    return data, (centers, radii)

def _sphere(samples):
    centers = np.array([[0, 0, 0]])
    radii = np.array([(3/np.pi)**(1/3)])
    data=[]
    dim = 3
    for i in range(samples):
        x = 2 * (np.random.rand(dim)) - 1
        y = 0
        for c, r in zip(centers, radii):
            if np.linalg.norm(x - c) < r:
                y = 1

        data.append([x, y])

    return data, (centers, radii)

def _hypersphere(samples):
    centers = np.array([[0, 0, 0, 0]])
    radii = np.array([(2/np.pi)**(1/2)])
    data=[]
    dim = 4
    for i in range(samples):
        x = 2 * (np.random.rand(dim)) - 1
        y = 0
        for c, r in zip(centers, radii):
            if np.linalg.norm(x - c) < r:
                y = 1

        data.append([x, y])


    return data, (centers, radii)
```

```python
###########################################################################
#Quantum classifier
#Adrián Pérez-Salinas, Alba Cervera-Lierta, Elies Gil, J. Ignacio Latorre
#Code by APS
#Code-checks by ACL
#June 3rd 2019


#Universitat de Barcelona / Barcelona Supercomputing Center/Institut de Ciències del Cosmos

###########################################################################

## This file creates the data points for the different problems to be tackled by the quantum classifier



import numpy as np

problems = ['circle', '3 circles', 'wavy circle', 'hypersphere', 'tricrown', 'non convex', 'crown', 'sphere', 'squares', 'wavy lines']

def data_generator(problem, samples=None):
    """
    This function generates the data for a problem
    INPUT:
        -problem: Name of the problem, one of: 'circle', '3 circles', 'hypersphere', 'tricrown', 'non convex', 'crown', 'sphere', 'squares', 'wavy lines'
        -samples Number of samples for the data
    OUTPUT:
        -data: set of training and test data
        -settings: things needed for drawing
    """
    problem = problem.lower()
    if problem not in problems:
        raise ValueError('problem must be one of {}'.format(problems))
    if samples == None:
        if problem == 'sphere':
            samples = 4500
        elif problem == 'hypersphere':
            samples = 5000
        else:
            samples = 4200

    if problem == 'circle':
        data, settings = _circle(samples)

    if problem == '3 circles':
        data, settings = _3_circles(samples)

    if problem == 'wavy lines':
        data, settings = _wavy_lines(samples)

    if problem == 'squares':
        data, settings = _squares(samples)

    if problem == 'sphere':
        data, settings = _sphere(samples)

    if problem == 'non convex':
        data, settings = _non_convex(samples)

    if problem == 'crown':
        data, settings = _crown(samples)

    if problem == 'tricrown':
        data, settings = _tricrown(samples)

    if problem == 'hypersphere':
        data, settings = _hypersphere(samples)


    return data, settings

def _circle(samples):
    centers = np.array([[0, 0]])
    radii = np.array([np.sqrt(2/np.pi)])
    data=[]
    dim = 2
    for i in range(samples):
        x = 2 * (np.random.rand(dim)) - 1
        y = 0
        for c, r in zip(centers, radii):
            if np.linalg.norm(x - c) < r:
                y = 1

        data.append([x, y])

    return data, (centers, radii)

def _3_circles(samples):
    centers = np.array([[-1, 1], [1, 0], [-.5, -.5]])
    radii = np.array([1, np.sqrt(6/np.pi - 1), 1/2])
    data=[]
    dim = 2
    for i in range(samples):
        x = 2 * (np.random.rand(dim)) - 1
        y = 0
        for j, (c, r) in enumerate(zip(centers, radii)):
            if np.linalg.norm(x - c) < r:
                y = j + 1

        data.append([x, y])


    return data, (centers, radii)


def _wavy_lines(samples, freq = 1):
    def fun1(s):
        return s + np.sin(freq * np.pi * s)

    def fun2(s):
        return -s + np.sin(freq * np.pi * s)
    data=[]
    dim=2
    for i in range(samples):
        x = 2 * (np.random.rand(dim)) - 1
        if x[1] < fun1(x[0]) and x[1] < fun2(x[0]): y = 0
        if x[1] < fun1(x[0]) and x[1] > fun2(x[0]): y = 1
        if x[1] > fun1(x[0]) and x[1] < fun2(x[0]): y = 2
        if x[1] > fun1(x[0]) and x[1] > fun2(x[0]): y = 3
        data.append([x, y])

    return data, freq

def _squares(samples):
    data=[]
    dim=2
    for i in range(samples):
        x = 2 * (np.random.rand(dim)) - 1
        if x[0] < 0 and x[1] < 0: y = 0
        if x[0] < 0 and x[1] > 0: y = 1
        if x[0] > 0 and x[1] < 0: y = 2
        if x[0] > 0 and x[1] > 0: y = 3
        data.append([x, y])

    return data, None


def _non_convex(samples, freq = 1, x_val = 2, sin_val = 1.5):
    def fun(s):
        return -x_val * s + sin_val * np.sin(freq * np.pi * s)

    data = []
    dim = 2
    for i in range(samples):
        x = 2 * (np.random.rand(dim)) - 1
        if x[1] < fun(x[0]): y = 0
        if x[1] > fun(x[0]): y = 1
        data.append([x, y])

    return data, (freq, x_val, sin_val)

def _crown(samples):
    c = [[0,0],[0,0]]
    r = [np.sqrt(.8), np.sqrt(.8 - 2/np.pi)]
    data = []
    dim = 2
    for i in range(samples):
        x = 2 * (np.random.rand(dim)) - 1
        if np.linalg.norm(x - c[0]) < r[0] and np.linalg.norm(x - c[1]) > r[1]:
            y = 1
        else:
            y=0
        data.append([x, y])

    return data, (c, r)


def _tricrown(samples):
    centers = [[0,0],[0,0]]
    radii = [np.sqrt(.8 - 2/np.pi), np.sqrt(.8)]
    data = []
    dim = 2
    for i in range(samples):
        x = 2 * (np.random.rand(dim)) - 1
        y=0
        for j,(r,c) in enumerate(zip(radii, centers)):
            if np.linalg.norm(x - c) > r:
                y = j + 1
        data.append([x, y])

    return data, (centers, radii)

def _sphere(samples):
    centers = np.array([[0, 0, 0]])
    radii = np.array([(3/np.pi)**(1/3)])
    data=[]
    dim = 3
    for i in range(samples):
        x = 2 * (np.random.rand(dim)) - 1
        y = 0
        for c, r in zip(centers, radii):
            if np.linalg.norm(x - c) < r:
                y = 1

        data.append([x, y])

    return data, (centers, radii)

def _hypersphere(samples):
    centers = np.array([[0, 0, 0, 0]])
    radii = np.array([(2/np.pi)**(1/2)])
    data=[]
    dim = 4
    for i in range(samples):
        x = 2 * (np.random.rand(dim)) - 1
        y = 0
        for c, r in zip(centers, radii):
            if np.linalg.norm(x - c) < r:
                y = 1

        data.append([x, y])


    return data, (centers, radii)
```

Text Compare

```
#Quantum classifier
#Sara Aminpour, Mike Banad, Sarah Sharif
#September 25th 2024

#School of Electrical and Computer Engineering/ Center for Quantum and Technology, University of Oklahoma, Norman, OK 73019 USA,
###########################################################################
#IMPORTANT_NOTE:
#The code on the left was developed by Sara Aminpour, while the code on the right serves as the reference implementation by Adrián Pérez-Salinas.
#The code on the left has been restructured to handle random data. So some certain sections has been deleted from the reference code.
#Additionally, our code on the left developed to analyze trace distance cost function and linear classification problem
#as well as necessary modification to apply COBYLA, L-BFGS-B, NELDER-MEAD, and SLSQP minimization methods.
from big_functions import minimizer, painter, SGD_step_by_step_minimization, overlearning_paint
import datetime
qubits = 2  #integer, number of qubits
layers = 5 #integer, number of layers (time we reupload data)
chi = 'fidelity_chi' #Cost function; choose between ['fidelity_chi', 'trace_chi]
entanglement = 'y' #entanglement y/n
name = 'run' #However you want to name your files
seed = 30 #random seed
#epochs=3000 #number of epochs, only for SGD methods


problem=['circle', 'line'] #name of the problem, choose among ['circle', 'wavy circle', '3 circles', 'wavy lines', 'sphere', 'non convex', 'crown']
for problem in problem:

            method = ['l-bfgs-b', 'cobyla', 'nelder-mead', 'slsqp'] #minimization methods between ['l-bfgs-b', 'cobyla', 'nelder-mead', 'slsqp']
            for method in method:
                a=datetime.datetime.now()
                #SGD_step_by_step_minimization(problem, qubits, entanglement, layers, name)
                minimizer(chi, problem, qubits, entanglement, layers, method, name)
                painter(chi, problem, qubits, entanglement, layers, method, name, standard_test=True)
                #paint_world(chi, problem, qubits, entanglement, layers, method, name, standard_test=True)
                b=datetime.datetime.now()
                c=b-a

                text_file_nn = open('time.txt', mode='a+')
                text_file_nn.write(problem +'_'+ chi +'_'+ method +'_'+ str(qubits) +'Qubits_' + entanglement +'_'+ str(layers) +'Layers_' + method + "__" + 'total_time'+' = '+ str(c))
                text_file_nn.write('\n')
                text_file_nn.write('====================================================================')
                text_file_nn.write('\n')
                text_file_nn.close()
```

```
from big_functions import minimizer, painter, SGD_step_by_step_minimization, overlearning_paint, paint_world

qubits = 1  #integer, number of qubits
layers = 5 #integer, number of layers (time we reupload data)
chi = 'fidelity_chi' #Cost function; choose between ['fidelity_chi', 'weighted_fidelity_chi']
problem='wavy lines' #name of the problem, choose among ['circle', 'wavy circle', '3 circles', 'wavy lines', 'sphere', 'non convex', 'crown']
entanglement = 'y' #entanglement y/n
method = 'L-BFGS-B' #minimization methods, scipy methods or 'SGD'
name = 'run' #However you want to name your files
seed = 30 #random seed
#epochs=3000 #number of epochs, only for SGD methods

#SGD_step_by_step_minimization(problem, qubits, entanglement, layers, name)
minimizer(chi, problem, qubits, entanglement, layers, method, name, seed = seed)
painter(chi, problem, qubits, entanglement, layers, method, name, standard_test=True, seed=seed)
paint_world(chi, problem, qubits, entanglement, layers, method, name, standard_test=True, seed=seed)
```

Text Compare

```python
# coding=utf-8
##########################################################################
#Quantum classifier
#Sara Aminpour, Mike Banad, Sarah Sharif
#September 25th 2024

#School of Electrical and Computer Engineering/ Center for Quantum and Technology, University of Oklahoma, Norman, OK 73019 USA,
###########################################################################
#IMPORTANT_NOTE:
#The code on the left was developed by Sara Aminpour, while the code on the right serves as the reference implementation by Adrián Pérez-Salinas.
#The code on the left has been restructured to handle random data. So some certain sections has been deleted from the reference code.
#Additionally, our code on the left developed to analyze trace distance cost function and linear classification problem
#as well as necessary modification to apply COBYLA, L-BFGS-B, NELDER-MEAD, and SLSQP minimization methods.
###########################################################################

## This file creates the problems and their settings
import numpy as np

def problem_generator(problem, qubits, layers, chi, qubits_lab=1):
    """
    This function generates everything needed for solving the problem
    INPUT:
        -chi: cost function, to choose between 'fidelity_chi' or 'weighted_fidelity_chi'
        -problem: name of the problem, to choose among
            ['circle', '3 circles', 'hypersphere', 'tricrown', 'non convex', 'crown', 'sphere', 'squares', 'wavy lines']
        -qubits: number of qubits, must be an integer
        -layers: number of layers, must be an integer. If layers == 1, entanglement is not taken in account


    OUTPUT:
        -theta: set of parameters needed for the circuit. It is an array with shape (qubits, layers, 3)
        -alpha: set of parameters needed for the circuit. It is an array with shape (qubits, layers, dimension of data)
        -weight: set of parameters needed fot the circuit only if chi == 'weighted_fidelity_chi'. It is an array with shape (classes, qubits)
        -reprs: variable encoding the label states of the different classes
    """
    chi = chi.lower()
    if chi in ['fidelity', 'weighted_fidelity','trace']: chi += '_chi'
    if chi not in ['fidelity_chi', 'weighted_fidelity_chi','trace_chi']:
        raise ValueError('Figure of merit is not valid')

    if chi == 'weighted_fidelity_chi' and qubits_lab != 1:
        qubits_lab = 1
        print('WARNING: number of qubits for the label states has been changed to 1')

    problem = problem.lower()
    if problem == 'circle':
        theta, alpha, reprs = _circle(qubits, layers, qubits_lab, chi)
    elif problem == '3 circles':
        theta, alpha, reprs = _3_circles(qubits, layers, qubits_lab, chi)
    elif problem == 'wavy lines':
        theta, alpha, reprs = _wavy_lines(qubits, layers, qubits_lab, chi)
    elif problem == 'squares':
        theta, alpha, reprs = _squares(qubits, layers, qubits_lab, chi)
    elif problem == 'sphere':
        theta, alpha, reprs = _sphere(qubits, layers, qubits_lab, chi)
    elif problem == 'non convex':
        theta, alpha, reprs = _non_convex(qubits, layers, qubits_lab, chi)
    elif problem == 'crown':
        theta, alpha, reprs = _crown(qubits, layers, qubits_lab, chi)
    elif problem == 'tricrown':
        theta, alpha, reprs = _tricrown(qubits, layers, qubits_lab, chi)
    elif problem == 'hypersphere':
        theta, alpha, reprs = _hypersphere(qubits, layers, qubits_lab, chi)
#=========================================================================
    elif problem == 'line':
        theta, alpha, reprs = _line(qubits, layers, qubits_lab, chi)
#=========================================================================
    else:
        raise ValueError('Problem is not valid')

    if chi == 'fidelity_chi':
        return theta, alpha, reprs
    elif chi == 'trace_chi':
        return theta, alpha, reprs
    elif chi == 'weighted_fidelity_chi':
        weights = np.ones((len(reprs), qubits))
        return theta, alpha, weights, reprs

#All these are auxiliary functions for problem_generator
def _circle(qubits, layers, qubits_lab, chi):
    classes = 2
    if chi == 'trace_chi':
        reprs = representatives_tr(classes, qubits_lab)
    else:
        reprs = representatives(classes, qubits_lab)

    theta = np.random.rand(qubits, layers, 3)
    alpha = np.random.rand(qubits, layers, 2)
    return theta, alpha, reprs

def _3_circles(qubits, layers, qubits_lab, chi):
    classes = 4
    reprs = representatives(classes, qubits_lab)
    theta = np.random.rand(qubits, layers, 3)
    alpha = np.random.rand(qubits, layers, 2)
    return theta, alpha, reprs

def _wavy_lines(qubits, layers, qubits_lab, chi):
    classes = 4
    reprs = representatives(classes, qubits_lab)
    theta = np.random.rand(qubits, layers, 3)
    alpha = np.random.rand(qubits, layers, 2)
    return theta, alpha, reprs

def _squares(qubits, layers, qubits_lab, chi):
    classes = 4
    reprs = representatives(classes, qubits_lab)
    theta = np.random.rand(qubits, layers, 3)
    alpha = np.random.rand(qubits, layers, 2)
    return theta, alpha, reprs
#=========================================================================
def _line(qubits, layers, qubits_lab, chi):
    classes = 2
    if chi == 'trace_chi':
        reprs = representatives_tr(classes, qubits_lab)
    else:
        reprs = representatives(classes, qubits_lab)

    theta = np.random.rand(qubits, layers, 3)
    alpha = np.random.rand(qubits, layers, 2)
    return theta, alpha, reprs
#=========================================================================
def _non_convex(qubits, layers, qubits_lab, chi):
    classes = 2
    if chi == 'trace_chi':
        reprs = representatives_tr(classes, qubits_lab)
    else:
        reprs = representatives(classes, qubits_lab)

    theta = np.random.rand(qubits, layers, 3)
    alpha = np.random.rand(qubits, layers, 2)
    return theta, alpha, reprs

def _crown(qubits, layers, qubits_lab, chi):
    classes = 2
    if chi == 'trace_chi':
        reprs = representatives_tr(classes, qubits_lab)
    else:
        reprs = representatives(classes, qubits_lab)

    theta = np.random.rand(qubits, layers, 3)
    alpha = np.random.rand(qubits, layers, 2)
    return theta, alpha, reprs

def _tricrown(qubits, layers, qubits_lab, chi):
    classes = 3
    reprs = representatives(classes, qubits_lab)
    theta = np.random.rand(qubits, layers, 3)
    alpha = np.random.rand(qubits, layers, 2)
    return theta, alpha, reprs

def _sphere(qubits, layers, qubits_lab, chi):
    classes = 2
    reprs = representatives(classes, qubits_lab)
    theta = np.random.rand(qubits, layers, 3)
    alpha = np.random.rand(qubits, layers, 3)
    return theta, alpha, reprs

def _hypersphere(qubits, layers, qubits_lab, chi):
    classes = 2
    reprs = representatives(classes, qubits_lab)
    theta = np.random.rand(qubits, layers, 6)
    alpha = np.random.rand(qubits, layers, 4)
    return theta, alpha, reprs


def representatives_tr(classes, qubits_lab):
    """
    This function creates the label states for the classification task
    INPUT:
        -classes: number of classes of our problem
        -qubits_lab: how many qubits will store the labels
    OUTPUT:
        -reprs: the label states
    """
    #reprs = np.zeros((classes, 2**qubits_lab), dtype = 'complex')
    reprs = np.zeros((classes, 3), dtype = 'complex')
    if qubits_lab == 1:
        if classes == 0:
            raise ValueError('Nonsense classifier')
        if classes == 1:
            raise ValueError('Nonsense classifier')
        if classes == 2:
            #reprs[0] = np.array([1, 0])
            reprs[0] = np.array([0.2938926261462367, -0.5090369604551273, 0.8090169943749473])
            reprs[1] = np.array([-0.2938926261462367, 0.5090369604551273, -0.8090169943749473])
        if classes == 3:
            reprs[0] = np.array([1, 0])
            reprs[1] = np.array([1 / 2, np.sqrt(3) / 2])
            reprs[2] = np.array([1 / 2, -np.sqrt(3) / 2])
        if classes == 4:
            reprs[0] = np.array([1, 0])
            reprs[1] = np.array([1 / np.sqrt(3), np.sqrt(2 / 3)])
            reprs[2] = np.array([1 / np.sqrt(3), np.exp(1j * 2 * np.pi / 3) * np.sqrt(2 / 3)])
            reprs[3] = np.array([1 / np.sqrt(3), np.exp(-1j * 2 * np.pi / 3) * np.sqrt(2 / 3)])
        if classes == 6:
            reprs[0] = np.array([0.2938926261462367, -0.5090369604551273, 0.8090169943749473])
            reprs[1] = np.array([-0.2938926261462367, 0.5090369604551273, -0.8090169943749473])
            reprs[2] = np.array([-0.7006292692220369, -0.4045084971874737, 0.5877852522924729])
            reprs[3] = np.array([0.7006292692220369, 0.4045084971874737, -0.5877852522924729])
            reprs[4] = np.array([0.4045084971874736, -0.7006292692220369, 0.5877852522924729])
            reprs[5] = np.array([0.7006292692220369, 0.4045084971874737, -0.5877852522924729])

    if qubits_lab == 2:
        if classes == 0:
            raise ValueError('Nonsense classifier')
        if classes == 1:
            raise ValueError('Nonsense classifier')
        if classes == 2:
            reprs[0] = np.array([0.29, -0.5, 0.8])
            reprs[1] = np.array([-0.29, 0.5, -0.8])
        if classes == 3:
            reprs[0] = np.array([1, 0, 0, 0])
            reprs[1] = np.array([0, 1, 0, 0])
            reprs[2] = np.array([0, 0, 1, 0])
        if classes == 4:
            reprs[0] = np.array([1, 0, 0, 0])
            reprs[1] = np.array([0, 1, 0, 0])
            reprs[2] = np.array([0, 0, 1, 0])
            reprs[3] = np.array([0, 0, 0, 1])

    return reprs

def representatives(classes, qubits_lab):
    """
    This function creates the label states for the classification task
    INPUT:
        -classes: number of classes of our problem
        -qubits_lab: how many qubits will store the labels
    OUTPUT:
        -reprs: the label states
    """
    reprs = np.zeros((classes, 2**qubits_lab), dtype = 'complex')
    if qubits_lab == 1:
        if classes == 0:
            raise ValueError('Nonsense classifier')
        if classes == 1:
            raise ValueError('Nonsense classifier')
        if classes == 2:
            reprs[0] = np.array([1, 0])
            reprs[1] = np.array([0, 1])
        if classes == 3:
            reprs[0] = np.array([1, 0])
            reprs[1] = np.array([1 / 2, np.sqrt(3) / 2])
            reprs[2] = np.array([1 / 2, -np.sqrt(3) / 2])
        if classes == 4:
            reprs[0] = np.array([1, 0])
            reprs[1] = np.array([1 / np.sqrt(3), np.sqrt(2 / 3)])
            reprs[2] = np.array([1 / np.sqrt(3), np.exp(1j * 2 * np.pi / 3) * np.sqrt(2 / 3)])
            reprs[3] = np.array([1 / np.sqrt(3), np.exp(-1j * 2 * np.pi / 3) * np.sqrt(2 / 3)])
        if classes == 6:
            reprs[0] = np.array([1, 0])
            reprs[1] = np.array([0, 1])
            reprs[2] = 1 / np.sqrt(2) * np.array([1, 1])
            reprs[3] = 1 / np.sqrt(2) * np.array([1, -1])
            reprs[4] = 1 / np.sqrt(2) * np.array([1, 1j])
            reprs[5] = 1 / np.sqrt(2) * np.array([1, -1j])

    if qubits_lab == 2:
        if classes == 0:
            raise ValueError('Nonsense classifier')
        if classes == 1:
            raise ValueError('Nonsense classifier')
        if classes == 2:
            reprs[0] = np.array([1, 0, 0, 0])
            reprs[1] = np.array([0, 0, 0, 1])
        if classes == 3:
            reprs[0] = np.array([1, 0, 0, 0])
            reprs[1] = np.array([0, 1, 0, 0])
            reprs[2] = np.array([0, 0, 1, 0])
        if classes == 4:
            reprs[0] = np.array([1, 0, 0, 0])
            reprs[1] = np.array([0, 1, 0, 0])
            reprs[2] = np.array([0, 0, 1, 0])
            reprs[3] = np.array([0, 0, 0, 1])

    return reprs
```

```python
##########################################################################
#Quantum classifier
#Adrián Pérez-Salinas, Alba Cervera-Lierta, Elies Gil, J. Ignacio Latorre
#Code by APS
#Code-checks by ACL
#June 3rd 2019


#Universitat de Barcelona / Barcelona Supercomputing Center/Institut de Ciències del Cosmos

###########################################################################

## This file creates the problems and their settings
import numpy as np

def problem_generator(problem, qubits, layers, chi, qubits_lab=1):
    """
    This function generates everything needed for solving the problem
    INPUT:
        -chi: cost function, to choose between 'fidelity_chi' or 'weighted_fidelity_chi'
        -problem: name of the problem, to choose among
            ['circle', '3 circles', 'hypersphere', 'tricrown', 'non convex', 'crown', 'sphere', 'squares', 'wavy lines']
        -qubits: number of qubits, must be an integer
        -layers: number of layers, must be an integer. If layers == 1, entanglement is not taken in account


    OUTPUT:
        -theta: set of parameters needed for the circuit. It is an array with shape (qubits, layers, 3)
        -alpha: set of parameters needed for the circuit. It is an array with shape (qubits, layers, dimension of data)
        -weight: set of parameters needed fot the circuit only if chi == 'weighted_fidelity_chi'. It is an array with shape (classes, qubits)
        -reprs: variable encoding the label states of the different classes
    """
    chi = chi.lower()
    if chi in ['fidelity', 'weighted_fidelity']: chi += '_chi'
    if chi not in ['fidelity_chi', 'weighted_fidelity_chi']:
        raise ValueError('Figure of merit is not valid')

    if chi == 'weighted_fidelity_chi' and qubits_lab != 1:
        qubits_lab = 1
        print('WARNING: number of qubits for the label states has been changed to 1')

    problem = problem.lower()
    if problem == 'circle':
        theta, alpha, reprs = _circle(qubits, layers, qubits_lab, chi)
    elif problem == '3 circles':
        theta, alpha, reprs = _3_circles(qubits, layers, qubits_lab, chi)
    elif problem == 'wavy lines':
        theta, alpha, reprs = _wavy_lines(qubits, layers, qubits_lab, chi)
    elif problem == 'squares':
        theta, alpha, reprs = _squares(qubits, layers, qubits_lab, chi)
    elif problem == 'sphere':
        theta, alpha, reprs = _sphere(qubits, layers, qubits_lab, chi)
    elif problem == 'non convex':
        theta, alpha, reprs = _non_convex(qubits, layers, qubits_lab, chi)
    elif problem == 'crown':
        theta, alpha, reprs = _crown(qubits, layers, qubits_lab, chi)
    elif problem == 'tricrown':
        theta, alpha, reprs = _tricrown(qubits, layers, qubits_lab, chi)
    elif problem == 'hypersphere':
        theta, alpha, reprs = _hypersphere(qubits, layers, qubits_lab, chi)

    else:
        raise ValueError('Problem is not valid')

    if chi == 'fidelity_chi':
        return theta, alpha, reprs
    elif chi == 'weighted_fidelity_chi':
        weights = np.ones((len(reprs), qubits))
        return theta, alpha, weights, reprs

#All these are auxiliary functions for problem_generator
def _circle(qubits, layers, qubits_lab, chi):
    classes = 2
    reprs = representatives(classes, qubits_lab)
    theta = np.random.rand(qubits, layers, 3)
    alpha = np.random.rand(qubits, layers, 2)
    return theta, alpha, reprs

def _3_circles(qubits, layers, qubits_lab, chi):
    classes = 4
    reprs = representatives(classes, qubits_lab)
    theta = np.random.rand(qubits, layers, 3)
    alpha = np.random.rand(qubits, layers, 2)
    return theta, alpha, reprs

def _wavy_lines(qubits, layers, qubits_lab, chi):
    classes = 4
    reprs = representatives(classes, qubits_lab)
    theta = np.random.rand(qubits, layers, 3)
    alpha = np.random.rand(qubits, layers, 2)
    return theta, alpha, reprs

def _squares(qubits, layers, qubits_lab, chi):
    classes = 4
    reprs = representatives(classes, qubits_lab)
    theta = np.random.rand(qubits, layers, 3)
    alpha = np.random.rand(qubits, layers, 2)
    return theta, alpha, reprs

def _non_convex(qubits, layers, qubits_lab, chi):
    classes = 2
    reprs = representatives(classes, qubits_lab)
    theta = np.random.rand(qubits, layers, 3)
    alpha = np.random.rand(qubits, layers, 2)
    return theta, alpha, reprs

def _crown(qubits, layers, qubits_lab, chi):
    classes = 2
    reprs = representatives(classes, qubits_lab)
    theta = np.random.rand(qubits, layers, 3)
    alpha = np.random.rand(qubits, layers, 2)
    return theta, alpha, reprs

def _tricrown(qubits, layers, qubits_lab, chi):
    classes = 3
    reprs = representatives(classes, qubits_lab)
    theta = np.random.rand(qubits, layers, 3)
    alpha = np.random.rand(qubits, layers, 2)
    return theta, alpha, reprs

def _sphere(qubits, layers, qubits_lab, chi):
    classes = 2
    reprs = representatives(classes, qubits_lab)
    theta = np.random.rand(qubits, layers, 3)
    alpha = np.random.rand(qubits, layers, 3)
    return theta, alpha, reprs

def _hypersphere(qubits, layers, qubits_lab, chi):
    classes = 2
    reprs = representatives(classes, qubits_lab)
    theta = np.random.rand(qubits, layers, 6)
    alpha = np.random.rand(qubits, layers, 4)
    return theta, alpha, reprs

def representatives(classes, qubits_lab):
    """
    This function creates the label states for the classification task
    INPUT:
        -classes: number of classes of our problem
        -qubits_lab: how many qubits will store the labels
    OUTPUT:
        -reprs: the label states
    """
    reprs = np.zeros((classes, 2**qubits_lab), dtype = 'complex')
    if qubits_lab == 1:
        if classes == 0:
            raise ValueError('Nonsense classifier')
        if classes == 1:
            raise ValueError('Nonsense classifier')
        if classes == 2:
            reprs[0] = np.array([1, 0])
            reprs[1] = np.array([0, 1])
        if classes == 3:
            reprs[0] = np.array([1, 0])
            reprs[1] = np.array([1 / 2, np.sqrt(3) / 2])
            reprs[2] = np.array([1 / 2, -np.sqrt(3) / 2])
        if classes == 4:
            reprs[0] = np.array([1, 0])
            reprs[1] = np.array([1 / np.sqrt(3), np.sqrt(2 / 3)])
            reprs[2] = np.array([1 / np.sqrt(3), np.exp(1j * 2 * np.pi / 3) * np.sqrt(2 / 3)])
            reprs[3] = np.array([1 / np.sqrt(3), np.exp(-1j * 2 * np.pi / 3) * np.sqrt(2 / 3)])
        if classes == 6:
            reprs[0] = np.array([1, 0])
            reprs[1] = np.array([0, 1])
            reprs[2] = 1 / np.sqrt(2) * np.array([1, 1])
            reprs[3] = 1 / np.sqrt(2) * np.array([1, -1])
            reprs[4] = 1 / np.sqrt(2) * np.array([1, 1j])
            reprs[5] = 1 / np.sqrt(2) * np.array([1, -1j])

    if qubits_lab == 2:
        if classes == 0:
            raise ValueError('Nonsense classifier')
        if classes == 1:
            raise ValueError('Nonsense classifier')
        if classes == 2:
            reprs[0] = np.array([1, 0, 0, 0])
            reprs[1] = np.array([0, 0, 0, 1])
        if classes == 3:
            reprs[0] = np.array([1, 0, 0, 0])
            reprs[1] = np.array([0, 1, 0, 0])
            reprs[2] = np.array([0, 0, 1, 0])
        if classes == 4:
            reprs[0] = np.array([1, 0, 0, 0])
            reprs[1] = np.array([0, 1, 0, 0])
            reprs[2] = np.array([0, 0, 1, 0])
            reprs[3] = np.array([0, 0, 0, 1])

    return reprs
```

Text Compare

```python
# coding=utf-8
##########################################################################
#Quantum classifier
#Sara Aminpour, Mike Banad, Sarah Sharif
#September 25th 2024

#School of Electrical and Computer Engineering/ Center for Quantum and Technology, University of Oklahoma, Norman, OK 73019 USA,
###########################################################################
#IMPORTANT_NOTE:
#The code on the left was developed by Sara Aminpour, while the code on the right serves as the reference implementation by Adrián Pérez-Salinas.
#The code on the left has been restructured to handle random data. So some certain sections has been deleted from the reference code.
#Additionally, our code on the left developed to analyze trace distance cost function and linear classification problem

#as well as necessary modification to apply COBYLA, L-BFGS-B, NELDER-MEAD, and SLSQP minimization methods.
###########################################################################


## This is an auxiliary file. It provides the tools needed for simulating quantum
# circuits.

import numpy as np
class QCircuit(object):
    def __init__(self,qubits):
        self.num_qubits = qubits
        self.psi = [0]*2**self.num_qubits
        self.psi[0] = 1
        self.E_x=0
        self.E_y=0
        self.E_z=0
        self.r=np.array([0,0,0])

    def Ry(self,i,theta):
        if i>=self.num_qubits: raise ValueError('There are not enough qubits')
        c = np.cos(theta/2)
        s = np.sin(theta/2)
        for k in range(2**(self.num_qubits-1)):
            S = k%(2**i) + 2*(k - k%(2**i))
            S_=S + 2**i
            a=c*self.psi[S] - s*self.psi[S_];
            b=s*self.psi[S] + c*self.psi[S_];
            self.psi[S]=a; self.psi[S_]=b;

    def Rx(self,i,theta):
        if i>=self.num_qubits: raise ValueError('There are not enough qubits')
        c = np.cos(theta/2)
        s = np.sin(theta/2)
        for k in range(2**(self.num_qubits-1)):
            S = k%(2**i) + 2*(k - k%(2**i))
            S_=S + 2**i
            a=c*self.psi[S] - 1j*s*self.psi[S_];
            b=-1j*s*self.psi[S] + c*self.psi[S_];
            self.psi[S]=a; self.psi[S_]=b;

    def U2(self,i,phi,lamb):
        if i >= self.num_qubits: raise ValueError('There are not enough qubits')
        f = np.exp(1j*phi)
        l = np.exp(-1j*lamb)
        for k in range(2**(self.num_qubits-1)):
            S = k%(2**i) + 2*(k - k%(2**i))
            S_=S + 2**i
            a=1/np.sqrt(2)*(self.psi[S] - l*self.psi[S_]);
            b=1/np.sqrt(2)*(f*self.psi[S] + f*l*self.psi[S_]);
            self.psi[S]=a; self.psi[S_]=b;

    def U3(self, i, theta3):
        if i >= self.num_qubits: raise ValueError('There are not enough qubits')
        c = np.cos(theta3[0] / 2)
        s = np.sin(theta3[0] / 2)
        e_phi = np.exp(1j * theta3[1] / 2)
        e_phi_s = np.conj(e_phi)
        e_lambda = np.exp(1j * theta3[2] / 2)
        e_lambda_s = np.conj(e_lambda)

        for k in range(2 ** (self.num_qubits - 1)):
            S = k % (2 ** i) + 2 * (k - k % (2 ** i))
            S_ = S + 2 ** i
            a = c * e_phi * e_lambda * self.psi[S] - s * e_phi * e_lambda_s * self.psi[S_];

            b = s * e_phi_s * e_lambda * self.psi[S] + c * e_phi_s * e_lambda_s * self.psi[S_];

            self.psi[S] = a;
            self.psi[S_] = b;

        theta_f=np.arccos(np.abs(self.psi[S])**2 - np.abs(self.psi[S_])**2) - np.pi/2
        phi_f=np.angle(self.psi[S_] / self.psi[S])
        self.r=np.array([np.sin(theta_f)*np.cos(phi_f),np.sin(phi_f)*np.sin(theta_f),np.cos(theta_f)])

    def Rz(self,i,theta):
        if i>=self.num_qubits: raise ValueError('There are not enough qubits')
        ex = np.exp(1j*theta)
        for k in range(2**(self.num_qubits-1)):
            S = k%(2**i) + 2*(k - k%(2**i)) + 2**i
            self.psi[S]=ex*self.psi[S];

    def Hx(self,i):
        if i>=self.num_qubits: raise ValueError('There are not enough qubits')
        for k in range(2**(self.num_qubits-1)):
            S = k%(2**i) + 2*(k - k%(2**i))
            S_=S + 2**i
            a=1/np.sqrt(2)*self.psi[S] + 1/np.sqrt(2)*self.psi[S_];
            b=1/np.sqrt(2)*self.psi[S] - 1/np.sqrt(2)*self.psi[S_];
            self.psi[S] = a
            self.psi[S_] = b

    def Hy(self,i):
        if i>=self.num_qubits: raise ValueError('There are not enough qubits')
        for k in range(2**(self.num_qubits-1)):
            S = k%(2**i) + 2*(k - k%(2**i))
            S_=S + 2**i
            a =1/np.sqrt(2)*self.psi[S] -1j/np.sqrt(2)*self.psi[S_];
            b =-1j/np.sqrt(2)*self.psi[S] + 1/np.sqrt(2)*self.psi[S_];
            self.psi[S] = a
            self.psi[S_] = b

    def HyT(self,i):
        if i>=self.num_qubits: raise ValueError('There are not enough qubits')
        for k in range(2**(self.num_qubits-1)):
            S = k%(2**i) + 2*(k - k%(2**i))
            S_=S + 2**i
            a=1/np.sqrt(2)*self.psi[S] +1j/np.sqrt(2)*self.psi[S_];
            b=1j/np.sqrt(2)*self.psi[S] + 1/np.sqrt(2)*self.psi[S_];
            self.psi[S]=a; self.psi[S_]=b;

    def Cz(self,i,j):
        if i>=self.num_qubits: raise ValueError('There are not enough qubits')
        if j>=self.num_qubits: raise ValueError('There are not enough qubits')
        if i==j: raise ValueError('Control and target qubits are the same')
        if j<i: a=i; i=j; j=a;
        for k in range(2**(self.num_qubits-2)):
            S = k%2**i + (
               ( k - k%2**i)*2)%2**j + 2*(
                      (k-k%2**i)*2-((2*(k-k%2**i))%2**j)) + 2**i + 2**j;
            self.psi[S]=-self.psi[S]

    def SWAP(self,i,j):
        if i>=self.num_qubits: raise ValueError('There are not enough qubits')
        if j>=self.num_qubits: raise ValueError('There are not enough qubits')
        if i==j: raise ValueError('Control and target qubits are the same')
        for k in range(2**(self.num_qubits-2)):
            S = k%2**i + (
               ( k - k%2**i)*2)%2**j + 2*(
                      (k-k%2**i)*2-((2*(k-k%2**i))%2**j)) + 2**j;
            S_ = S + 2**i - 2**j
            a=self.psi[S_]
            self.psi[S_] = self.psi[S]
            self.psi[S] = a


    def Cx(self,i,j):
        #i = control
        #j = target
        if i>=self.num_qubits: raise ValueError('There are not enough qubits')
        if j>=self.num_qubits: raise ValueError('There are not enough qubits')
        if i==j: raise ValueError('Control and target qubits are the same')
        for k in range(2**(self.num_qubits-2)):
            S = k%2**i + (
               ( k - k%2**i)*2)%2**j + 2*(
                      (k-k%2**i)*2-((2*(k-k%2**i))%2**j)) + 2**i;
            S_ = S + 2**j
            '''
            a=self.psi[S_]
            self.psi[S_] = self.psi[S]
            self.psi[S] = a
            '''
            self.psi[S],self.psi[S_] = self.psi[S_],self.psi[S]
    def Cy(self,i,j):
        if i>=self.num_qubits: raise ValueError('There are not enough qubits')
        if j>=self.num_qubits: raise ValueError('There are not enough qubits')
        if i==j: raise ValueError('Control and target qubits are the same')
        for k in range(2**(self.num_qubits-2)):
            S = k%2**i + (
               ( k - k%2**i)*2)%2**j + 2*(
                      (k-k%2**i)*2-((2*(k-k%2**i))%2**j)) + 2**i;
            S_ = S + 2**j
            self.psi[S],self.psi[S_] = 1j*self.psi[S_],-1j*self.psi[S]

    def MeasureZ(self):
        self.E_z = 0;
        for h in range(2 ** self.num_qubits):
            s = np.binary_repr(h, width=self.num_qubits)
            self.E_z += np.abs(self.psi[h])**2*(s.count('1')-s.count('0'))

    def MeasureX(self):
        self.E_x = 0;
        for i in range(self.num_qubits):
            self.Hx(i);
        for h in range(2 ** self.num_qubits):
            s = np.binary_repr(h, width=self.num_qubits)
            self.E_x += np.abs(self.psi[h])**2*(s.count('1')-s.count('0'))
        for i in range(self.num_qubits):
            self.Hx(i);

    def MeasureY(self):
        self.E_y = 0;
        for i in range(self.num_qubits):
            self.Hy(i);
        for h in range(2 ** self.num_qubits):
            s = np.binary_repr(h, width=self.num_qubits)
            self.E_y += np.abs(self.psi[h])**2*(s.count('1')-s.count('0'))
        for i in range(self.num_qubits):
            self.HyT(i);

    def reduced_density_matrix(self, q):
        rho = np.zeros((2,2), dtype='complex')
        for i in range(2):
            for j in range(i + 1):
                for k in range(2**(self.num_qubits-1)):
                    S = k%(2**q) + 2*(k - k%(2**q))
                    rho[i,j] += self.psi[S + i*2**q] * np.conj(self.psi[S + j*2**q])
                rho[j,i] = np.conj(rho[i,j])
        return rho
```

```python
##########################################################################
#Quantum classifier
#Adrián Pérez-Salinas, Alba Cervera-Lierta, Elies Gil, J. Ignacio Latorre
#Code by APS
#Code-checks by ACL
#June 3rd 2019


#Universitat de Barcelona / Barcelona Supercomputing Center/Institut de Ciències del Cosmos

###########################################################################


## This is an auxiliary file. It provides the tools needed for simulating quantum
# circuits.

import numpy as np
class QCircuit(object):
    def __init__(self,qubits):
        self.num_qubits = qubits
        self.psi = [0]*2**self.num_qubits
        self.psi[0] = 1
        self.E_x=0
        self.E_y=0
        self.E_z=0

    def Ry(self,i,theta):
        if i>=self.num_qubits: raise ValueError('There are not enough qubits')
        c = np.cos(theta/2)
        s = np.sin(theta/2)
        for k in range(2**(self.num_qubits-1)):
            S = k%(2**i) + 2*(k - k%(2**i))
            S_=S + 2**i
            a=c*self.psi[S] - s*self.psi[S_];
            b=s*self.psi[S] + c*self.psi[S_];
            self.psi[S]=a; self.psi[S_]=b;

    def Rx(self,i,theta):
        if i>=self.num_qubits: raise ValueError('There are not enough qubits')
        c = np.cos(theta/2)
        s = np.sin(theta/2)
        for k in range(2**(self.num_qubits-1)):
            S = k%(2**i) + 2*(k - k%(2**i))
            S_=S + 2**i
            a=c*self.psi[S] - 1j*s*self.psi[S_];
            b=-1j*s*self.psi[S] + c*self.psi[S_];
            self.psi[S]=a; self.psi[S_]=b;

    def U2(self,i,phi,lamb):
        if i >= self.num_qubits: raise ValueError('There are not enough qubits')
        f = np.exp(1j*phi)
        l = np.exp(-1j*lamb)
        for k in range(2**(self.num_qubits-1)):
            S = k%(2**i) + 2*(k - k%(2**i))
            S_=S + 2**i
            a=1/np.sqrt(2)*(self.psi[S] - l*self.psi[S_]);
            b=1/np.sqrt(2)*(f*self.psi[S] + f*l*self.psi[S_]);
            self.psi[S]=a; self.psi[S_]=b;

    def U3(self, i, theta3):
        if i >= self.num_qubits: raise ValueError('There are not enough qubits')
        c = np.cos(theta3[0] / 2)
        s = np.sin(theta3[0] / 2)
        e_phi = np.exp(1j * theta3[1] / 2)
        e_phi_s = np.conj(e_phi)
        e_lambda = np.exp(1j * theta3[2] / 2)
        e_lambda_s = np.conj(e_lambda)
        for k in range(2 ** (self.num_qubits - 1)):
            S = k % (2 ** i) + 2 * (k - k % (2 ** i))
            S_ = S + 2 ** i
            a = c * e_phi * e_lambda * self.psi[S] - s * e_phi * e_lambda_s * self.psi[S_];
            b = s * e_phi_s * e_lambda * self.psi[S] + c * e_phi_s * e_lambda_s * self.psi[S_];
            self.psi[S] = a;
            self.psi[S_] = b;

    def Rz(self,i,theta):
        if i>=self.num_qubits: raise ValueError('There are not enough qubits')
        ex = np.exp(1j*theta)
        for k in range(2**(self.num_qubits-1)):
            S = k%(2**i) + 2*(k - k%(2**i)) + 2**i
            self.psi[S]=ex*self.psi[S];

    def Hx(self,i):
        if i>=self.num_qubits: raise ValueError('There are not enough qubits')
        for k in range(2**(self.num_qubits-1)):
            S = k%(2**i) + 2*(k - k%(2**i))
            S_=S + 2**i
            a=1/np.sqrt(2)*self.psi[S] + 1/np.sqrt(2)*self.psi[S_];
            b=1/np.sqrt(2)*self.psi[S] - 1/np.sqrt(2)*self.psi[S_];
            self.psi[S] = a
            self.psi[S_] = b

    def Hy(self,i):
        if i>=self.num_qubits: raise ValueError('There are not enough qubits')
        for k in range(2**(self.num_qubits-1)):
            S = k%(2**i) + 2*(k - k%(2**i))
            S_=S + 2**i
            a =1/np.sqrt(2)*self.psi[S] -1j/np.sqrt(2)*self.psi[S_];
            b =-1j/np.sqrt(2)*self.psi[S] + 1/np.sqrt(2)*self.psi[S_];
            self.psi[S] = a
            self.psi[S_] = b

    def HyT(self,i):
        if i>=self.num_qubits: raise ValueError('There are not enough qubits')
        for k in range(2**(self.num_qubits-1)):
            S = k%(2**i) + 2*(k - k%(2**i))
            S_=S + 2**i
            a=1/np.sqrt(2)*self.psi[S] +1j/np.sqrt(2)*self.psi[S_];
            b=1j/np.sqrt(2)*self.psi[S] + 1/np.sqrt(2)*self.psi[S_];
            self.psi[S]=a; self.psi[S_]=b;

    def Cz(self,i,j):
        if i>=self.num_qubits: raise ValueError('There are not enough qubits')
        if j>=self.num_qubits: raise ValueError('There are not enough qubits')
        if i==j: raise ValueError('Control and target qubits are the same')
        if j<i: a=i; i=j; j=a;
        for k in range(2**(self.num_qubits-2)):
            S = k%2**i + (
               ( k - k%2**i)*2)%2**j + 2*(
                      (k-k%2**i)*2-((2*(k-k%2**i))%2**j)) + 2**i + 2**j;
            self.psi[S]=-self.psi[S]

    def SWAP(self,i,j):
        if i>=self.num_qubits: raise ValueError('There are not enough qubits')
        if j>=self.num_qubits: raise ValueError('There are not enough qubits')
        if i==j: raise ValueError('Control and target qubits are the same')
        for k in range(2**(self.num_qubits-2)):
            S = k%2**i + (
               ( k - k%2**i)*2)%2**j + 2*(
                      (k-k%2**i)*2-((2*(k-k%2**i))%2**j)) + 2**j;
            S_ = S + 2**i - 2**j
            a=self.psi[S_]
            self.psi[S_] = self.psi[S]
            self.psi[S] = a


    def Cx(self,i,j):
        #i = control
        #j = target
        if i>=self.num_qubits: raise ValueError('There are not enough qubits')
        if j>=self.num_qubits: raise ValueError('There are not enough qubits')
        if i==j: raise ValueError('Control and target qubits are the same')
        for k in range(2**(self.num_qubits-2)):
            S = k%2**i + (
               ( k - k%2**i)*2)%2**j + 2*(
                      (k-k%2**i)*2-((2*(k-k%2**i))%2**j)) + 2**i;
            S_ = S + 2**j
            '''
            a=self.psi[S_]
            self.psi[S_] = self.psi[S]
            self.psi[S] = a
            '''
            self.psi[S],self.psi[S_] = self.psi[S_],self.psi[S]
    def Cy(self,i,j):
        if i>=self.num_qubits: raise ValueError('There are not enough qubits')
        if j>=self.num_qubits: raise ValueError('There are not enough qubits')
        if i==j: raise ValueError('Control and target qubits are the same')
        for k in range(2**(self.num_qubits-2)):
            S = k%2**i + (
               ( k - k%2**i)*2)%2**j + 2*(
                      (k-k%2**i)*2-((2*(k-k%2**i))%2**j)) + 2**i;
            S_ = S + 2**j
            self.psi[S],self.psi[S_] = 1j*self.psi[S_],-1j*self.psi[S]

    def MeasureZ(self):
        self.E_z = 0;
        for h in range(2 ** self.num_qubits):
            s = np.binary_repr(h, width=self.num_qubits)
            self.E_z += np.abs(self.psi[h])**2*(s.count('1')-s.count('0'))

    def MeasureX(self):
        self.E_x = 0;
        for i in range(self.num_qubits):
            self.Hx(i);
        for h in range(2 ** self.num_qubits):
            s = np.binary_repr(h, width=self.num_qubits)
            self.E_x += np.abs(self.psi[h])**2*(s.count('1')-s.count('0'))
        for i in range(self.num_qubits):
            self.Hx(i);

    def MeasureY(self):
        self.E_y = 0;
        for i in range(self.num_qubits):
            self.Hy(i);
        for h in range(2 ** self.num_qubits):
            s = np.binary_repr(h, width=self.num_qubits)
            self.E_y += np.abs(self.psi[h])**2*(s.count('1')-s.count('0'))
        for i in range(self.num_qubits):
            self.HyT(i);

    def reduced_density_matrix(self, q):
        rho = np.zeros((2,2), dtype='complex')
        for i in range(2):
            for j in range(i + 1):
                for k in range(2**(self.num_qubits-1)):
                    S = k%(2**q) + 2*(k - k%(2**q))
                    rho[i,j] += self.psi[S + i*2**q] * np.conj(self.psi[S + j*2**q])
                rho[j,i] = np.conj(rho[i,j])
        return rho
```

Text Compare

```python
# coding=utf-8
###########################################################################
#Quantum classifier
#Sara Aminpour, Mike Banad, Sarah Sharif
#September 25th 2024

#School of Electrical and Computer Engineering/ Center for Quantum and Technology, University of Oklahoma, Norman, OK 73019 USA,
###########################################################################
#IMPORTANT_NOTE:
#The code on the left was developed by Sara Aminpour, while the code on the right serves as the reference implementation by Adrián Pérez-Salinas.
#The code on the left has been restructured to handle random data. So some certain sections has been deleted from the reference code.
#Additionally, our code on the left developed to analyze trace distance cost function and linear classification problem
#as well as necessary modification to apply COBYLA, L-BFGS-B, NELDER-MEAD, and SLSQP minimization methods.
###########################################################################


#This file provides useful tools for painting and saving data according to the problem,
# the minimization style, the number of qubits and layers.

import os
import numpy as np
import matplotlib.pyplot as plt
from matplotlib.cm import get_cmap
from matplotlib.colors import Normalize


def write_summary(chi, problem, qubits, entanglement, layers, method, name,
          theta, alpha, weights, chi_value, acc_train, acc_test, epochs):
    """
    This function takes some informations of a given problem and saves some text files
    with this information and the parameters which are solution of the problem
    INPUT:
        -chi: cost function, to choose between 'fidelity_chi' or 'weighted_fidelity_chi'
        -problem: name of the problem, to choose between
            ['circle', '3 circles', 'hypersphere', 'tricrown', 'non convex', 'crown', 'sphere', 'squares', 'wavy lines']
        -qubits: number of qubits, must be an integer
        -entanglement: whether there is entanglement or not in the Ansätze, just 'y'/'n'
        -layers: number of layers, must be an integer. If layers == 1, entanglement is not taken in account
        -method: minimization method, to choose among ['SGD', another valid for function scipy.optimize.minimize]
        -name: a name we want for our our files to be save with
        -theta: set of parameters needed for the circuit. Must be an array with shape (qubits, layers, 3)
        -alpha: set of parameters needed for the circuit. Must be an array with shape (qubits, layers, dimension of data)
        -weight: set of parameters needed fot the circuit only if chi == 'weighted_fidelity_chi'. Must be an array with shape (classes, qubits)
        -chi_value: Value of the cost function after minimization
        -acc_train: accuracy for the training set
        -acc_test: accuracy for the test set
        -seed: seed of numpy.random, needed for replicating results
        -epochs: number of epochs for a 'SGD' method. If there is another method, this input has got no importance

    OUTPUT:
        This function has got no outputs, but several files are saved in an appropiate folder. The files are
        -summary.txt: Saves useful information for the problem
        -theta.txt: saves the theta parameters as a flat array
        -alpha.txt: saves the alpha parameters as a flat array
        -weight.txt: saves the weights as a flat array if they exist
    """
    foldname = name_folder(chi, problem, qubits, entanglement, layers, method)
    create_folder(foldname)
    file_text = open(foldname + '/' + name + '_summary.txt','w')
    file_text.write('\nFigur of merit = '+chi)
    file_text.write('\nProblem = ' + problem)
    file_text.write('\nNumber of qubits = ' + str(qubits))


    if qubits != 1:
        file_text.write('\nEntanglement = ' + entanglement)
    file_text.write('\nNumber of layers = ' + str(layers))
    file_text.write('\nMinimization method = '+ method)

    if method == 'SGD':
        file_text.write('\nNumber of epochs = '+ str(epochs))
    file_text.write('\n\nBEST RESULT\n\n')
    file_text.write('\nTHETA = \n')
    file_text.write(str(theta))
    file_text.write('\nALPHA = \n')
    file_text.write(str(alpha))


    #==========================================================================

    #==========================================================================



    if chi == 'weighted_fidelity_chi':
        file_text.write('\nWEIGHTS = \n')
        file_text.write(str(weights))
    if chi == 'weighted_trace_chi':
        file_text.write('\nWEIGHTS = \n')
        file_text.write(str(weights))
    file_text.write('\nchi**2 = ' + str(chi_value))
    file_text.write('\nacc_train = ' + str(acc_train))
    file_text.write('\nacc_test = ' + str(acc_test))
    file_text.close()

    np.savetxt(foldname + '/' + name + '_theta.txt', theta.flatten())
    np.savetxt(foldname + '/' + name + '_alpha.txt', alpha.flatten())


    if chi == 'weighted_fidelity_chi':
        np.savetxt(foldname + '/' + name + '_weight.txt', weights.flatten())

    if chi == 'weighted_trace_chi':
        np.savetxt(foldname + '/' + name + '_weight.txt', weights.flatten())



def write_summary_acc(chi, problem,layers, method, name, acc_test):

    foldname_acc = name_folder_acc(chi, layers)
    create_folder(foldname_acc)
    file_text_acc = open(foldname_acc + '/' + name + '_accuracy.txt','a+')
    file_text_acc.write('\nNEW')
    file_text_acc.write('\nProblem = ' + problem + '\n')

    file_text_acc.write('\nacc_test = ' + str(acc_test))


def read_summary(chi, problem, qubits, entanglement, layers, method, name):

    """
    This function reads the files saved by write_summary and returns theta, alpha and weight parameters
    INPUT:
        -chi: cost function, to choose between 'fidelity_chi' or 'weighted_fidelity_chi'
        -problem: name of the problem, to choose among
            ['circle', '3 circles', 'hypersphere', 'tricrown', 'non convex', 'crown', 'sphere', 'squares', 'wavy lines']
        -qubits: number of qubits, must be an integer
        -entanglement: whether there is entanglement or not in the Ansätze, just 'y'/'n'
        -layers: number of layers, must be an integer. If layers == 1, entanglement is not taken in account
        -method: minimization method, to choose among ['SGD', another valid for function scipy.optimize.minimize]
        -name: a name we want for our our files to be save with

    OUTPUT:
        -theta: set of parameters needed for the circuit. It is an array with shape (qubits, layers, 3)
        -alpha: set of parameters needed for the circuit. It is an array with shape (qubits, layers, dimension of data)
        -weight: set of parameters needed fot the circuit only if chi == 'weighted_fidelity_chi'. It is an array with shape (classes, qubits)
    """
    chi = chi.lower().replace(' ','_')
    if chi in ['fidelity', 'weighted_fidelity', 'trace', 'weighted_trace']: chi += '_chi'
    if chi not in ['fidelity_chi', 'weighted_fidelity_chi','trace_chi', 'weighted_trace_chi']:
        raise ValueError('Figure of merit is not valid')
    if chi == 'fidelity_chi':
        foldname = name_folder(chi, problem, qubits, entanglement, layers, method)
        if problem in ['circle', 'line', '2 lines', '6squares', '3 circles', 'wavy circles', 'wavy lines', 'non convex','crown','tricrown','squares']:
            theta = np.loadtxt(foldname + '/' + name + '_theta.txt').reshape((qubits, layers, 3))
            dim = 2
        elif problem == 'sphere':
            theta = np.loadtxt(foldname + '/' + name + '_theta.txt').reshape((qubits, layers, 3))
            dim = 3
        elif problem in ['hypersphere']:
            theta = np.loadtxt(foldname + '/' + name + '_theta.txt').reshape((qubits, layers, 6))
            dim = 4

        alpha = np.loadtxt(foldname + '/' + name + '_alpha.txt').reshape((qubits, layers, dim))
        return theta, alpha
#==========================================================================
#Sara
    if chi == 'trace_chi':
        foldname = name_folder(chi, problem, qubits, entanglement, layers, method)
        if problem in ['circle', 'line', '2 lines', '6squares', '3 circles', 'wavy circles', 'wavy lines', 'non convex','crown','tricrown','squares']:
            theta = np.loadtxt(foldname + '/' + name + '_theta.txt').reshape((qubits, layers, 3))
            dim = 2
        elif problem == 'sphere':
            theta = np.loadtxt(foldname + '/' + name + '_theta.txt').reshape((qubits, layers, 3))
            dim = 3
        elif problem in ['hypersphere']:
            theta = np.loadtxt(foldname + '/' + name + '_theta.txt').reshape((qubits, layers, 6))
            dim = 4

        alpha = np.loadtxt(foldname + '/' + name + '_alpha.txt').reshape((qubits, layers, dim))
        return theta, alpha
#Sara
#==========================================================================
    if chi == 'weighted_fidelity_chi':
        foldname = name_folder(chi, problem, qubits, entanglement, layers, method)
        if problem in ['circle', 'line', '2 lines', '6squares', '3 circles', 'wavy circles', 'wavy lines', 'non convex','crown','tricrown','squares']:
            theta = np.loadtxt(foldname + '/' + name + '_theta.txt').reshape((qubits, layers, 3))
            dim = 2
        elif problem == 'sphere':
            theta = np.loadtxt(foldname + '/' + name + '_theta.txt').reshape((qubits, layers, 3))
            dim = 3
        elif problem in ['hypersphere']:
            theta = np.loadtxt(foldname + '/' + name + '_theta.txt').reshape((qubits, layers, 6))
            dim = 4

        alpha = np.loadtxt(foldname + '/' + name + '_alpha.txt').reshape((qubits, layers, dim))

        if problem in ['3 circles','wavy lines','squares']:
            weight = np.loadtxt(foldname + '/' + name + '_weight.txt').reshape((4, qubits))
        if problem in ['circle', 'line', '2 lines', 'wavy circle','sphere', 'non convex', 'crown', 'hypersphere']:
            weight = np.loadtxt(foldname + '/' + name + '_weight.txt').reshape((2, qubits))
        if problem in ['tricrown']:
            weight = np.loadtxt(foldname + '/' + name + '_weight.txt').reshape((3, qubits))
        if problem in ['6squares']:
            weight = np.loadtxt(foldname + '/' + name + '_weight.txt').reshape((6, qubits))
        return theta, alpha, weight

#==========================================================================
#Sara
    if chi == 'weighted_trace_chi':
        foldname = name_folder(chi, problem, qubits, entanglement, layers, method)
        if problem in ['circle', 'line', '2 lines', '6squares', '3 circles', 'wavy circles', 'wavy lines', 'non convex','crown','tricrown','squares']:
            theta = np.loadtxt(foldname + '/' + name + '_theta.txt').reshape((qubits, layers, 3))
            dim = 2
        elif problem == 'sphere':
            theta = np.loadtxt(foldname + '/' + name + '_theta.txt').reshape((qubits, layers, 3))
            dim = 3
        elif problem in ['hypersphere']:
            theta = np.loadtxt(foldname + '/' + name + '_theta.txt').reshape((qubits, layers, 6))
            dim = 4

        alpha = np.loadtxt(foldname + '/' + name + '_alpha.txt').reshape((qubits, layers, dim))

        if problem in ['3 circles','wavy lines','squares']:
            weight = np.loadtxt(foldname + '/' + name + '_weight.txt').reshape((4, qubits))
        if problem in ['circle', 'line', '2 lines', 'wavy circle','sphere', 'non convex', 'crown', 'hypersphere']:
            weight = np.loadtxt(foldname + '/' + name + '_weight.txt').reshape((2, qubits))
        if problem in ['tricrown']:
            weight = np.loadtxt(foldname + '/' + name + '_weight.txt').reshape((3, qubits))
        if problem in ['6squares']:
            weight = np.loadtxt(foldname + '/' + name + '_weight.txt').reshape((6, qubits))
        return theta, alpha, weight


def write_epochs_file_acc(chi, layers, name):

    """
    This function creates a text file for saving data only in the SGD_step_by_step function
    INPUT:
        -chi: cost function, to choose between 'fidelity_chi' or 'weighted_fidelity_chi'
        -problem: name of the problem, to choose among
            ['circle', '3 circles', 'hypersphere', 'tricrown', 'non convex', 'crown', 'sphere', 'squares', 'wavy lines']
        -qubits: number of qubits, must be an integer
        -entanglement: whether there is entanglement or not in the Ansätze, just 'y'/'n'
        -layers: number of layers, must be an integer. If layers == 1, entanglement is not taken in account
        -method: minimization method, to choose among ['SGD', another valid for function scipy.optimize.minimize]
        -name: a name we want for our our files to be save with
    OUTPUT:
        -file_text: an object which is an open textfile ready to be used
    """
    foldname_acc = name_folder_acc(chi, layers)
    create_folder(foldname_acc)
    filename_acc = foldname_acc + '/' + name + '_epochs.txt'
    file_text_acc = open(filename_acc,'a+')
    return file_text_acc
#Sara
#==========================================================================
def write_epochs_file(chi, problem, qubits, entanglement, layers, method, name):

    """
    This function creates a text file for saving data only in the SGD_step_by_step function
    INPUT:
        -chi: cost function, to choose between 'fidelity_chi' or 'weighted_fidelity_chi'
        -problem: name of the problem, to choose among
            ['circle', '3 circles', 'hypersphere', 'tricrown', 'non convex', 'crown', 'sphere', 'squares', 'wavy lines']
        -qubits: number of qubits, must be an integer
        -entanglement: whether there is entanglement or not in the Ansätze, just 'y'/'n'
        -layers: number of layers, must be an integer. If layers == 1, entanglement is not taken in account
        -method: minimization method, to choose among ['SGD', another valid for function scipy.optimize.minimize]
        -name: a name we want for our our files to be save with
    OUTPUT:
        -file_text: an object which is an open textfile ready to be used
    """
    foldname = name_folder(chi, problem, qubits, entanglement, layers, method)
    create_folder(foldname)
    filename = foldname + '/' + name + '_epochs.txt'
    file_text = open(filename,'w')
    return file_text

def write_epoch(problem, file_text, epoch, theta, alpha, chi_value, acc_train, acc_test):
    """
    This function takes a text file and write information on it
    INPUT:
        -file_text: an object which is an open textfile ready to be used, output of write_epochs_file
        -epoch: the number of epoch providing this information
        -theta: set of parameters needed for the circuit. Must be an array with shape (qubits, layers, 3)
        -alpha: set of parameters needed for the circuit. Must be an array with shape (qubits, layers, dimension of data)
        -weight: set of parameters needed fot the circuit only if chi == 'weighted_fidelity_chi'. Must be an array with shape (classes, qubits)
        -chi_value: Value of the cost function after minimization
        -acc_train: accuracy for the training set
        -acc_test: accuracy for the test set
    OUTPUT:
        -file_text: with more information on it
    """

    file_text.write('\n Epoch = ' + str(epoch))
    file_text.write('\nTHETA = \n')
    file_text.write(str(theta))
    file_text.write('\nALPHA = \n')
    file_text.write(str(alpha))
    file_text.write('\n chi**2 = \n')
    file_text.write(str(chi_value))
    file_text.write('\nacc_train = \n')
    file_text.write(str(acc_train))
    file_text.write('\nacc_test = \n')
    file_text.write(str(acc_test))


def close_epochs_file(file_text, best_epoch):
    """
    This function takes a text file and closes it
    INPUT:
        -file_text: an object which is an open textfile ready to be used, output of write_epochs_file after write_epoch
        -best_epoch: the epoch with the best possible results
    OUTPUT:
        -file_text: closed
    """
    file_text.write('\n\n\nBest epoch = ' + str(best_epoch))
    file_text.close()

def write_epochs_error_rate(chi, problem, qubits, entanglement, layers, method, name,
                            accs_train, accs_test):
    """
    This function takes information from the SGD_step_by_step function and saves the accuracies for training and test sets. It is required for studying the overlearning
    INPUT:
        -chi: cost function, to choose between 'fidelity_chi' or 'weighted_fidelity_chi'
        -problem: name of the problem, to choose among
            ['circle', '3 circles', 'hypersphere', 'tricrown', 'non convex', 'crown', 'sphere', 'squares', 'wavy lines']
        -qubits: number of qubits, must be an integer
        -entanglement: whether there is entanglement or not in the Ansätze, just 'y'/'n'
        -layers: number of layers, must be an integer. If layers == 1, entanglement is not taken in account
        -method: minimization method, to choose among ['SGD', another valid for function scipy.optimize.minimize]

        -name: a name we want for our our files to be save with
        -accs_train: list or array with the accuracies of the training set for all epochs
        -accs_test: list or array with the accuracies of the test set for all epochs
    OUTPUT:
        Two files with the error rates in them
    """
    foldname = name_folder(chi, problem, qubits, entanglement, layers, method)
    create_folder(foldname)
    filename_train = foldname + '/' + name + '_train.txt'
    filename_test = foldname + '/' + name + '_test.txt'

    np.savetxt(filename_train, 1 - np.array(accs_train))
    np.savetxt(filename_test, 1 - np.array(accs_test))

def samples_paint(problem, settings, sol, foldname, filename, bw):
    """
    This function takes the problem and the points when they are already classified, and saves a picture of them

    INPUT:
        -problem: name of the problem, to choose among
            ['circle', '3 circles', 'hypersphere', 'tricrown', 'non convex', 'crown', 'sphere', 'squares', 'wavy lines']
        -settings: parameters the function needs for drawing. Provided by problem_gen.problem_gen
        -sol: solutions of the points alreafy classified
        -foldname : name of the folder where we store results
        -filename: name of the files we will produce
        -bw: black and white, True/False
    OUTPUT:
        a file with the points and their classes, and whether they are right or wrong
    """
    if bw == False:
        colors_classes = get_cmap('Dark2')
        norm_class = Normalize(vmin=-.5,vmax=np.max(sol[:,-3]) + .5)

        colors_rightwrong = get_cmap('RdYlGn')
        norm_rightwrong = Normalize(vmin=-.1,vmax=1.1)

    if bw == True:
        colors_classes = get_cmap('Greys')
        norm_class = Normalize(vmin=-.1,vmax=np.max(sol[:,-3]) + .1)

        colors_rightwrong = get_cmap('Greys')
        norm_rightwrong = Normalize(vmin=-.1,vmax=1.1)

    fig, axs = plt.subplots(ncols = 2, figsize=(10,5))
    ax = axs[0]
    if problem in ['circle', '3 circles', 'crown', 'tricrown']:
        centers, radii = settings
        for c, r in zip(centers, radii):
            ca = plt.Circle(c, r, color='k', fill=False, linewidth=2)
            ax.add_artist(ca)
    elif problem == 'wavy circle':
        centers, radii, wave, freq = settings
        phi = np.linspace(0, 2*np.pi, 1000)
        for (c,r, w, f) in zip(centers, radii, wave, freq):
            ax.plot(c[0] + r*(1 + w * np.cos(f * phi)) * np.cos(phi),
                    c[1] + r*(1 + w * np.cos(f * phi)) * np.sin(phi),
                    'k-')
    elif problem == 'wavy lines':
        freq = settings
        s = np.linspace(-1,1,100)
        ax.plot(s, np.clip(s + np.sin(freq * np.pi * s), -1, 1), 'k-')
        ax.plot(s, -s + np.sin(freq * np.pi * s), 'k-')
    elif problem == 'squares':
        freq = settings
        s = np.linspace(-1,1,10)
        ax.plot(s, np.zeros(10), 'k-')
        ax.plot(np.zeros(10), s, 'k-')
#==========================================================================
    elif problem == 'line':
        freq = settings
        #s = np.linspace(-1,1,10)
        s=np.linspace(-1,1,10)
        #ax.plot(s, np.zeros(10), 'k-')
        ax.plot(s, s, 'k-')
#==========================================================================
    elif problem == '2 lines':
        freq = settings
        s = np.linspace(-1,1,10)
        #ax.plot(s, np.zeros(10), 'k-')
        ax.plot(s, -s, 'k-')
        ax.plot(s, s, 'k-')
#==========================================================================
    elif problem == '6squares':
        freq = settings
        s = np.linspace(-1,1,10)
        ax.plot(s, np.zeros(10), 'k-')
        a=np.array([-0.33,-0.33,-0.33,-0.33,-0.33,-0.33,-0.33,-0.33,-0.33,-0.33])
        b=np.array([0.33,0.33,0.33,0.33,0.33,0.33,0.33,0.33,0.33,0.33])
        ax.plot(a, s, 'k-')
        ax.plot(b, s, 'k-')
#==========================================================================
    elif problem == 'non convex':
        freq, x_val, sin_val = settings
        s = np.linspace(-1,1,100)
        ax.plot(s, np.clip(-x_val * s + sin_val * np.sin(freq * np.pi * s), -1, 1), 'k-')

    ax.scatter(sol[:,0], sol[:,1], c=sol[:,-2], cmap = colors_classes, s=2, norm=norm_class)

    ax.set_xlabel('x', fontsize=16)
    ax.set_ylabel('y', fontsize=16)
    ax.tick_params(axis='both',labelsize=16)
    ax.set_xlim(-1, 1)
    ax.set_ylim(-1, 1)
    ax.margins(0)
    ax.axis('equal')

    bx = axs[1]
    bx.scatter(sol[:,0], sol[:,1], c=sol[:,-1], cmap = colors_rightwrong, s=2, norm=norm_rightwrong)
    if problem in ['circle', '3 circles', 'crown', 'tricrown']:
        centers, radii = settings
        for c, r in zip(centers, radii):
            ca = plt.Circle(c, r, color='k', fill=False, linewidth=2)
            bx.add_artist(ca)
    elif problem == 'wavy circle':
        centers, radii, wave, freq = settings
        phi = np.linspace(0, 2*np.pi, 1000)
        bx.plot(c[0] + r*(1 + wave * np.cos(freq * phi)) * np.cos(phi),
                c[1] + r*(1 + wave * np.cos(freq * phi)) * np.sin(phi),
                'k-')
    elif problem == 'wavy lines':
        freq = settings
        s = np.linspace(-1,1,100)
        bx.plot(s, np.clip(s + np.sin(freq * np.pi * s), -1, 1), 'k-')
        bx.plot(s, -s + np.sin(freq * np.pi * s), 'k-')

    elif problem == 'squares':
        freq = settings
        s = np.linspace(-1,1,10)
        bx.plot(s, np.zeros(10), 'k-')
        bx.plot(np.zeros(10), s, 'k-')
#==========================================================================
    elif problem == 'line':
        freq = settings
        #s = np.linspace(-1,1,10)
        s=np.linspace(-1,1,10)
        #ax.plot(s, np.zeros(10), 'k-')
        bx.plot(s, s, 'k-')
#==========================================================================
    elif problem == '2 lines':
        freq = settings
        s = np.linspace(-1,1,10)
        #ax.plot(s, np.zeros(10), 'k-')
        bx.plot(s, -s, 'k-')
        bx.plot(s, s, 'k-')
#==========================================================================
    elif problem == '6squares':
        freq = settings
        s = np.linspace(-1,1,10)
        ax.plot(s, np.zeros(10), 'k-')
        a=np.array([-0.33,-0.33,-0.33,-0.33,-0.33,-0.33,-0.33,-0.33,-0.33,-0.33])
        b=np.array([0.33,0.33,0.33,0.33,0.33,0.33,0.33,0.33,0.33,0.33])
        ax.plot(a, s, 'k-')
        ax.plot(b, s, 'k-')
#==========================================================================
    elif problem == 'non convex':
        freq, x_val, sin_val = settings
        s = np.linspace(-1,1,100)
        bx.plot(s, np.clip(-x_val * s + sin_val * np.sin(freq * np.pi * s), -1, 1), 'k-')


    bx.set_xlabel('x', fontsize=16)
    bx.tick_params(axis='x', labelsize = 16)
    bx.tick_params(axis='y', labelsize=0)
    bx.set_xlim([-1, 1])
    bx.set_ylim([-1, 1])
    bx.margins(0)
    bx.axis('equal')

    fig.savefig(foldname + '/' + filename)
    plt.close('all')

def laea_x(lamb, phi):
    return 2*np.sqrt(2) * np.cos(phi)*np.sin(lamb / 2) / np.sqrt(1 + np.cos(phi)*np.cos(lamb/2))


def laea_y(lamb, phi):
    return np.sqrt(2) * np.sin(phi) / np.sqrt(1 + np.cos(phi)*np.cos(lamb/2))


def samples_paint_worldmap(problem, settings, sol, foldname, filename, bw):
    """
    This function takes the problem and the points when they are already classified, and saves a picture of them

    INPUT:
        -problem: name of the problem, to choose among
            ['circle', '3 circles', 'hypersphere', 'tricrown', 'non convex', 'crown', 'sphere', 'squares', 'wavy lines']
        -settings: parameters the function needs for drawing. Provided by problem_gen.problem_gen
        -sol: solutions of the points alreafy classified
        -foldname : name of the folder where we store results
        -filename: name of the files we will produce
        -bw: black and white, True/False
    OUTPUT:
        a file with the points and their classes, and whether they are right or wrong
    """
    if bw == False:
        colors_classes = get_cmap('plasma')
        norm_class = Normalize(vmin=-.5,vmax=np.max(sol[:,-3]) + .5)

        colors_rightwrong = get_cmap('RdYlGn')
        norm_rightwrong = Normalize(vmin=-.1,vmax=1.1)

    if bw == True:
        colors_classes = get_cmap('Greys')
        norm_class = Normalize(vmin=-.5,vmax=np.max(sol[:,-3]) + .5)

        colors_rightwrong = get_cmap('Greys')
        norm_rightwrong = Normalize(vmin=-.1,vmax=1.1)

    fig, axs = plt.subplots(nrows = 3, figsize=(5,15))

    line1 = _winkel_map((np.linspace(-np.pi,np.pi), np.zeros(50)))
    line2 = _winkel_map((np.linspace(-np.pi,np.pi), np.ones(50)))
    line3 = _winkel_map((np.linspace(-np.pi,np.pi), -np.ones(50)))
    line4 = _winkel_map((np.zeros(50), np.linspace(-np.pi/2,.5*np.pi)))
    line5 = _winkel_map((np.pi*np.ones(50), np.linspace(-np.pi/2,.5*np.pi)))
    line6 = _winkel_map((-np.pi*np.ones(50), np.linspace(-np.pi/2,.5*np.pi)))
    ax = axs[0]
    ax.plot(line1[0], line1[1], 'k')
    ax.plot(line2[0], line2[1], 'k')
    ax.plot(line3[0], line3[1], 'k')
    ax.plot(line4[0], line4[1], 'k')
    ax.plot(line5[0], line5[1], 'k')
    ax.plot(line6[0], line6[1], 'k')

    X = np.empty((len(sol), 2))
    for i,s in enumerate(sol):
        mapped = _winkel_map(s[:2])
        X[i] = mapped

    ax.scatter(X[:,0], X[:,1], c=sol[:,-3], cmap = colors_classes, s=2, norm=norm_class)

    #ax.set_xlabel('x', fontsize=16)
    #ax.set_ylabel('y', fontsize=16)
    #ax.tick_params(axis='both',labelsize=16)
    #ax.set_xlim(-1, 1)
    #ax.set_ylim(-1, 1)
    #ax.margins(0)
    #ax.axis('equal')

    bx = axs[1]
    bx.scatter(X[:,0], X[:,1], c=sol[:,-2], cmap = colors_classes, s=2, norm=norm_class)

    cx = axs[2]
    cx.scatter(X[:,0], X[:,1], c=sol[:,-1], cmap = colors_rightwrong, s=2, norm=norm_rightwrong)

    #bx.set_xlabel('x', fontsize=16)
    #bx.tick_params(axis='x', labelsize = 16)
    #bx.tick_params(axis='y', labelsize=0)
    #bx.set_xlim([-1, 1])
    #bx.set_ylim([-1, 1])
    #bx.margins(0)
    #bx.axis('equal')

    fig.savefig(foldname + '/' + filename + '_worldmap')
    plt.close('all')

def _winkel_map(angles):

    alpha = np.arccos(np.cos(angles[1])*np.cos(angles[0] / 2))
    x = .5 * (angles[0] * 180 / np.pi + 2 * np.cos(angles[1] * np.sin(.5 * angles[0])) / np.sinc(alpha / np.pi))
    y = .5 * (angles[1] * 180 / np.pi + np.sin(angles[1])/np.sinc(alpha/np.pi))

    return np.array([x,y])


def create_folder(directory):
    """
    Auxiliar function for creating directories with name directory

    """
    try:
        if not os.path.exists(directory):
            os.makedirs(directory)
    except OSError:
        print ('Error: Creating directory. ' + directory)

def name_folder(chi, problem, qubits, entanglement, layers, method):
    """
    This function takes information from the SGD_step_by_step function and saves the accuracies for training and test sets. It is required for studying the overlearning
    INPUT:
        -chi: cost function, to choose between 'fidelity_chi' or 'weighted_fidelity_chi'
        -problem: name of the problem, to choose among
            ['circle', '3 circles', 'hypersphere', 'tricrown', 'non convex', 'crown', 'sphere', 'squares', 'wavy lines']
        -qubits: number of qubits, must be an integer
        -entanglement: whether there is entanglement or not in the Ansätze, just 'y'/'n'
        -layers: number of layers, must be an integer. If layers == 1, entanglement is not taken in account
        -method: minimization method, to choose among ['SGD', another valid for function scipy.optimize.minimize]

        -name: a name we want for our our files to be save with
        -accs_train: list or array with the accuracies of the training set for all epochs
        -accs_test: list or array with the accuracies of the test set for all epochs
    OUTPUT:
        -foldname: A name for a folder
    """
    chi = chi.lower().replace(' ','_')
    if chi in ['fidelity', 'weighted_fidelity', 'trace', 'weighted_trace']: chi += '_chi'
    if chi not in ['fidelity_chi', 'weighted_fidelity_chi', 'trace_chi', 'weighted_trace_chi']:
        raise ValueError('Figure of merit is not valid')
    foldname = chi + '/'
    problem = problem.replace(' ', '_')
    foldname += problem + '/'
    foldname += str(qubits) + '_qubits/'
    if qubits != 1:
        if entanglement.lower()[0] == 'y':
            foldname += 'entangled/'
        if entanglement.lower()[0] == 'n':
            foldname += 'not_entangled/'

    foldname += str(layers) + '_layers/'
    foldname += method

    return foldname

def name_folder_acc(chi, layers):
    """
    This function takes information from the SGD_step_by_step function and saves the accuracies for training and test sets. It is required for studying the overlearning
    INPUT:
        -chi: cost function, to choose between 'fidelity_chi' or 'weighted_fidelity_chi'
        -problem: name of the problem, to choose among
            ['circle', '3 circles', 'hypersphere', 'tricrown', 'non convex', 'crown', 'sphere', 'squares', 'wavy lines']
        -qubits: number of qubits, must be an integer
        -entanglement: whether there is entanglement or not in the Ansätze, just 'y'/'n'
        -layers: number of layers, must be an integer. If layers == 1, entanglement is not taken in account
        -method: minimization method, to choose among ['SGD', another valid for function scipy.optimize.minimize]
        -name: a name we want for our our files to be save with
        -accs_train: list or array with the accuracies of the training set for all epochs
        -accs_test: list or array with the accuracies of the test set for all epochs
    OUTPUT:
        -foldname: A name for a folder
    """
    chi = chi.lower().replace(' ','_')
    if chi in ['fidelity', 'weighted_fidelity', 'trace', 'weighted_trace']: chi += '_chi'
    if chi not in ['fidelity_chi', 'weighted_fidelity_chi', 'trace_chi', 'weighted_trace_chi']:
        raise ValueError('Figure of merit is not valid')
    foldname = chi + '/'
    foldname += str(layers) + '_layers/'

    return foldname
```

```python
###########################################################################
#Quantum classifier
#Adrián Pérez-Salinas, Alba Cervera-Lierta, Elies Gil, J. Ignacio Latorre
#Code by APS
#Code-checks by ACL
#June 3rd 2019


#Universitat de Barcelona / Barcelona Supercomputing Center/Institut de Ciències del Cosmos

###########################################################################


#This file provides useful tools for painting and saving data according to the problem,
# the minimization style, the number of qubits and layers.

import os
import numpy as np
import matplotlib.pyplot as plt
from matplotlib.cm import get_cmap
from matplotlib.colors import Normalize

def write_summary(chi, problem, qubits, entanglement, layers, method, name,
          theta, alpha, weights, chi_value, acc_train, acc_test, seed, epochs):
    """
    This function takes some informations of a given problem and saves some text files
    with this information and the parameters which are solution of the problem
    INPUT:
        -chi: cost function, to choose between 'fidelity_chi' or 'weighted_fidelity_chi'
        -problem: name of the problem, to choose between
            ['circle', '3 circles', 'hypersphere', 'tricrown', 'non convex', 'crown', 'sphere', 'squares', 'wavy lines']
        -qubits: number of qubits, must be an integer
        -entanglement: whether there is entanglement or not in the Ansätze, just 'y'/'n'
        -layers: number of layers, must be an integer. If layers == 1, entanglement is not taken in account
        -method: minimization method, to choose among ['SGD', another valid for function scipy.optimize.minimize]
        -name: a name we want for our our files to be save with
        -theta: set of parameters needed for the circuit. Must be an array with shape (qubits, layers, 3)
        -alpha: set of parameters needed for the circuit. Must be an array with shape (qubits, layers, dimension of data)
        -weight: set of parameters needed fot the circuit only if chi == 'weighted_fidelity_chi'. Must be an array with shape (classes, qubits)
        -chi_value: Value of the cost function after minimization
        -acc_train: accuracy for the training set
        -acc_test: accuracy for the test set
        -seed: seed of numpy.random, needed for replicating results
        -epochs: number of epochs for a 'SGD' method. If there is another method, this input has got no importance

    OUTPUT:
        This function has got no outputs, but several files are saved in an appropiate folder. The files are
        -summary.txt: Saves useful information for the problem
        -theta.txt: saves the theta parameters as a flat array
        -alpha.txt: saves the alpha parameters as a flat array
        -weight.txt: saves the weights as a flat array if they exist
    """
    foldname = name_folder(chi, problem, qubits, entanglement, layers, method)
    create_folder(foldname)
    file_text = open(foldname + '/' + name + '_summary.txt','w')
    file_text.write('\nFigur of merit = '+chi)
    file_text.write('\nProblem = ' + problem)
    file_text.write('\nNumber of qubits = ' + str(qubits))
    if qubits != 1:
        file_text.write('\nEntanglement = ' + entanglement)
    file_text.write('\nNumber of layers = ' + str(layers))
    file_text.write('\nMinimization method = '+ method)
    file_text.write('\nRandom seed = '+ str(seed))
    if method == 'SGD':
        file_text.write('\nNumber of epochs = '+ str(epochs))
    file_text.write('\n\nBEST RESULT\n\n')
    file_text.write('\nTHETA = \n')
    file_text.write(str(theta))
    file_text.write('\nALPHA = \n')
    file_text.write(str(alpha))
    if chi == 'weighted_fidelity_chi':
        file_text.write('\nWEIGHTS = \n')
        file_text.write(str(weights))
    file_text.write('\nchi**2 = ' + str(chi_value))
    file_text.write('\nacc_train = ' + str(acc_train))
    file_text.write('\nacc_test = ' + str(acc_test))
    file_text.close()

    np.savetxt(foldname + '/' + name + '_theta.txt', theta.flatten())
    np.savetxt(foldname + '/' + name + '_alpha.txt', alpha.flatten())
    if chi == 'weighted_fidelity_chi':
        np.savetxt(foldname + '/' + name + '_weight.txt', weights.flatten())


def read_summary(chi, problem, qubits, entanglement, layers, method, name):

    """
    This function reads the files saved by write_summary and returns theta, alpha and weight parameters
    INPUT:
        -chi: cost function, to choose between 'fidelity_chi' or 'weighted_fidelity_chi'
        -problem: name of the problem, to choose among
            ['circle', '3 circles', 'hypersphere', 'tricrown', 'non convex', 'crown', 'sphere', 'squares', 'wavy lines']
        -qubits: number of qubits, must be an integer
        -entanglement: whether there is entanglement or not in the Ansätze, just 'y'/'n'
        -layers: number of layers, must be an integer. If layers == 1, entanglement is not taken in account
        -method: minimization method, to choose among ['SGD', another valid for function scipy.optimize.minimize]
        -name: a name we want for our our files to be save with

    OUTPUT:
        -theta: set of parameters needed for the circuit. It is an array with shape (qubits, layers, 3)
        -alpha: set of parameters needed for the circuit. It is an array with shape (qubits, layers, dimension of data)
        -weight: set of parameters needed fot the circuit only if chi == 'weighted_fidelity_chi'. It is an array with shape (classes, qubits)
    """
    chi = chi.lower().replace(' ','_')
    if chi in ['fidelity', 'weighted_fidelity']: chi += '_chi'
    if chi not in ['fidelity_chi', 'weighted_fidelity_chi']:
        raise ValueError('Figure of merit is not valid')
    if chi == 'fidelity_chi':
        foldname = name_folder(chi, problem, qubits, entanglement, layers, method)
        if problem in ['circle', '3 circles', 'wavy circles', 'wavy lines', 'non convex','crown','tricrown','squares']:
            theta = np.loadtxt(foldname + '/' + name + '_theta.txt').reshape((qubits, layers, 3))
            dim = 2
        elif problem == 'sphere':
            theta = np.loadtxt(foldname + '/' + name + '_theta.txt').reshape((qubits, layers, 3))
            dim = 3
        elif problem in ['hypersphere']:
            theta = np.loadtxt(foldname + '/' + name + '_theta.txt').reshape((qubits, layers, 6))
            dim = 4

        alpha = np.loadtxt(foldname + '/' + name + '_alpha.txt').reshape((qubits, layers, dim))
        return theta, alpha

    if chi == 'weighted_fidelity_chi':
        foldname = name_folder(chi, problem, qubits, entanglement, layers, method)
        if problem in ['circle', '3 circles', 'wavy circles', 'wavy lines', 'non convex','crown','tricrown','squares']:
            theta = np.loadtxt(foldname + '/' + name + '_theta.txt').reshape((qubits, layers, 3))
            dim = 2
        elif problem == 'sphere':
            theta = np.loadtxt(foldname + '/' + name + '_theta.txt').reshape((qubits, layers, 3))
            dim = 3
        elif problem in ['hypersphere']:
            theta = np.loadtxt(foldname + '/' + name + '_theta.txt').reshape((qubits, layers, 6))
            dim = 4

        alpha = np.loadtxt(foldname + '/' + name + '_alpha.txt').reshape((qubits, layers, dim))

        if problem in ['3 circles','wavy lines','squares']:
            weight = np.loadtxt(foldname + '/' + name + '_weight.txt').reshape((4, qubits))
        if problem in ['circle','wavy circle','sphere', 'non convex', 'crown', 'hypersphere']:
            weight = np.loadtxt(foldname + '/' + name + '_weight.txt').reshape((2, qubits))
        if problem in ['tricrown']:
            weight = np.loadtxt(foldname + '/' + name + '_weight.txt').reshape((3, qubits))
        return theta, alpha, weight


def write_epochs_file(chi, problem, qubits, entanglement, layers, method, name):

    """
    This function creates a text file for saving data only in the SGD_step_by_step function
    INPUT:
        -chi: cost function, to choose between 'fidelity_chi' or 'weighted_fidelity_chi'
        -problem: name of the problem, to choose among
            ['circle', '3 circles', 'hypersphere', 'tricrown', 'non convex', 'crown', 'sphere', 'squares', 'wavy lines']
        -qubits: number of qubits, must be an integer
        -entanglement: whether there is entanglement or not in the Ansätze, just 'y'/'n'
        -layers: number of layers, must be an integer. If layers == 1, entanglement is not taken in account
        -method: minimization method, to choose among ['SGD', another valid for function scipy.optimize.minimize]
        -name: a name we want for our our files to be save with
    OUTPUT:
        -file_text: an object which is an open textfile ready to be used
    """
    foldname = name_folder(chi, problem, qubits, entanglement, layers, method)
    create_folder(foldname)
    filename = foldname + '/' + name + '_epochs.txt'
    file_text = open(filename,'w')
    return file_text

def write_epoch(file_text, epoch, theta, alpha, chi_value, acc_train, acc_test):
    """
    This function takes a text file and write information on it
    INPUT:
        -file_text: an object which is an open textfile ready to be used, output of write_epochs_file
        -epoch: the number of epoch providing this information
        -theta: set of parameters needed for the circuit. Must be an array with shape (qubits, layers, 3)
        -alpha: set of parameters needed for the circuit. Must be an array with shape (qubits, layers, dimension of data)
        -weight: set of parameters needed fot the circuit only if chi == 'weighted_fidelity_chi'. Must be an array with shape (classes, qubits)
        -chi_value: Value of the cost function after minimization
        -acc_train: accuracy for the training set
        -acc_test: accuracy for the test set
    OUTPUT:
        -file_text: with more information on it
    """
    file_text.write('\n Epoch = ' + str(epoch))
    file_text.write('\nTHETA = \n')
    file_text.write(str(theta))
    file_text.write('\nALPHA = \n')
    file_text.write(str(alpha))
    file_text.write('\n chi**2 = \n')
    file_text.write(str(chi_value))
    file_text.write('\nacc_train = \n')
    file_text.write(str(acc_train))
    file_text.write('\nacc_test = \n')
    file_text.write(str(acc_test))

def close_epochs_file(file_text, best_epoch):
    """
    This function takes a text file and closes it
    INPUT:
        -file_text: an object which is an open textfile ready to be used, output of write_epochs_file after write_epoch
        -best_epoch: the epoch with the best possible results
    OUTPUT:
        -file_text: closed
    """
    file_text.write('\n\n\nBest epoch = ' + str(best_epoch))
    file_text.close()

def write_epochs_error_rate(chi, problem, qubits, entanglement, layers, method, name,
                            accs_train, accs_test):
    """
    This function takes information from the SGD_step_by_step function and saves the accuracies for training and test sets. It is required for studying the overlearning
    INPUT:
        -chi: cost function, to choose between 'fidelity_chi' or 'weighted_fidelity_chi'
        -problem: name of the problem, to choose among
            ['circle', '3 circles', 'hypersphere', 'tricrown', 'non convex', 'crown', 'sphere', 'squares', 'wavy lines']
        -qubits: number of qubits, must be an integer
        -entanglement: whether there is entanglement or not in the Ansätze, just 'y'/'n'
        -layers: number of layers, must be an integer. If layers == 1, entanglement is not taken in account
        -method: minimization method, to choose among ['SGD', another valid for function scipy.optimize.minimize]
        -name: a name we want for our our files to be save with
        -accs_train: list or array with the accuracies of the training set for all epochs
        -accs_test: list or array with the accuracies of the test set for all epochs
    OUTPUT:
        Two files with the error rates in them
    """
    foldname = name_folder(chi, problem, qubits, entanglement, layers, method)
    create_folder(foldname)
    filename_train = foldname + '/' + name + '_train.txt'
    filename_test = foldname + '/' + name + '_test.txt'

    np.savetxt(filename_train, 1 - np.array(accs_train))
    np.savetxt(filename_test, 1 - np.array(accs_test))

def samples_paint(problem, settings, sol, foldname, filename, bw):
    """
    This function takes the problem and the points when they are already classified, and saves a picture of them
    INPUT:
        -problem: name of the problem, to choose among
            ['circle', '3 circles', 'hypersphere', 'tricrown', 'non convex', 'crown', 'sphere', 'squares', 'wavy lines']
        -settings: parameters the function needs for drawing. Provided by problem_gen.problem_gen
        -sol: solutions of the points alreafy classified
        -foldname : name of the folder where we store results
        -filename: name of the files we will produce
        -bw: black and white, True/False
    OUTPUT:
        a file with the points and their classes, and whether they are right or wrong
    """
    if bw == False:
        colors_classes = get_cmap('plasma')
        norm_class = Normalize(vmin=-.5,vmax=np.max(sol[:,-3]) + .5)

        colors_rightwrong = get_cmap('RdYlGn')
        norm_rightwrong = Normalize(vmin=-.1,vmax=1.1)

    if bw == True:
        colors_classes = get_cmap('Greys')
        norm_class = Normalize(vmin=-.1,vmax=np.max(sol[:,-3]) + .1)

        colors_rightwrong = get_cmap('Greys')
        norm_rightwrong = Normalize(vmin=-.1,vmax=1.1)

    fig, axs = plt.subplots(ncols = 2, figsize=(10,5))
    ax = axs[0]
    if problem in ['circle', '3 circles', 'crown', 'tricrown']:
        centers, radii = settings
        for c, r in zip(centers, radii):
            ca = plt.Circle(c, r, color='k', fill=False, linewidth=2)
            ax.add_artist(ca)
    elif problem == 'wavy circle':
        centers, radii, wave, freq = settings
        phi = np.linspace(0, 2*np.pi, 1000)
        for (c,r, w, f) in zip(centers, radii, wave, freq):
            ax.plot(c[0] + r*(1 + w * np.cos(f * phi)) * np.cos(phi),
                    c[1] + r*(1 + w * np.cos(f * phi)) * np.sin(phi),
                    'k-')
    elif problem == 'wavy lines':
        freq = settings
        s = np.linspace(-1,1,100)
        ax.plot(s, np.clip(s + np.sin(freq * np.pi * s), -1, 1), 'k-')
        ax.plot(s, -s + np.sin(freq * np.pi * s), 'k-')
    elif problem == 'squares':
        freq = settings
        s = np.linspace(-1,1,10)
        ax.plot(s, np.zeros(10), 'k-')
        ax.plot(np.zeros(10), s, 'k-')

    elif problem == 'non convex':
        freq, x_val, sin_val = settings
        s = np.linspace(-1,1,100)
        ax.plot(s, np.clip(-x_val * s + sin_val * np.sin(freq * np.pi * s), -1, 1), 'k-')

    ax.scatter(sol[:,0], sol[:,1], c=sol[:,-2], cmap = colors_classes, s=2, norm=norm_class)

    ax.set_xlabel('x', fontsize=16)
    ax.set_ylabel('y', fontsize=16)
    ax.tick_params(axis='both',labelsize=16)
    ax.set_xlim(-1, 1)
    ax.set_ylim(-1, 1)
    ax.margins(0)
    ax.axis('equal')

    bx = axs[1]
    bx.scatter(sol[:,0], sol[:,1], c=sol[:,-1], cmap = colors_rightwrong, s=2, norm=norm_rightwrong)
    if problem in ['circle', '3 circles', 'crown', 'tricrown']:
        centers, radii = settings
        for c, r in zip(centers, radii):
            ca = plt.Circle(c, r, color='k', fill=False, linewidth=2)
            bx.add_artist(ca)
    elif problem == 'wavy circle':
        centers, radii, wave, freq = settings
        phi = np.linspace(0, 2*np.pi, 1000)
        bx.plot(c[0] + r*(1 + wave * np.cos(freq * phi)) * np.cos(phi),
                c[1] + r*(1 + wave * np.cos(freq * phi)) * np.sin(phi),
                'k-')
    elif problem == 'wavy lines':
        freq = settings
        s = np.linspace(-1,1,100)
        bx.plot(s, np.clip(s + np.sin(freq * np.pi * s), -1, 1), 'k-')
        bx.plot(s, -s + np.sin(freq * np.pi * s), 'k-')

    elif problem == 'squares':
        freq = settings
        s = np.linspace(-1,1,10)
        bx.plot(s, np.zeros(10), 'k-')
        bx.plot(np.zeros(10), s, 'k-')

    elif problem == 'non convex':
        freq, x_val, sin_val = settings
        s = np.linspace(-1,1,100)
        bx.plot(s, np.clip(-x_val * s + sin_val * np.sin(freq * np.pi * s), -1, 1), 'k-')


    bx.set_xlabel('x', fontsize=16)
    bx.tick_params(axis='x', labelsize = 16)
    bx.tick_params(axis='y', labelsize=0)
    bx.set_xlim([-1, 1])
    bx.set_ylim([-1, 1])
    bx.margins(0)
    bx.axis('equal')

    fig.savefig(foldname + '/' + filename)
    plt.close('all')

def laea_x(lamb, phi):
    return 2*np.sqrt(2) * np.cos(phi)*np.sin(lamb / 2) / np.sqrt(1 + np.cos(phi)*np.cos(lamb/2))


def laea_y(lamb, phi):
    return np.sqrt(2) * np.sin(phi) / np.sqrt(1 + np.cos(phi)*np.cos(lamb/2))


def samples_paint_worldmap(problem, settings, sol, foldname, filename, bw):
    """
    This function takes the problem and the points when they are already classified, and saves a picture of them
    INPUT:
        -problem: name of the problem, to choose among
            ['circle', '3 circles', 'hypersphere', 'tricrown', 'non convex', 'crown', 'sphere', 'squares', 'wavy lines']
        -settings: parameters the function needs for drawing. Provided by problem_gen.problem_gen
        -sol: solutions of the points alreafy classified
        -foldname : name of the folder where we store results
        -filename: name of the files we will produce
        -bw: black and white, True/False
    OUTPUT:
        a file with the points and their classes, and whether they are right or wrong
    """
    if bw == False:
        colors_classes = get_cmap('plasma')
        norm_class = Normalize(vmin=-.5,vmax=np.max(sol[:,-3]) + .5)

        colors_rightwrong = get_cmap('RdYlGn')
        norm_rightwrong = Normalize(vmin=-.1,vmax=1.1)

    if bw == True:
        colors_classes = get_cmap('Greys')
        norm_class = Normalize(vmin=-.5,vmax=np.max(sol[:,-3]) + .5)

        colors_rightwrong = get_cmap('Greys')
        norm_rightwrong = Normalize(vmin=-.1,vmax=1.1)

    fig, axs = plt.subplots(nrows = 3, figsize=(5,15))

    line1 = _winkel_map((np.linspace(-np.pi,np.pi), np.zeros(50)))
    line2 = _winkel_map((np.linspace(-np.pi,np.pi), np.ones(50)))
    line3 = _winkel_map((np.linspace(-np.pi,np.pi), -np.ones(50)))
    line4 = _winkel_map((np.zeros(50), np.linspace(-np.pi/2,.5*np.pi)))
    line5 = _winkel_map((np.pi*np.ones(50), np.linspace(-np.pi/2,.5*np.pi)))
    line6 = _winkel_map((-np.pi*np.ones(50), np.linspace(-np.pi/2,.5*np.pi)))
    ax = axs[0]
    ax.plot(line1[0], line1[1], 'k')
    ax.plot(line2[0], line2[1], 'k')
    ax.plot(line3[0], line3[1], 'k')
    ax.plot(line4[0], line4[1], 'k')
    ax.plot(line5[0], line5[1], 'k')
    ax.plot(line6[0], line6[1], 'k')

    X = np.empty((len(sol), 2))
    for i,s in enumerate(sol):
        mapped = _winkel_map(s[:2])
        X[i] = mapped

    ax.scatter(X[:,0], X[:,1], c=sol[:,-3], cmap = colors_classes, s=2, norm=norm_class)

    #ax.set_xlabel('x', fontsize=16)
    #ax.set_ylabel('y', fontsize=16)
    #ax.tick_params(axis='both',labelsize=16)
    #ax.set_xlim(-1, 1)
    #ax.set_ylim(-1, 1)
    #ax.margins(0)
    #ax.axis('equal')

    bx = axs[1]
    bx.scatter(X[:,0], X[:,1], c=sol[:,-2], cmap = colors_classes, s=2, norm=norm_class)

    cx = axs[2]
    cx.scatter(X[:,0], X[:,1], c=sol[:,-1], cmap = colors_rightwrong, s=2, norm=norm_rightwrong)

    #bx.set_xlabel('x', fontsize=16)
    #bx.tick_params(axis='x', labelsize = 16)
    #bx.tick_params(axis='y', labelsize=0)
    #bx.set_xlim([-1, 1])
    #bx.set_ylim([-1, 1])
    #bx.margins(0)
    #bx.axis('equal')

    fig.savefig(foldname + '/' + filename + '_worldmap')
    plt.close('all')

def _winkel_map(angles):

    alpha = np.arccos(np.cos(angles[1])*np.cos(angles[0] / 2))
    x = .5 * (angles[0] * 180 / np.pi + 2 * np.cos(angles[1] * np.sin(.5 * angles[0])) / np.sinc(alpha / np.pi))
    y = .5 * (angles[1] * 180 / np.pi + np.sin(angles[1])/np.sinc(alpha/np.pi))

    return np.array([x,y])


def create_folder(directory):
    """
    Auxiliar function for creating directories with name directory

    """
    try:
        if not os.path.exists(directory):
            os.makedirs(directory)
    except OSError:
        print ('Error: Creating directory. ' + directory)

def name_folder(chi, problem, qubits, entanglement, layers, method):
    """
    This function takes information from the SGD_step_by_step function and saves the accuracies for training and test sets. It is required for studying the overlearning
    INPUT:
        -chi: cost function, to choose between 'fidelity_chi' or 'weighted_fidelity_chi'
        -problem: name of the problem, to choose among
            ['circle', '3 circles', 'hypersphere', 'tricrown', 'non convex', 'crown', 'sphere', 'squares', 'wavy lines']
        -qubits: number of qubits, must be an integer
        -entanglement: whether there is entanglement or not in the Ansätze, just 'y'/'n'
        -layers: number of layers, must be an integer. If layers == 1, entanglement is not taken in account
        -method: minimization method, to choose among ['SGD', another valid for function scipy.optimize.minimize]
        -name: a name we want for our our files to be save with
        -accs_train: list or array with the accuracies of the training set for all epochs
        -accs_test: list or array with the accuracies of the test set for all epochs
    OUTPUT:
        -foldname: A name for a folder
    """
    chi = chi.lower().replace(' ','_')
    if chi in ['fidelity', 'weighted_fidelity']: chi += '_chi'
    if chi not in ['fidelity_chi', 'weighted_fidelity_chi']:
        raise ValueError('Figure of merit is not valid')
    foldname = chi + '/'
    problem = problem.replace(' ', '_')
    foldname += problem + '/'
    foldname += str(qubits) + '_qubits/'
    if qubits != 1:
        if entanglement.lower()[0] == 'y':
            foldname += 'entangled/'
        if entanglement.lower()[0] == 'n':
            foldname += 'not_entangled/'

    foldname += str(layers) + '_layers/'
    foldname += method

    return foldname
```

Text Compare

```
# coding=utf-8
##########################################################################
#Quantum classifier
#Sara Aminpour, Mike Banad, Sarah Sharif
#September 25th 2024

#School of Electrical and Computer Engineering/ Center for Quantum and Technology, University of Oklahoma, Norman, OK 73019 USA,
##########################################################################
#IMPORTANT_NOTE:
#The code on the left was developed by Sara Aminpour, while the code on the right serves as the reference implementation by Adrián Pérez-Salinas.
#The code on the left has been restructured to handle random data. So some certain sections has been deleted from the reference code.
#Additionally, our code on the left developed to analyze trace distance cost function and linear classification problem
#as well as necessary modification to apply COBYLA, L-BFGS-B, NELDER-MEAD, and SLSQP minimization methods.
##########################################################################


#This file provides useful tools checking how good our results are

from circuitery import code_coords, circuit
from fidelity_minimization import fidelity
from trace_minimization import trace_dis
from weighted_fidelity_minimization import mat_fidelities, w_fidelities
import numpy as np

def _claim(theta, alpha, weight, x, reprs, entanglement, chi):
    """
    This function takes the parameters of a solved problem and one data computes classification of this point

    INPUT:
        -theta: initial point for the theta parameters. The shape must be correct (qubits, layers, 3)

        -alpha: initial point for the alpha parameters. The shape must be correct (qubits, layers, dim)

        -weight: set of parameters needed fot the circuit. Must be an array with shape (classes, qubits)

        -x: coordinates of data for testing.
        -reprs: variable encoding the label states of the different classes
        -entanglement: whether there is entanglement or not in the Ansätze, just 'y'/'n'
        -chi: cost function, to choose between 'fidelity_chi' or 'weighted_fidelity_chi'
    OUTPUT:
        -y_: the class of x, according to the classifier
    """
    chi = chi.lower().replace(' ','_')
    if chi in ['fidelity', 'weighted_fidelity','trace']: chi += '_chi'
    if chi not in ['fidelity_chi', 'weighted_fidelity_chi','trace_chi']:
        raise ValueError('Figure of merit is not valid')

    if chi == 'fidelity_chi':
        y_ = _claim_fidelity(theta, alpha, x, reprs, entanglement)

    if chi == 'trace_chi':
        y_ = _claim_trace(theta, alpha, x, reprs, entanglement)

    if chi == 'weighted_fidelity_chi':
        y_ = _claim_weighted_fidelity(theta, alpha, weight, x, reprs, entanglement)

    return y_


def _claim_fidelity(theta, alpha, x, reprs, entanglement):
    """
    This function is inside _claim for fidelity_chi
    INPUT:
        -theta: initial point for the theta parameters. The shape must be correct (qubits, layers, 3)

        -alpha: initial point for the alpha parameters. The shape must be correct (qubits, layers, dim)

        -weight: set of parameters needed fot the circuit. Must be an array with shape (classes, qubits)

        -x: coordinates of data for testing.
        -reprs: variable encoding the label states of the different classes
        -entanglement: whether there is entanglement or not in the Ansätze, just 'y'/'n'
    OUTPUT:
        the class of x, according to the classifier
    """
    theta_aux = code_coords(theta, alpha, x)
    C = circuit(theta_aux, entanglement)
    Fidelities = [fidelity(r, C.psi) for r in reprs]
    return np.argmax(Fidelities)




#==============================================================================
#==============================================================================
#==============================================================================
#==============================================================================
#==============================================================================

def _claim_trace(theta, alpha, x, reprs, entanglement):
    """
    This function is inside _claim for fidelity_chi
    INPUT:
        -theta: initial point for the theta parameters. The shape must be correct (qubits, layers, 3)
        -alpha: initial point for the alpha parameters. The shape must be correct (qubits, layers, dim)
        -weight: set of parameters needed fot the circuit. Must be an array with shape (classes, qubits)
        -x: coordinates of data for testing.
        -reprs: variable encoding the label states of the different classes
        -entanglement: whether there is entanglement or not in the Ansätze, just 'y'/'n'
    OUTPUT:
        the class of x, according to the classifier
    """
    theta_aux = code_coords(theta, alpha, x)
    C = circuit(theta_aux, entanglement)
    #for r1 in reprs:
    #   Trace=trace_dis(r1, C.r)
    Trace = [trace_dis(r1, C.r) for r1 in reprs]
    #print('td=',Trace)
    #print('reprs[y]=',r1)
    #print('C.r=',C.r)
    #print('min=',np.argmin(Trace))
    return np.argmax(Trace)


#==============================================================================
#==============================================================================
#==============================================================================
#==============================================================================
#==============================================================================



def _claim_weighted_fidelity(theta, alpha, weight, x, reprs, entanglement):
    """
    This function is inside _claim for weighted_fidelity_chi
    INPUT:
        -theta: initial point for the theta parameters. The shape must be correct (qubits, layers, 3)

        -alpha: initial point for the alpha parameters. The shape must be correct (qubits, layers, dim)

        -weight: set of parameters needed fot the circuit. Must be an array with shape (classes, qubits)

        -x: coordinates of data for testing.
        -reprs: variable encoding the label states of the different classes
        -entanglement: whether there is entanglement or not in the Ansätze, just 'y'/'n'
    OUTPUT:
        the class of x, according to the classifier
    """
    theta_aux = code_coords(theta, alpha, x)
    fids = mat_fidelities(theta_aux, weight, reprs, entanglement)
    w_fid = w_fidelities(fids, weight)
    return np.argmax(w_fid)

def tester(theta, alpha, test_data, reprs, entanglement, chi, weights=None):
    """
    This function takes the parameters of a solved problem and one data computes how many points are correct

    INPUT:
        -theta: initial point for the theta parameters. The shape must be correct (qubits, layers, 3)

        -alpha: initial point for the alpha parameters. The shape must be correct (qubits, layers, dim)

        -weight: set of parameters needed fot the circuit. Must be an array with shape (classes, qubits)

        -test_data: set of data for testing
        -reprs: variable encoding the label states of the different classes
        -entanglement: whether there is entanglement or not in the Ansätze, just 'y'/'n'
        -chi: cost function, to choose between 'fidelity_chi' or 'weighted_fidelity_chi'
    OUTPUT:
        -success normalized
    """
    acc = 0
    for i, d in enumerate(test_data):
        x, y = d
        y_ = _claim(theta, alpha, weights, x, reprs, entanglement, chi)
        if y == y_:
            acc += 1
    return acc / len(test_data)


def Accuracy_test(theta, alpha, test_data, reprs, entanglement, chi, weights=None):
    """
    This function takes the parameters of a solved problem and one data computes how many points are correct

    INPUT:
        -theta: initial point for the theta parameters. The shape must be correct (qubits, layers, 3)

        -alpha: initial point for the alpha parameters. The shape must be correct (qubits, layers, dim)

        -weight: set of parameters needed fot the circuit. Must be an array with shape (classes, qubits)

        -test_data: set of data for testing
        -reprs: variable encoding the label states of the different classes
        -entanglement: whether there is entanglement or not in the Ansätze, just 'y'/'n'
        -chi: cost function, to choose between 'fidelity_chi' or 'weighted_fidelity_chi'
    OUTPUT:
        -solutions of the classification
        -success normalized
    """
    dim = len(test_data[0][0])
    solutions = np.zeros((len(test_data), dim + 3)) #data  #Esto se podrá mejorar en el futuro
    for i, d in enumerate(test_data):
        x, y = d
        y_ = _claim(theta, alpha, weights, x, reprs, entanglement, chi)
        solutions[i,:dim] = x
        solutions[i, -3] = y
        solutions[i, -2] = y_
        solutions[i, -1] = int(y == y_)

    acc = np.sum(solutions[:, -1]) / (i + 1)

    return solutions, acc

```

```
##########################################################################
#Quantum classifier
#Adrián Pérez-Salinas, Alba Cervera-Lierta, Elies Gil, J. Ignacio Latorre
#Code by APS
#Code-checks by ACL
#June 3rd 2019


#Universitat de Barcelona / Barcelona Supercomputing Center/Institut de Ciències del Cosmos

##########################################################################


#This file provides useful tools checking how good our results are

from circuitery import code_coords, circuit
from fidelity_minimization import fidelity
from weighted_fidelity_minimization import mat_fidelities, w_fidelities
import numpy as np

def _claim(theta, alpha, weight, x, reprs, entanglement, chi):
    """
    This function takes the parameters of a solved problem and one data computes classification of this point
    INPUT:
        -theta: initial point for the theta parameters. The shape must be correct (qubits, layers, 3)
        -alpha: initial point for the alpha parameters. The shape must be correct (qubits, layers, dim)
        -weight: set of parameters needed fot the circuit. Must be an array with shape (classes, qubits)
        -x: coordinates of data for testing.
        -reprs: variable encoding the label states of the different classes
        -entanglement: whether there is entanglement or not in the Ansätze, just 'y'/'n'
        -chi: cost function, to choose between 'fidelity_chi' or 'weighted_fidelity_chi'
    OUTPUT:
        -y_: the class of x, according to the classifier
    """
    chi = chi.lower().replace(' ','_')
    if chi in ['fidelity', 'weighted_fidelity']: chi += '_chi'
    if chi not in ['fidelity_chi', 'weighted_fidelity_chi']:
        raise ValueError('Figure of merit is not valid')

    if chi == 'fidelity_chi':
        y_ = _claim_fidelity(theta, alpha, x, reprs, entanglement)

    if chi == 'weighted_fidelity_chi':
        y_ = _claim_weighted_fidelity(theta, alpha, weight, x, reprs, entanglement)

    return y_


def _claim_fidelity(theta, alpha, x, reprs, entanglement):
    """
    This function is inside _claim for fidelity_chi
    INPUT:
        -theta: initial point for the theta parameters. The shape must be correct (qubits, layers, 3)
        -alpha: initial point for the alpha parameters. The shape must be correct (qubits, layers, dim)
        -weight: set of parameters needed fot the circuit. Must be an array with shape (classes, qubits)
        -x: coordinates of data for testing.
        -reprs: variable encoding the label states of the different classes
        -entanglement: whether there is entanglement or not in the Ansätze, just 'y'/'n'
    OUTPUT:
        the class of x, according to the classifier
    """
    theta_aux = code_coords(theta, alpha, x)
    C = circuit(theta_aux, entanglement)
    Fidelities = [fidelity(r, C.psi) for r in reprs]

    return np.argmax(Fidelities)


def _claim_weighted_fidelity(theta, alpha, weight, x, reprs, entanglement):
    """
    This function is inside _claim for weighted_fidelity_chi
    INPUT:
        -theta: initial point for the theta parameters. The shape must be correct (qubits, layers, 3)
        -alpha: initial point for the alpha parameters. The shape must be correct (qubits, layers, dim)
        -weight: set of parameters needed fot the circuit. Must be an array with shape (classes, qubits)
        -x: coordinates of data for testing.
        -reprs: variable encoding the label states of the different classes
        -entanglement: whether there is entanglement or not in the Ansätze, just 'y'/'n'
    OUTPUT:
        the class of x, according to the classifier
    """
    theta_aux = code_coords(theta, alpha, x)
    fids = mat_fidelities(theta_aux, weight, reprs, entanglement)
    w_fid = w_fidelities(fids, weight)
    return np.argmax(w_fid)

def tester(theta, alpha, test_data, reprs, entanglement, chi, weights=None):
    """
    This function takes the parameters of a solved problem and one data computes how many points are correct
    INPUT:
        -theta: initial point for the theta parameters. The shape must be correct (qubits, layers, 3)
        -alpha: initial point for the alpha parameters. The shape must be correct (qubits, layers, dim)
        -weight: set of parameters needed fot the circuit. Must be an array with shape (classes, qubits)
        -test_data: set of data for testing
        -reprs: variable encoding the label states of the different classes
        -entanglement: whether there is entanglement or not in the Ansätze, just 'y'/'n'
        -chi: cost function, to choose between 'fidelity_chi' or 'weighted_fidelity_chi'
    OUTPUT:
        -success normalized
    """
    acc = 0
    for i, d in enumerate(test_data):
        x, y = d
        y_ = _claim(theta, alpha, weights, x, reprs, entanglement, chi)
        if y == y_:
            acc += 1

    return acc / len(test_data)


def Accuracy_test(theta, alpha, test_data, reprs, entanglement, chi, weights=None):
    """
    This function takes the parameters of a solved problem and one data computes how many points are correct
    INPUT:
        -theta: initial point for the theta parameters. The shape must be correct (qubits, layers, 3)
        -alpha: initial point for the alpha parameters. The shape must be correct (qubits, layers, dim)
        -weight: set of parameters needed fot the circuit. Must be an array with shape (classes, qubits)
        -test_data: set of data for testing
        -reprs: variable encoding the label states of the different classes
        -entanglement: whether there is entanglement or not in the Ansätze, just 'y'/'n'
        -chi: cost function, to choose between 'fidelity_chi' or 'weighted_fidelity_chi'
    OUTPUT:
        -solutions of the classification
        -success normalized
    """
    dim = len(test_data[0][0])
    solutions = np.zeros((len(test_data), dim + 3)) #data  #Esto se podrá mejorar en el futuro
    for i, d in enumerate(test_data):
        x, y = d
        y_ = _claim(theta, alpha, weights, x, reprs, entanglement, chi)
        solutions[i,:dim] = x
        solutions[i, -3] = y
        solutions[i, -2] = y_
        solutions[i, -1] = int(y == y_)

    acc = np.sum(solutions[:, -1]) / (i + 1)

    return solutions, acc

```

Text Compare

```
# coding=utf-8
########################################################################
#Quantum classifier
#Sara Aminpour, Mike Banad, Sarah Sharif
#September 25th 2024

#School of Electrical and Computer Engineering/ Center for Quantum and Technology, University of Oklahoma, Norman, OK 73019 USA,
#########################################################################
#IMPORTANT_NOTE:
#The code on the left was developed by Sara Aminpour, while the code on the right serves as the reference implementation by Adrián Pérez-Salinas.
#The code on the left has been restructured to handle random data. So some certain sections has been deleted from the reference code.
#Additionally, our code on the left developed to analyze trace distance cost function and linear classification problem
#as well as necessary modification to apply COBYLA, L-BFGS-B, NELDER-MEAD, and SLSQP minimization methods.
#########################################################################


#This file provides the minimization for the cheap chi square
from circuitery import code_coords, circuit
import numpy as np
import random
from scipy.optimize import minimize

def trace_minimization(theta, alpha, train_data, reprs,
                       entanglement, method,
                       batch_size, eta, epochs):
    """
    This function takes the parameters of a problem and computes the optimal parameters for it, using different functions. It uses the trace minimization
    INPUT:
        -theta: initial point for the theta parameters. The shape must be correct (qubits, layers, 3)
        -alpha: initial point for the alpha parameters. The shape must be correct (qubits, layers, dim)
        -train_data: set of data for training. There must be several entries (x,y)
        -reprs: variable encoding the label states of the different classes
        -entanglement: whether there is entanglement or not in the Ansätze, just 'y'/'n'
        -method: minimization method, to choose among ['SGD', another valid for function scipy.optimize.minimize]
        -batch_size: size of the batches for stochastic gradient descent, only for 'SGD' method
        -eta: learning rate, only for 'SGD' method
        -epochs: number of epochs , only for 'SGD' method
    OUTPUT:
        -theta: optimized point for the theta parameters. The shape is correct (qubits, layers, 3)
        -alpha: optimized point for the alpha parameters. The shape is correct (qubits, layers, dim)
        -chi: value of the minimization function
    """

    if method == 'SGD':
        thetas, alphas, chis = _sgd(theta, alpha, train_data, reprs,
                                    entanglement, eta, batch_size, epochs)
        i = chis.index(max(chis))
        return thetas[i], alphas[i], chis[i]

    else:
        params, hypars = _translate_to_scipy(theta, alpha)
        results = minimize(_scipy_minimizing, params,
                           args = (hypars, train_data, reprs, entanglement),
                                   method=method)
        theta, alpha = _translate_from_scipy(results['x'], hypars)

        return theta, alpha, results['fun']


def _gradient(theta, alpha, data, reprs, entanglement):
    """
    This function computes a gradient step for the SGD minimization
    INPUT:
        -theta: initial point for the theta parameters. The shape must be correct (qubits, layers, 3)
        -alpha: initial point for the alpha parameters. The shape must be correct (qubits, layers, dim)
        -data: one data for training. It must be (x,y)
        -reprs: variable encoding the label states of the different classes
        -entanglement: whether there is entanglement or not in the Ansätze, just 'y'/'n'

    OUTPUT:
        -grad_theta: gradient for the theta parameters. The shape is correct (qubits, layers, 3)
        -grad_alpha: gradient for the alpha parameters. The shape is correct (qubits, layers, dim)
        -results['fun']: value of the minimization function
    """

    x,y = data
    theta_aux = code_coords(theta, alpha, x)
    C = circuit(theta_aux, entanglement)
    prod1 = np.dot(np.conj(reprs[y]), C.psi)
    prods2 = np.zeros(theta.shape, dtype='complex')
    (Q, L, I) = theta_aux.shape

    for q in range(Q):
        for l in range(L):
            for i in range(I):
                theta_aux[q, l, i] += np.pi
                der_c = circuit(theta_aux, entanglement)
                prods2[q, l, i] = np.dot(reprs[y], np.conj(der_c.psi))
                theta_aux[q, l, i] -= np.pi
    grad_theta = np.asfarray(np.real(prod1 * prods2))
    if len(x) <= 3:
        dim = len(x)
        grad_alpha = np.empty((theta.shape[0], theta.shape[1], dim))
        for q in range(Q):
            for l in range(L):
                for i in range(dim):
                    grad_alpha[q, l, i] = x[i] * grad_theta[q, l, i]

    if len(x) == 4:
        grad_alpha = np.empty((theta.shape[0], theta.shape[1], 4))
        for q in range(Q):
            grad_alpha[q, l, 0] = x[0] * grad_theta[q, l, 0]
            grad_alpha[q, l, 1] = x[1] * grad_theta[q, l, 1]
            grad_alpha[q, l, 2] = x[2] * grad_theta[q, l, 3]
            grad_alpha[q, l, 3] = x[3] * grad_theta[q, l, 4]


    return grad_theta, grad_alpha


def _train_batch(theta, alpha, batch, reprs, entanglement):
    """
    This function computes a gradient step for a complete batch for the SGD minimization
    INPUT:
        -theta: initial point for the theta parameters. The shape must be correct (qubits, layers, 3)
        -alpha: initial point for the alpha parameters. The shape must be correct (qubits, layers, dim)
        -batch: small set of data for training. It must be several (x,y)
        -reprs: variable encoding the label states of the different classes
        -entanglement: whether there is entanglement or not in the Ansätze, just 'y'/'n'

    OUTPUT:
        -grad_theta: gradient for the theta parameters averaged in batch. The shape is correct (qubits, layers, 3)
        -grad_alpha: gradient for the alpha parameters averaged in batch. The shape is correct (qubits, layers, dim)
    """
    gradient_theta = np.zeros(theta.shape)
    gradient_alpha = np.zeros(alpha.shape)
    for d in batch:
        g_t, g_a = _gradient(theta, alpha, d, reprs, entanglement)
        gradient_theta += g_t
        gradient_alpha += g_a

    return gradient_theta / len(batch), gradient_alpha / len(batch)


def _session_sgd(theta, alpha, train_data, reprs, entanglement, eta, batch_size):
    """
    This function computes a gradient descent step for all batches
    INPUT:
        -theta: initial point for the theta parameters. The shape must be correct (qubits, layers, 3)
        -alpha: initial point for the alpha parameters. The shape must be correct (qubits, layers, dim)
        -train_data: set of data for training. There must be several entries (x,y)
        -reprs: variable encoding the label states of the different classes
        -entanglement: whether there is entanglement or not in the Ansätze, just 'y'/'n'
        -eta: learning rate, only for 'SGD' method
        -batch_size: size of the batches for stochastic gradient descent, only for 'SGD' method

    OUTPUT:
        -theta: updated point for the theta parameters. The shape is correct (qubits, layers, 3)
        -alpha: updated point for the alpha parameters. The shape is correct (qubits, layers, dim)
        -Av_chi_square: value of the minimization function
    """
    batches = [train_data[k:k + batch_size] for k in range(0,
               len(train_data), batch_size)]
    for batch in batches:
        gradient_theta_batch, gradient_alpha_batch = _train_batch(
                theta, alpha, batch, reprs, entanglement)
        theta += eta * gradient_theta_batch #This sign is very important, it is the difference between maximizing or minimizing.
        alpha += eta * gradient_alpha_batch

    return theta, alpha, Av_Tr(theta, alpha, train_data, reprs, entanglement)



def _sgd(theta, alpha, train_data, reprs, entanglement, eta, batch_size, epochs):
    """
    This function completes the whole SGD strategy
    INPUT:
        -theta: initial point for the theta parameters. The shape must be correct (qubits, layers, 3)
        -alpha: initial point for the alpha parameters. The shape must be correct (qubits, layers, dim)
        -train_data: set of data for training. There must be several entries (x,y)
        -reprs: variable encoding the label states of the different classes
        -entanglement: whether there is entanglement or not in the Ansätze, just 'y'/'n'
        -method: minimization method, to choose among ['SGD', another valid for function scipy.optimize.minimize]
        -batch_size: size of the batches for stochastic gradient descent, only for 'SGD' method
        -eta: learning rate, only for 'SGD' method
        -epochs: number of epochs , only for 'SGD' method
    OUTPUT:
        -thetas: optimized points for the theta parameters for all epochs. The shape is correct (qubits, layers, 3)
        -alphas: optimized points for the theta parameters for all epochs. The shape is correct (qubits, layers, dim)
        -chis: value of the minimization function at every step
    """
    thetas = [np.empty(theta.shape)] * epochs
    alphas = [np.empty(alpha.shape)] * epochs
    chis = [0] * epochs
    for e in range(epochs):
        random.shuffle(train_data)
        theta_, alpha_, chi_ = _session_sgd(theta, alpha, train_data, reprs,
                                            entanglement, eta, batch_size)
        thetas[e] = theta_
        alphas[e] = alpha_
        chis[e] = chi_           #Storage for solution

        theta = theta_
        alpha = alpha_           #Next step initialization

    return thetas, alphas, chis



def _translate_to_scipy(theta, alpha):
    """
    This function is a intermediate step for translating theta and alpha to a single variable for scipy.optimize.minimize
    """
    qubits = theta.shape[0]
    layers = theta.shape[1]
    dim = alpha.shape[-1]

    return np.concatenate((theta.flatten(), alpha.flatten())), (qubits, layers, dim)


def _translate_from_scipy(params, hypars):
    """
    This function is a intermediate step for getting theta and alpha from a single variable for scipy.optimize.minimize
    """
    (qubits, layers, dim) = hypars
    if dim <= 3:
        theta = params[:qubits * layers * 3]. reshape(qubits, layers, 3)
        alpha = params[qubits * layers * 3: qubits * layers * 3 + qubits * layers * dim].reshape(qubits, layers, dim)

    if dim == 4:
        theta = params[:qubits * layers * 6]. reshape(qubits, layers, 6)
        alpha = params[qubits * layers * 6: qubits * layers * 6 + qubits * layers * dim].reshape(qubits, layers, dim)
    return theta, alpha



#============================================================================
#Sara
def _scipy_minimizing(params, hypars, train_data, reprs, entanglement):
    """
    This function returns the chi^2 function for using scipy
    INPUT:
        -params: theta and alpha inside the same variable
        -hypars: hyperparameters needed to rebuild theta and alpha
        -train_data: training dataset for the classifier
        -reprs: variable encoding the label states of the different classes
        -entanglement: whether there is entanglement or not in the Ansätze, just 'y'/'n'
    OUTPUT:
        - -Av_Tr, which is the function we want to minimize
    """
    theta, alpha = _translate_from_scipy(params, hypars)
    return -Av_Tr(theta, alpha, train_data, reprs, entanglement)


#Sara
#============================================================================


def trace_dis(r,s):
    """
    This function returns the trace distance of two pure states
    INPUT:
        -2 vectors of pure states of the same dimension
    OUTPUT:
        -trace distance
    """
    dist = np.linalg.norm(r - s)
    td=dist/2

    return td

#============================================================================
def _Tr(theta, alpha, data, reprs, entanglement): #Chi for one point
    """
    This function compute chi^2 for only one point
    INPUT:
        -theta: set of parameters needed for the circuit. Must be an array with shape (qubits, layers, 3)
        -alpha: set of parameters needed for the circuit. Must be an array with shape (qubits, layers, dimension of data)
        -data: one data for training. It must be (x,y)
        -reprs: variable encoding the label states of the different classes
        -entanglement: whether there is entanglement or not in the Ansätze, just 'y'/'n'
    OUTPUT:
        -chi^2 for data
    """
    #
    x, y = data
    #print('data=',data)
    theta_aux = code_coords(theta, alpha, x)
    C = circuit(theta_aux, entanglement)


    '''if y==0:
        s=np.array([0,0,-1])

    elif y==1:
        s=np.array([0,0,1])

    elif y==2:
        s=np.array([1,0,0])

    elif y==3:
        s=np.array([-1,0,0])

    elif y==4:
        s=np.array([0,1,0])

    elif y==5:
        s=np.array([0,-1,0])'''

    ans = trace_dis(reprs[y], C.r)
    return ans

#============================================================================
#Sara
def Av_Tr(theta, alpha, train_data, reprs, entanglement): #Chi in average
    """
    This function compute chi^2 for only one point
    INPUT:
        -theta: set of parameters needed for the circuit. Must be an array with shape (qubits, layers, 3)
        -alpha: set of parameters needed for the circuit. Must be an array with shape (qubits, layers, dimension of data)
        -data: one data for training. It must be (x,y)
        -reprs: variable encoding the label states of the different classes
        -entanglement: whether there is entanglement or not in the Ansätze, just 'y'/'n'
    OUTPUT:
        -Averaged chi^2 for data
    """
    Av_Tr = 0
    for d in train_data:
        Av_Tr += _Tr(theta, alpha, d, reprs, entanglement)

    return Av_Tr / len(train_data)
#Sara
#============================================================================
```
